# lncRNA CYTOR promotes aberrant glycolysis and mitochondrial respiration via HNRNPC-mediated ZEB1 stabilization in oral squamous cell carcinoma

**DOI:** 10.1038/s41419-022-05157-1

**Published:** 2022-08-13

**Authors:** Weiwen Zhu, Jie Wang, Xiang Liu, Yanbin Xu, Rundong Zhai, Jiayi Zhang, Mengqi Wang, Mengyao Wang, Laikui Liu

**Affiliations:** 1 Department of Basic Science of Stomatology, The Affiliated Stomatological Hospital of Nanjing Medical University, Jiangsu, China; 2 Jiangsu Province Key Laboratory of Oral Diseases, Nanjing Medical University, Jiangsu, China; 3 Jiangsu Province Engineering Research Center of Stomatological Translational Medicine, Jiangsu, China

**Keywords:** Cancer metabolism, Cancer metabolism, Oral cancer

## Abstract

Oral squamous cell carcinoma (OSCC), the most common malignancy of the oral and maxillofacial region, severely affects human health. However, current treatments for OSCC commonly show only a ~60% 5-year survival rate of patients with distant metastases, indicating an urgent need for targeted treatments for patients with advanced metastases. Here, we report a survival-related long non-coding RNA, CYTOR, which is highly expressed in the lesions of oral cancer patients. We found that CYTOR can promote both migration and invasion in oral cancer cells as well as the epithelial–mesenchymal transition (EMT). RNA-sequencing of CYTOR-knockdown oral cancer cells revealed that CYTOR can regulate mitochondrial respiration and RNA splicing. Mechanistically, we found that nuclear-localized CYTOR interacts with HNRNPC, resulting in stabilization of ZEB1 mRNAs by inhibiting the nondegradative ubiquitination of HNRNPC. By synthesizing CYTOR-targeting small interfering RNAs (siRNAs) encapsulated in Nanoscale Metal Organic Frameworks (NMOFs), we demonstrate the targeted suppression of CYTOR to inhibit invasion and metastasis of oral cancer cells in a nude mouse model. Cumulatively, this study reveals the potential role of the CYTOR-HNRNPC-ZEB1 axis in regulating mitochondrial metabolism and glycolysis of oral cancer cells, and illustrates the effective use of lncRNA targeting in anti-metastatic cancer therapies.

## Introduction

Oral squamous cell carcinoma (OSCC) is the most common malignancy among head and neck squamous cell carcinomas (HNSCCs). Despite considerable research efforts to establish the causative factors and develop new therapeutic approaches, the overall prognosis for patients with OSCC remains poor, with a 5-year survival rate of only about 60% [1]. The onset of metastasis, the major factor leading to low survival rates among OSCC patients, has become a prominent research concern in studies investigating viable potential treatments for OSCC [2]. Recent publications have revealed that specific signaling mechanisms which promote cancer cell viability [3, 4], immune therapy approaches [5], non-coding RNAs [6], and the epithelial–mesenchymal transition (EMT) [7] all contribute significant effects to distant cancer cell metastasis. Moreover, a better understanding of the different biological characteristics that distinguish distant metastasized cancer cells from primary tumors may provide more potential avenues for cancer treatments [8, 9]. These observations thus emphasize the need for higher resolution capability in distinguishing different cancer cell subpopulations in order to target metastasis in the development of cancer treatments that increase OSCC patient survival rates.

A growing body of evidence supports the likelihood that mitochondria exert a major influence on metabolic reprogramming to drive cancer cell function, and that a mechanistic understanding of mitochondrial function during cancer progression is crucial for development of new cancer therapeutics [8, 10, 11]. Importantly, highly metastatic cancer cell subpopulations can exploit mitochondrial function by inducing elevated oxidative stress via increased reactive oxygen species (ROS) production [8]. Moreover, the “Warburg effect” provides evidence that glycolysis concurrently serves a supporting factor in cancer cell metastasis [12, 13]. Finding potential targets that regulate mitochondrial activity and glycolysis could therefore provide a sound strategy for treating cancer metastasis. ZEB1, a major transcription factor controlling the EMT, has long been recognized as an important regulator of OSCC metastasis [14]. Recent studies have demonstrated that ZEB1 also plays essential roles in regulating processes related to aerobic glycolysis [12, 13]. However, it remains unclear whether and how ZEB1 contributes to regulating mitochondrial reprogramming and aerobic glycolysis in OSCC.

Elevated expression of heterogeneous nuclear ribonucleoprotein C (HNRNPC) has been reported in lung adenocarcinoma, breast cancer, and OSCC patients [15–17]. As an RNA-binding protein (RBP), HNRNPC is well known for its functions in RNA splicing [18], stability [19], and translation [20]. Mechanistically, HNRNPC preferentially binds to five or more single-stranded uridines to affect pre-mRNA stability [21], while the previously reported modified ubiquitination sites on lysine residues located in its in coiled-coil domain contributes to the alternative splicing in virus RNAs [22]. However, the essential mechanism of HNRNPC function in the cancer progression and poor prognosis for patients remains largely unknown.

Long noncoding RNAs (lncRNAs), non-protein coding transcripts longer than 200 nucleotides [23], play a crucial role in tumorigenesis and cancer metastasis. Mechanistically, lncRNAs have been shown to confer significant impacts on gene regulation [24], alternative splicing [25], and post-transcriptional regulation of RBPs [26]. For example, the lncRNA MNX1-AS1 was shown to promote colorectal cancer progression by inhibiting YB1 ubiquitination [27]. lncRNA01232 promotes metastasis of pancreatic cancer by suppressing the ubiquitin-mediated degradation of HNRNPA2B1 [28]. In addition, lncRNA GLCC1 promotes colorectal cancer development through c-Myc mRNA stabilization [29]. Here, we identified a prognosis-related lncRNA, CYTOR, and show its potential regulation of glycolysis and mitochondrial respiration in OSCC. Further investigation demonstrates that the CYTOR-binding to HNRNPC increases stability of ZEB1 mRNAs, thereby promoting mitochondrial respiration and glycolysis. These findings expand our mechanistic understanding of regulatory factors driving distant metastasis in oral cancers, and provide new potential targets for metastatic cancer treatment, demonstrated here by packaging CYTOR-targeting siRNAs into a nanoparticle metal organic frameworks (NMOFs) for OSCC treatment.

## Results

### Upregulation of lncRNA-CYTOR is associated with poor prognosis in OSCC

In order to find prognosis-associated long noncoding RNAs, we screened out 2934 dysregulated lncRNAs from HNSCC patients in the TCGA database (Supplementary Fig. S1A). We additionally used microarray data from OSCC patients (GSE23558) in the Gene Expression Omnibus (GEO) database to narrow the range of candidates, which resulted in the selection of a total of 1533 upregulated genes in OSCC (Supplementary Fig. S1B). Among them, six lncRNAs were discovered to overlap between both datasets (Supplementary Fig. S1C). Assessment of the Kaplan–Meier curves of these six lncRNAs from TCGA using the PrognoScan database and Gene Expression Profiling Interactive Analysis (GEPIA) [30] (Supplementary Fig. S1D) revealed two highly significant, candidate prognosis-related genes, LINC00152 (CYTOR) and HOTAIRM1, for further evaluation. Finally, in order to further validate these candidates, we recruited an independent cohort of 32 OSCC patients consisting of 13 females and 19 males, aged from 40-65, and sampled tumor tissues as well as paired normal epithelial tissues (Supplementary Fig. S1E, F) for qPCR analysis. The results showed that one lncRNA in particular, LINC00152 (CYTOR) was significantly differentially upregulated in OSCC tissues and elevated CYTOR expression was associated with advanced clinical stage (Supplementary Fig. S1G) and advanced pathological grade (Supplementary Fig. S1H) of HNSCC patients in the TCGA database cohort., thus suggesting its potential oncogenic and prognostic value.

**Fig. 1:**
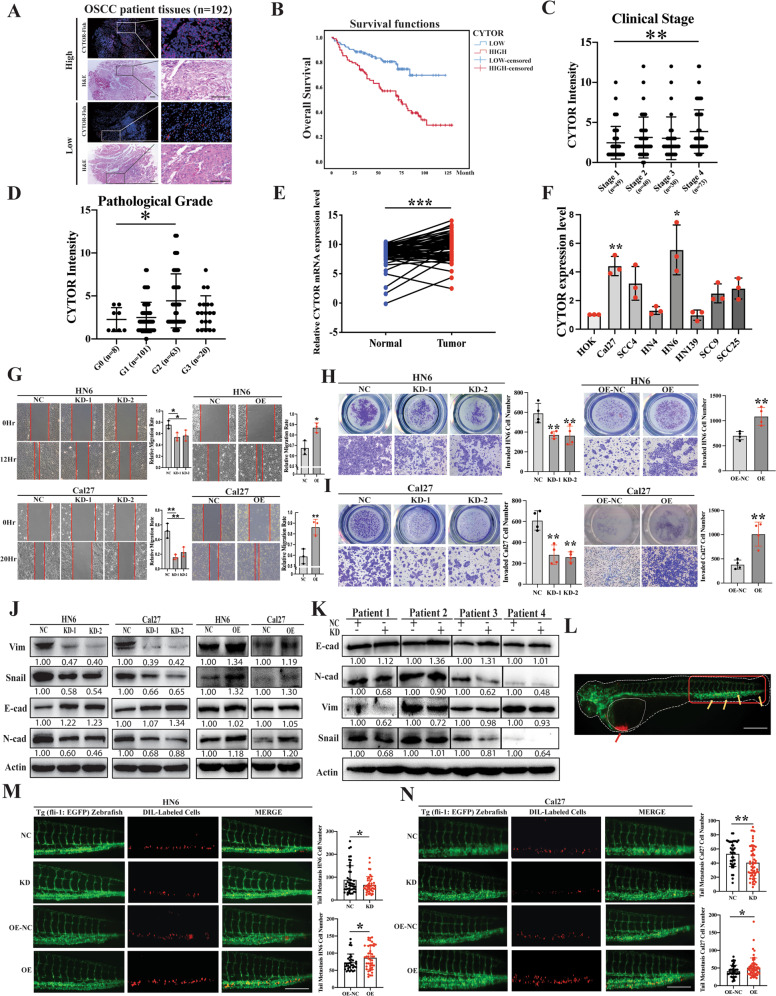
Elevated expression of CYTOR promotes EMT-mediated migration and invasion in OSCC. **A** Total 192 sampled OSCC patient tissues were subjected to tissue microarray for FISH assay. **B** The survival analyses of the CYTOR-low and CYTOR-high expression OSCC patients were performed using the Kaplan–Meier method and assessed using the log-rank test. **C** CYTOR expression in OSCC patients with different clinical stages and **D** pathology grades. **E** qPCR detection showed the CYTOR was highly expressed in OSCC tissue (*n* = 63) compared with the expression in adjacent normal oral epithelium tissue (*n* = 63), statistical analysis was performed by paired *t*-test. **F** Determination of CYTOR mRNA expression in OSCC and normal cell lines. Median values are plotted in the column with data generated from three technical replicates (each dot indicates the average of at least three biological replicates), statistical analysis was performed by one-way anova followed by multiple *t*-tests. **G** Wound healing assays were used to evaluate the expression of CYTOR in regulating the migration behavior of HN6 and Cal27 cell lines. **H**, **I** Transwell invasion assays in CYTOR-knockdown cells and CYTOR-overexpressing cells. Median values are plotted in the column with data generated from four technical replicates (each dot represents the average of three biological replicates), statistical analysis was performed by one-way anova followed by multiple *t*-tests. **J** Western blot assays detected the vimentin, snail, e-cad, n-cad and beta-actin proteins in CYTOR knockdown or overexpression cells. **K** Western blot assays for EMT-related proteins in si-CYTOR transfected PDCs. **L** OSCC cell lines were microinjected into the perivitelline space of zebrafish to evaluate the invasion ability of the cells. Red arrow indicates the injection area of dil-labeled OSCC cells, the red box indicates the monitor region of cell invasion, yellow arrows indicate the metastasized cells. **M**, **N** Zebrafish xenografts revealed that the knockdown of CYTOR inhibited the cells invasion ability, whereas the overexpression of CYTOR promoted the invasion ability of oral cancer cell lines. Median values are plotted in the column, statistical analysis was performed by unpaired *t*-tests. (KD knockdown, NC negative control, OE overexpression; scale bars, 400 μm. The data was presented as mean ± SD, *P < 0.05, **P < 0.01, and ***P < 0.001).

To validate our initial findings, we next recruited a larger group of OSCC patients (*n* = 192), by subjecting the patient samples to tissue microarray. The CYTOR expression was then determined by fluorescence in-situ hybridization (FISH) (Fig. 1A, Supplementary Fig. S2), and we confirmed that higher expression levels of CYTOR were associated with worse outcomes (Fig. 1B) (*P* < 0.001), as well as advanced clinical stage (Fig. 1C) and pathological grade (Fig. 1D). Comparing CYTOR expression in tumors with adjacent noncancerous tissue using qPCR demonstrated that CYTOR was significantly upregulated in OSCC tissue (Normal, 8.339 ± 1.942 vs Tumor, 9.423 ± 2.098; *P* = 0.0002, *n* = 63) (Fig. 1E). Compared its expression in normal oral epithelial cell line HOK, CYTOR was significantly upregulated in OSCC cell lines, especially in two tongue squamous cell carcinoma cell lines (HN6 and Cal27) (Fig. 1F). In addition, FISH imaging assays and RNA subcellular fractionation together showed that CYTOR localized primarily in the nucleus, and to a lesser extent in the cytoplasm of oral cancer cells (Supplementary Fig. S3). Taken together, these results indicated that CYTOR was a strong candidate lncRNA related to OSCC prognosis.

### CYTOR promotes EMT-mediated oral cancer cell migration and invasion

To further assess its function, we established stable CYTOR knockdown (KD) and overexpression (OE) cell lines via lentiviral infection of HN6 and Cal27 cells (Supplementary Fig. S4A–D). 5-Ethynyl-20-deoxyuridine (EdU) and CCK-8 assays showed that changes in the expression of CYTOR did not affect the proliferation of oral cancer cells (Supplementary Fig. S4E–H). Wound healing and transwell assays showed that CYTOR knockdown resulted in decreased migration and invasion behaviors, while CYTOR overexpression resulted in the opposite effect, i.e., elevated migration and invasion, in oral cancer cells (Fig. 1G–I). We then conducted Western blot (WB) and immunofluorescent staining of EMT-related proteins (vimentin, snail, e-cadherin and n-cadherin) to determine the EMT status of the transfected cell lines. The results demonstrated that CYTOR upregulation promotes the EMT process, as indicated by the respective levels of these markers in HN6 and Cal27 OE and KD cell lines (Fig. 1J, Supplementary Fig. S4I). We also found that CYTOR knockdown inhibited the EMT in OSCC patient derived cells (PDCs) (Fig. 1K).

In addition, we used transparent in vivo imaging analysis of tumor metastasis [31, 32] by microinjecting Dil-labeled, stably transfected oral cancer cell lines into the perivitelline space of Tg (fli-1: EGFP) zebrafish for 3 days to observe cell invasion (Fig. 1L). The results indicated that high CYTOR expression promoted the invasion of HN6 (NC, 88.56 ± 61.64 vs KD, 66.53 ± 36.36; *P* = 0.0455, *n* = 34) (OE-NC, 73.29 ± 24.20 vs OE, 87.05 ± 34.45; *P* = 0.0489, *n* = 35) and Cal27 (NC, 53.32 ± 18.08 vs KD, 40.67 ± 22.65; *P* = 0.0052, *n* = 38) (OE-NC, 41.22 ± 16.67 vs OE, 51.20 ± 26.98; *P* = 0.0250, *n* = 50) in zebrafish (Fig. 1M, N). Again, these results were also confirmed in the tail vein metastasis mice model (Supplementary Fig. S5). Collectively, these findings showed that CYTOR could promote EMT, as well as enhanced migration and invasion behavior by oral cancer cells.

### CYTOR positively affects mitochondrial function and aerobic glycolysis in OSCC

**Fig. 2:**
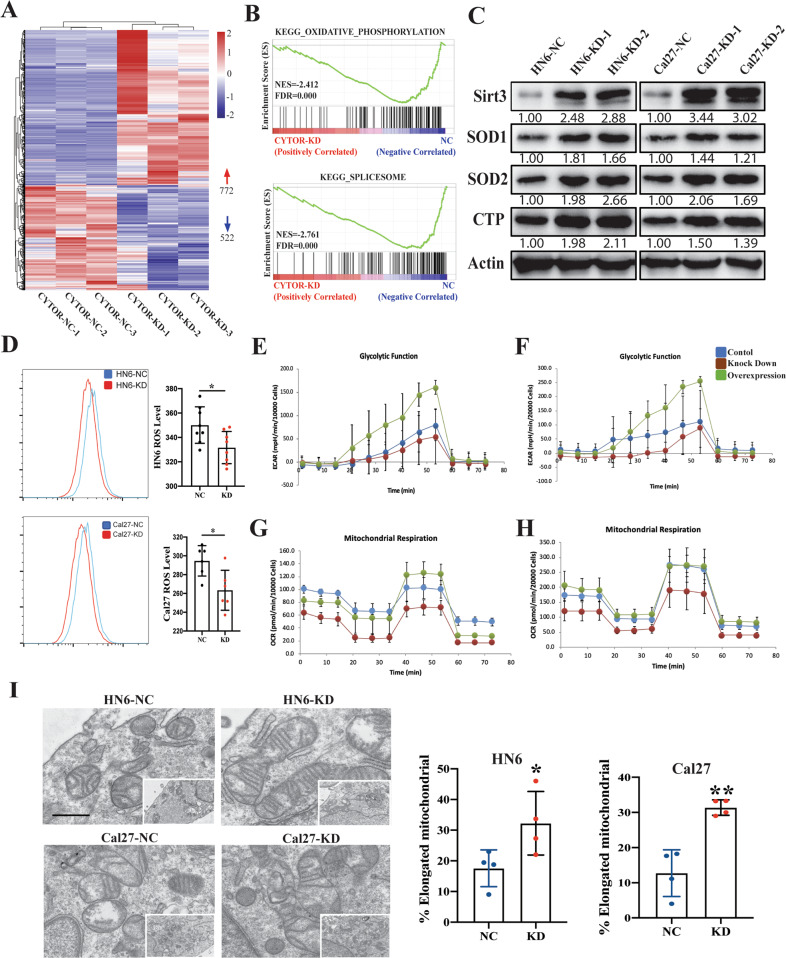
CYTOR regulates the glycolysis and mitochondrial respiration function in oral cancer cells. **A** RNA-seq analysis showing that CYTOR expression is associated with 1294 genes dysregulation in Cal27 cells. **B** Gene set enrichment analysis (GSEA) of the CYTOR-related pathways in CYTOR-knockdown Cal27 cells. **C** Western blot analysis showing the mitochondrial-related proteins (SIRT3, CTP, SOD1, SOD2) expression in CYTOR-depletion cells. **D** Flow cytometry analysis of ROS level (MitoSOX) in HN6 and Cal27 cells. **E**, **F** CYTOR-knockdown and CYTOR-overexpression HN6 and Cal27 cells were detected for ECAR to indicate the glycolysis stress. Median values are plotted in the column with data generated from six technical replicates and three biological replicates (each dot indicates the mean value of three independent biological replicates), statistical analysis was performed by unpaired *t*-tests. **G**, **H** CYTOR-knockdown and CYTOR-overexpression HN6 and Cal27 cells were detected for OCR to indicate the mitochondrial respiration. **I** Transmission electron microscope exposing the ultrastructure of mitochondrial in HN6 and Cal27 cells. Median values are plotted in the dots plot with data generated from four independent biological replicates, statistical analysis was performed by unpaired *t*-tests (KD knockdown, NC negative control; scale bar = 1 μm. The data was presented as mean ± SD, *P < 0.05).

To better understand the effects of CYTOR expression in regulating cellular function and metabolism in OSCC, we conducted RNA-seq analysis of the stable CYTOR KD and negative control (NC, scrambled insert) Cal27 oral cancer cells. The results demonstrated that CYTOR knockdown in these cells led to dysregulation of 1294 genes (772 up- and 522 downregulated) (Fig. 2A, Supplementary Table S1). Gene set enrichment analysis (GSEA) showed that “oxidative phosphorylation” and “spliceosome” were significantly enriched among the CYTOR-associated genes (Fig. 2B), which suggested that CYTOR likely in regulating mitochondrial function and alternative splicing in oral cancer cells. To verify the relationship between CYTOR expression and oxidative phosphorylation, we used WB to detect the accumulation of mitochondrial-related proteins (i.e., Sirt3, CTP, SOD1, and SOD2), which showed that CYTOR suppression led to enhanced expression of these mitochondrial-associated proteins (Fig. 2C). Given their importance as a hallmark of mitochondrial reprograming [8] and aerobic glycolysis [12], we next used flow cytometry to quantify reactive oxygen species (ROS) levels in the KD cell lines and found that ROS production was significantly decreased in these cells compared with controls (Fig. 2D). In addition, we quantified aerobic glycolysis function and mitochondrial by extracellular acidification rate (ECAR) and oxygen consumption rate (OCR), respectively. We observed that ECAR and OCR rates were decreased in CYTOR-silenced cells, but were significantly increased in CYTOR-OE cell lines, compared with controls (Fig. 2E–H), suggesting positive regulatory effects of CYTOR in glycolysis and mitochondrial respiration. Furthermore, transmission electron microscope (TEM) was used to observe mitochondrial ultrastructure, which revealed that silencing of CYTOR apparently drove a shift from circular to elongated morphology in both KD cell lines (Fig. 2I).

### CYTOR modulates the non-degradative ubiquitination of HNRNPC in OSCC

**Fig. 3:**
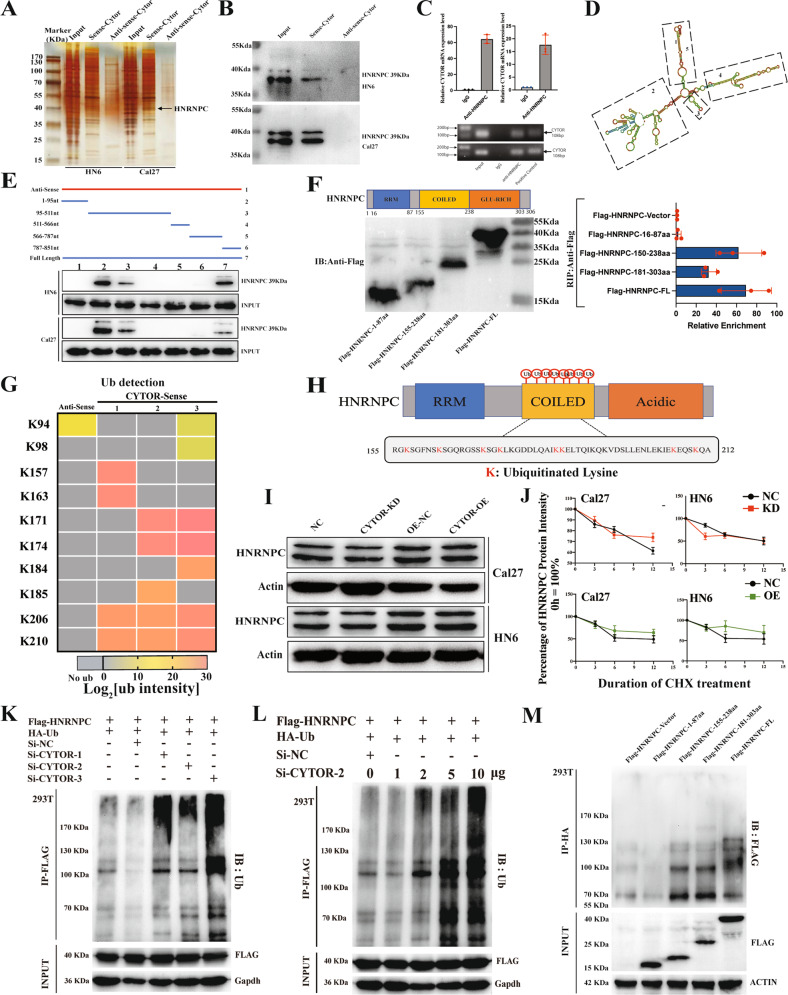
CYTOR inhibits the non-degradative ubiquitination of HNRNPC. **A** Sense and antisense of CYTOR were transcribed, biotinylated in vitro, and incubated with protein extracts from HN6 and Cal27 cells for RNA pull-down assays. The pulled-down proteins were used for silver staining, and a specific band appeared at ~39 kDa (black arrow). **B** Immunoblotting for specific interactions of HNRNPC with CYTOR in HN6 and Cal27 cells. **C** RIP assays were performed using antibody against HNRNPC and normal rabbit IgG. Median values are plotted in the column with data generated from three technical replicates, statistical analysis was performed by unpaired *t*-tests. **D** Graphic illustration of the predicted secondary structure of CYTOR using RNA-fold software. **E** Immunoblotting for HNRNPC in samples pulled down with full-length CYTOR, truncated CYTOR ((1) 1–95 nt; (2) 95–511 nt; (3) 511–566 nt; (4) 566–787 nt; and (5) 787–851 nt) or the antisense of CYTOR. Upper panel: graphic illustration of truncated CYTOR probe according to the secondary structure. Lower panel: immunoblotting for HNRNPC in protein samples pulled down by the different truncated mutants of CYTOR. **F** HNRNPC was truncated (1-87 aa, 155-238 aa, and 181-303 aa) according to its protein domains to identify the specific domain of HNRNPC which interacts with CYTOR. RIP assays were performed to detect the enrichment of CYTOR in cells transfected with full-length and truncated Flag-tagged constructs. Left panel: immunoblotting for truncations of Flag-tagged recombinant HNRNPC protein. Right panel: the enrichment of CYTOR in different truncations were measured by qPCR. Data were generated from three technical replicates. **G** Heatmap of ubiquitinated lysine residues within HNRNPC with normalized log2 abundance of CYTOR pull-down samples. Colors correspond to the intensity of detected ubiquitination by mass spectrometry. **H** Eight ubiquitinated lysine residues (K157, K163, K171, K174, K184, K185, K206, K210) which located within the coiled-coil domain of HNRNPC were detected. **I** Immunoblotting for HNRNPC protein expression in OSCC cells. **J** CYTOR-overexpressing and CYTOR-knockdown HN6 and Cal27 cells were treated with cycloheximide (CHX, 50 μg/mL) for the indicated times. **K**, **L** 293 T cells were transfected with indicated plasmids or small interferon-RNAs and followed by immunoprecipitation (IP), the immunoblotting for HA-Ub showed the ubiquitination of HNRNPC protein. **M** Truncated HNRNPC plasmids were transfected into 293 T cells to evaluate the ubiquitination activity in specific domains. (KD knockdown, NC negative control, OE overexpression; The data was presented as mean ± SD).

To develop a mechanistic understanding of the regulatory effects by which CYTOR contributes to cancer cell metastasis and mitochondrial metabolism, we first conducted pull-down assays with biotinylated sense and anti-sense CYTOR transcripts to identify potential CYTOR-binding proteins in HN6 and Cal27 cells. The precipitated proteins were subjected to silver staining and mass spectrometry analyses. In addition, we selected ten potential RNA-binding proteins for further evaluation based on their predicted subcellular localization to the nucleus by the Human Protein Atlas. Among these, we identified HNRNPC through the presence of specific bands (~39 KDa) in the silver staining assays (Fig. 3A) combined with its occurrence among the most significant CYTOR-related proteins identified by mass spectrometry (MS) (Supplementary Table S2). We therefore hypothesized that HNRNPC may specifically interact with CYTOR to positively regulate cancer cell metabolism. We then re-examined the pull-down products by WB with antibodies against HNRNPC and confirmed that it was a direct binding partner of CYTOR (Fig. 3B). Consistent with this finding, RIP assays followed by agarose gel electrophoresis and qPCR further verified that CYTOR was significantly enriched in the precipitates obtained by an anti-HNRNPC antibody (Fig. 3C). These results further indicated that HNRNPC specifically interacts with CYTOR in oral cancer cells

In order to identify the specific binding domains that interact with HNRNPC protein, we next constructed a series of truncated, biotin-coupled probes for CYTOR based on its predicted secondary structure (Fig. 3D). RNA pull-down assays revealed that CYTOR regions 1–95 nt and 95–511 nt could directly interact with HNRNPC (Fig. 3E). Moreover, RIP assays using full length and truncated, flag-labeled HNRNPC peptides demonstrated that a coiled-coil region (155-238aa) was required for interacting with CYTOR (Fig. 3F).

LncRNAs have been reported to interact with RBPs by regulating the ubiquitination of these proteins [27, 28]. To determine whether CYTOR indeed affected HNRNPC ubiquitination levels, we re-evaluated the MS data of HNRNPC. Interestingly, we noticed that HNRNPC bound to CYTOR also harbored ubiquitin modifications of lysine residues specifically in the coiled-coil region (Fig. 3G, H, Supplementary Fig. S6) (Supplementary Table S3). We then conducted WB analysis of the CYTOR OE and KD cell lines to determine the effects of CYTOR on HNRNPC protein accumulation. The results showed that modulation of CYTOR levels did not significantly affect HNRNPC protein abundance (Fig. 3I). We also performed proteasome degradation assays by treating the CYTOR OE and KD cell lines with CHX and MG-132. Interestingly, the up- or downregulation of CYTOR in these cell lines did not appear to impact the degradation rate of HNRNPC protein compared with that in the controls (Fig. 3J, Supplementary Fig. S7A–C). However, IP assays confirmed that siRNA silencing of CYTOR in 293 T cells led to significantly enhanced ubiquitination of HNRNPC (Fig. 3K, L, Supplementary Fig. S7F, G), the overexpression of CYTOR led to significantly decreased ubiquitination level of HNRNPC (Supplementary Fig. S7H–J). Moreover, the flag-tagged coiled-coil domain was able to pull down a significantly greater amount of ubiquitin molecules than other domains (Fig. 3M). These data indicated that interaction with CYTOR could apparently decrease HNRNPC ubiquitination without significantly affecting its overall abundance.

### CYTOR enhances HNRNPC-mediated ZEB1 mRNA stabilization

**Fig. 4:**
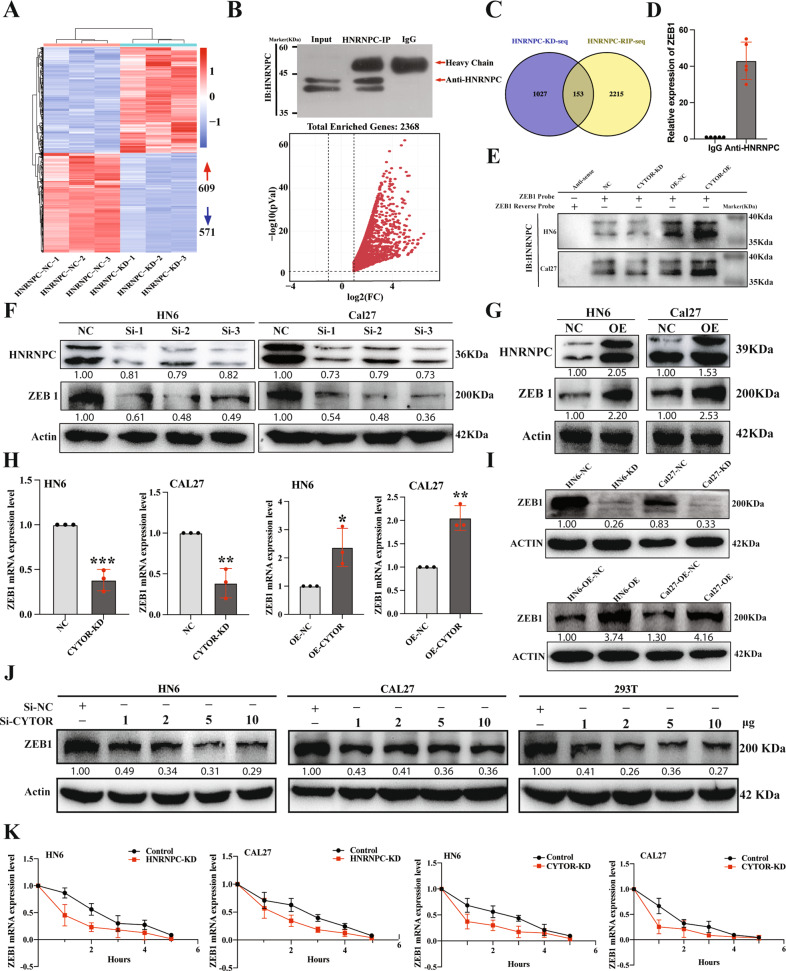
HNRNPC enhances CYTOR-mediated mRNA stability of ZEB1. **A** RNA-seq analysis showing that HNRNPC expression is associated with 1180 genes dysregulation in Cal27 cells. **B** RIP-seq assay demonstrated that 2368 protein-coding genes possibly interact with HNRNPC; Upper panel: IP result of anti-HNRNPC antibody; Lower panel: Volcano plot showed 2368 protein-coding genes significantly enriched in the precipitates obtained by an anti-HNRNPC antibody. **C** Venn diagram showed 153 genes in both RNA-seq and RIP-seq analysis. **D** RIP assays were performed using antibody against HNRNPC and normal rabbit IgG to verify the interactions of ZEB1 and HNRNPC. Median values are plotted in the column with data generated from five technical replicates, each dot indicates the average of the three independent biological replicates. **E** The protein extracts of CYTOR-knockdown and CYTOR-overexpression cell lines were incubated with the biotin labeled-sense and anti-sense of ZEB1 for pull-down assays then immunoblotted for HNRNPC. **F** HN6 and Cal27 cells were transfected with small interferon RNA of HNRNPC and subjected to WB to evaluate the protein expression of ZEB1 and HNRNPC. **G** Cells were transfected with pcDNA-HNRNPC and pcDNA-NC to evaluate the effect of HNRNPC in regulating the ZEB1 expression. **H** qPCR analysis for ZEB1 expression in CYTOR-knockdown and CYTOR-overexpression cell lines. Median values are plotted in the column with data generated from three technical replicates, each dot represents the average gene expression level from at least three biological replicates. Statistical analysis was performed by unpaired *t*-tests. **I** Immunoblotting for ZEB1 expression in CYTOR-knockdown and CYTOR-overexpression cell lines. **J** HN6, Cal27 and 293 T cells were transfected with the different doses of si-RNAs to knockdown the CYTOR expression, then the expression of ZEB1 was analysis by WB. **K** Cells were treated with actinomycin D for the indicated time, then mRNA expressions of ZEB1 were measured by qPCR. (Linear mixed effect regression models with time point defined as random intercept and group defined as random slope were used to test the difference between two groups, median values are plotted in the plots with data generated from three technical replicates and three biological replicates). (*P* < 0.05).

HNRNPC, a spliceosome component, is well-established to participate in pre-mRNA splicing and mRNA stabilization [33]. To better understand the HNRNPC downstream regulation, we first conducted RNA-seq analysis of stable HNRNPC KD and negative control (NC, scrambled insert) oral cancer cells (Fig. 4A), which identified that HNRNPC-KD resulted in dysregulation of 1180 genes (609 up- and 571 downregulated) (Supplementary Table S4). We then conducted RNA immunoprecipitation followed by high-throughput sequencing (RIP-seq) to identify the transcripts interacting with HNRNPC (Fig. 4B), which resulted in the detection of 2368 potential protein-coding genes (Supplementary Table S5). Among them, 153 genes were discovered to overlap between RNA-seq data from HNRNPC-KD cell lines and RIP-seq (Fig. 4C). However, only 19 mRNAs were identified with verified experimental evidence from ENCODE eCLIP data [34]. This data in combination with calculations from the RNA-Protein Interaction Prediction (RPISeq) software (Supplementary Table S6) indicated that ZEB1 and LIN28B were the two genes with the highest possibility to interact with HNRNPC. We further plot the predicted binding matrix map of both two genes in CatRAPID, interestingly, ZEB1 was predicted to bind with HNRNPC in a broader interaction region than LIN28B (Supplementary Fig. S9). Also, given that HNRNPC was reported to interact with ZEB1 to stabilize its mRNAs in esophageal cancer [35], we therefore hypothesized that HNRNPC could possibly directly interact with ZEB1 mRNAs to prolong their stability in oral cancer cells. To explore this possibility, we initially used RIP assays to confirm that ZEB1 mRNA was significantly enriched in the precipitates obtained by anti-HNRNPC antibody (Fig. 4D). Subsequent pull-down assays were then used to determine whether and how CYTOR impacted interactions between HNRNPC and ZEB1 in the stable KD and OE cell lines. The results showed that binding activity between HNRNPC and ZEB1 mRNA was enhanced in the OE lines, but decreased in the KD lines, which indicated that CYTOR participated in interactions between HNRNPC and ZEB1 mRNA (Fig. 4E). Next, we transfected the HN6 and Cal27 oral cancer cell lines with small interfering RNAs or a pcDNA plasmid to knock down or overexpress HNRNPC, respectively. Analysis by WB to detect the ZEB1 protein expression in these lines demonstrated that upregulation of HNRNPC resulted in higher ZEB1 accumulation, while its silencing decreased ZEB1 levels, thereby showing that HNRNPC positively regulated ZEB1 expression (Fig. 4F, G).

Additionally, we evaluated ZEB1 transcription levels in the stable CYTOR OE and KD HN6 and Cal27 cell lines using qPCR and observed that CYTOR silencing led to attenuated ZEB1 mRNA expression, while its overexpression resulted in significantly higher ZEB1 mRNA levels (Fig. 4H). Subsequent WB assays confirmed that CYTOR expression levels shared a positive relationship with ZEB1 protein accumulation (Fig. 4I), and si-CYTOR treatment attenuated ZEB1 protein expression in a dose-dependent manner in both oral cancer cells and 293 T cells (Fig. 4J). Next, we used actinomycin D, a well-known inhibitor of mRNA stability, to evaluate the lifespan of ZEB1 mRNAs. The results showed that deletion of either CYTOR or HNRNPC promoted the degradation of ZEB1 mRNAs in oral cancer cells (Fig. 4K). Collectively, these data indicated that CYTOR could affect the ability of HNRNPC to interact with ZEB1, further enhancing ZEB1 mRNA stability in oral cancer cells.

### ZEB1 enhances mitochondrial respiration and aerobic glycolysis in OSCC

**Fig. 5:**
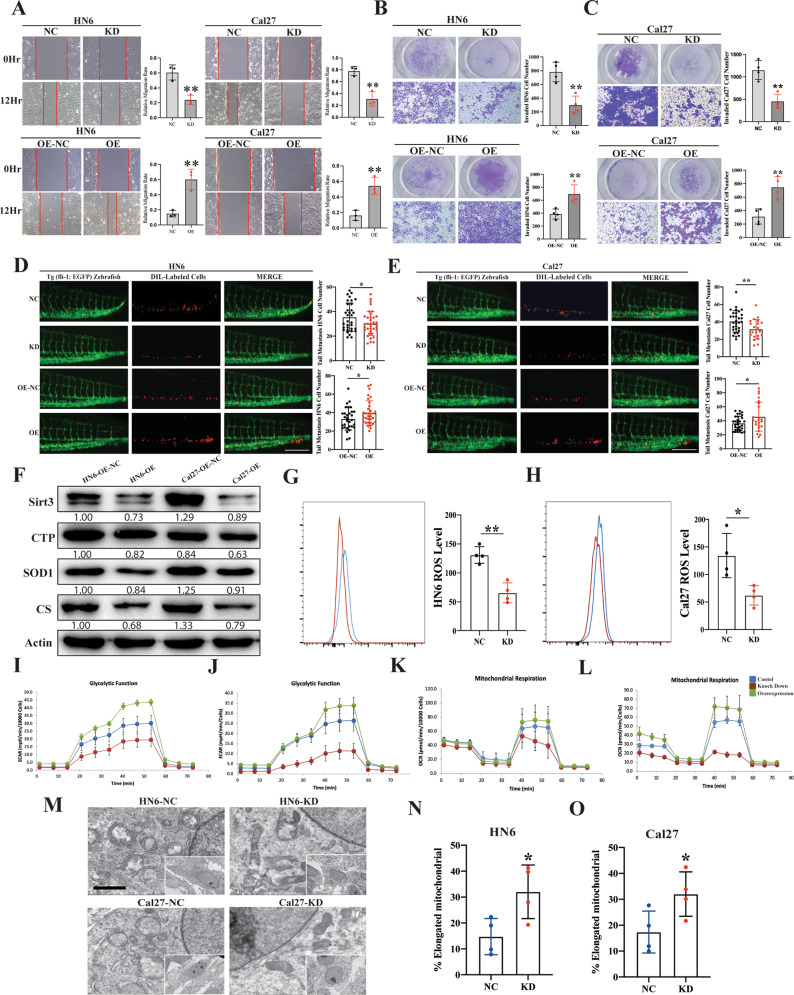
ZEB1 positively regulates the mitochondrial respiration and aerobic glycolysis mediated EMT of OSCC. **A** Wound healing assays were used to evaluate the expression of ZEB1 in regulating the migratory ability of HN6 and Cal27 cell lines. **B**, **C** Transwell invasion assays in ZEB1-knockdown cells and ZEB1-overexpressing cells. Median values are plotted in the column with data generated from at least three technical replicates, statistical analysis was performed by unpaired *t*-tests. **D**, **E** Zebrafish xenografts revealed that the knockdown of ZEB1 inhibited the cells invasion ability, whereas the overexpression of ZEB1 promoted the invasion ability of oral cancer cell lines. Median values are plotted in the column, statistical analysis was performed by unpaired *t*-tests. **F** WB analysis showing the mitochondrial-related proteins (SIRT3, CTP, SOD1, Citrate Synthase) expression in ZEB1-overexpression cells. **G**, **H** Flow cytometry analysis of ROS level (MitoSOX) in HN6 and Cal27 cells. Median values are plotted in each dots plot, the data were generated from four technical replicates, and the statistical analysis was performed by unpaired *t*-tests **I**, **J** ZEB1-knockdown and ZEB1-overexpression HN6 and Cal27 cells were detected for ECAR to indicate the glycolysis stress. **K**, **L** ZEB1-knockdown and ZEB1-overexpression HN6 and Cal27 cells were detected for OCR to indicate the mitochondrial respiration. **M**–**O** Transmission electron microscope exposing the ultrastructure of mitochondrial in HN6 and Cal27 cells. Median values are plotted in the column with data generated from four technical replicates, statistical analysis was performed by unpaired *t*-tests (KD knockdown, NC negative control, OE overexpression, CS Citrate Synthase. The data was presented as mean ± SD, *P < 0.05, **P < 0.01. Each dot showed in the dot plots represents the average of at least three independent biological replicates).

To investigate the role of ZEB1 in OSCC, we next generated HN6 and Cal27 stable ZEB1 KD and OE cell lines via lentiviral infection (Supplementary Fig. S10) to evaluate its effects on oral cancer cell migration and invasion. The results showed that ZEB1 expression was positively associated with oral cancer cell migration (Fig. 5A) and invasion (Fig. 5B, C) levels. Cell metastasis assays in zebrafish, performed using the same method as above, showed that ZEB1 overexpression promoted metastasis, while its suppression had inhibitory effects in HN6 (NC, 35.39 ± 11.49 vs KD, 30.17 ± 9.759; *P* = 0.0450, *n* = 33) (OE-NC, 33.16 ± 12.12 vs OE, 39.61 ± 13.14; *P* = 0.0461, *n* = 31) and Cal27 tumors (NC, 40.52 ± 12.21 vs KD, 31.43 ± 11.31; *P* = 0.0092, *n* = 21) (OE-NC, 36.32 ± 10.08 vs OE, 45.83 ± 20.59; *P* = 0.0295, *n* = 23) in zebrafish (Fig. 5D, E).

Further, using WB analysis we found that overexpression of ZEB1 significantly inhibited the expression of mitochondrial metabolism-related proteins (i.e., Sirt3, CTP, SOD1, and CS) (Fig. 5F). Flow cytometry assays also demonstrated that ZEB1 silencing led to significantly decreased ROS production in HN6 and Cal27 cells (Fig. 5G, H). Evaluation of aerobic glycolysis and mitochondrial respiratory function in these ZEB1 KD and OE cell lines showed that ECAR and OCR rates were significantly higher under ZEB1 overexpression and significantly lower upon ZEB1 knockdown compared to control oral cancer cells (Fig. 5I–L). Mitochondrial ultrastructure analysis by TEM also demonstrated that the ZEB1 silencing led to their morphological elongation in cancer cells (Fig. 5M–O). Meanwhile, by transfecting the pcDNA 3.1 plasmids to CYTOR-KD cell lines for ZEB1 overexpression, we demonstrated that overexpression of ZEB1 significantly alleviates the CYTOR-KD induced inhibition of migration and invasion (Supplementary Fig. S11A–D). Moreover, the ZEB1 overexpression also reversed the CYTOR-KD induced aberrant changes in EMT and mitochondrial function (Supplementary Fig. S11E, F). The findings relating to aerobic glycolysis and mitochondrial respiratory function in these cell lines demonstrated that ZEB1 overexpression significantly rescued CYTOR-KD induced inhibition of ECAR and OCR rates (Supplementary Fig. S11G–J).

### SIRT3 and COX10 are targets of ZEB1 transcriptional regulation

**Fig. 6:**
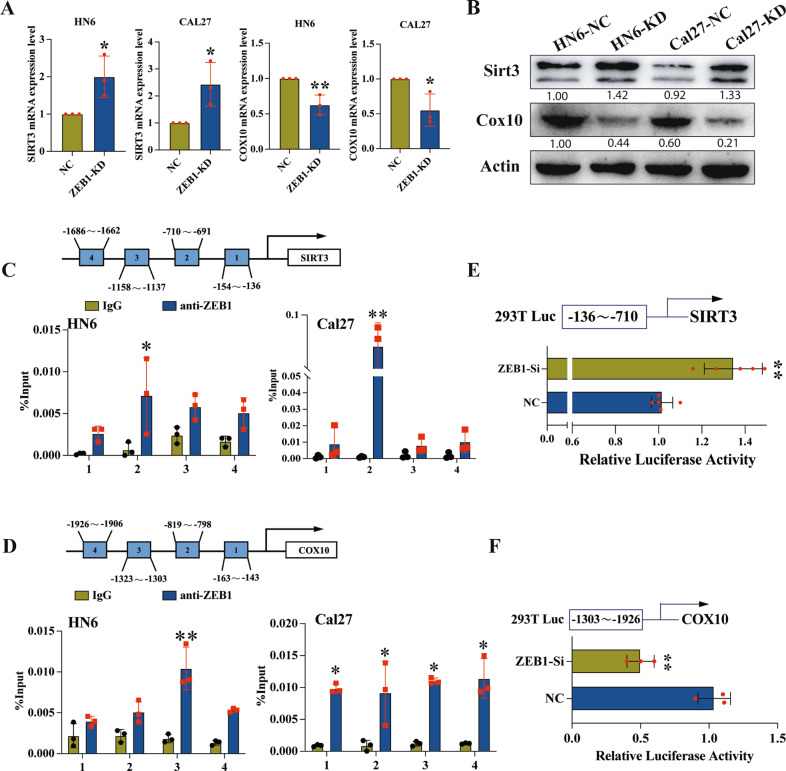
SIRT3 and COX10 are transcription targets of ZEB1. **A** Representative qPCR analysis of SIRT3 and COX10 expression in ZEB1-depletion cell lines. **B** Immunoblotting for SIRT3 and COX10 in ZEB1-knockdown cells. **C**, **D** Four primers were designed to cover the human SIRT3 or COX10 promoter regions and were used to identify ZEB1 binding sites in ChIP assays. **E** Luciferase reporter assay indicated that the luciferase activity was significantly increased in ZEB1-depletion cells containing the reporters with binding site of SIRT3. **F** The luciferase activity was significantly decreased in ZEB1-depletion cells containing the reporters with binding site of COX10. (Median values are plotted in the column with data generated from at least three independent biological replicates, statistical analysis was performed by unpaired *t*-tests. KD knockdown, NC negative control, Si small interferon. The data was presented as mean ± SD, *P < 0.05, **P < 0.01).

To further explore mechanism by which ZEB1 could regulate mitochondrial metabolism and glycolysis, we screened the Gene Transcription Regulation Database (GTRD) for genes which were transcriptionally regulated by ZEB1. This analysis showed that the promoter regions of SIRT3, a negative regulator of glycolysis [12], and COX10, the regulator of mitochondrial respiration [36] contained putative ZEB1 binding sites. We therefore evaluated SIRT3 and COX10 gene expression in ZEB1 KD cells and found that ZEB1 deficiency resulted in significantly higher SIRT3 expression but significantly inhibited COX10 expression than that in control cells, at both the mRNA and protein levels (Fig. 6A, B). To confirm that ZEB1 could physically bind to the SIRT3 and COX10 promoter regions in oral cancer cells, we conducted ChIP and Dual-luciferase reporter assays. The results showed that the SIRT3 and COX10 promoter regions were both significantly enriched with ZEB1 (Fig. 6C, D). Furthermore, the dual-luciferase assays indicated that ZEB1 depletion promoted SIRT3 promoter-driven luciferase activity but inhibited COX10 promoter luciferase activity (Fig. 6E, F). Taken together, these results showed that ZEB1 could negatively regulate SIRT3 but activate COX10 transcription as part of its function in OSCC.

### The CYTOR-HNRNPC-ZEB1 axis is a potential therapeutic target for treating OSCC

**Fig. 7:**
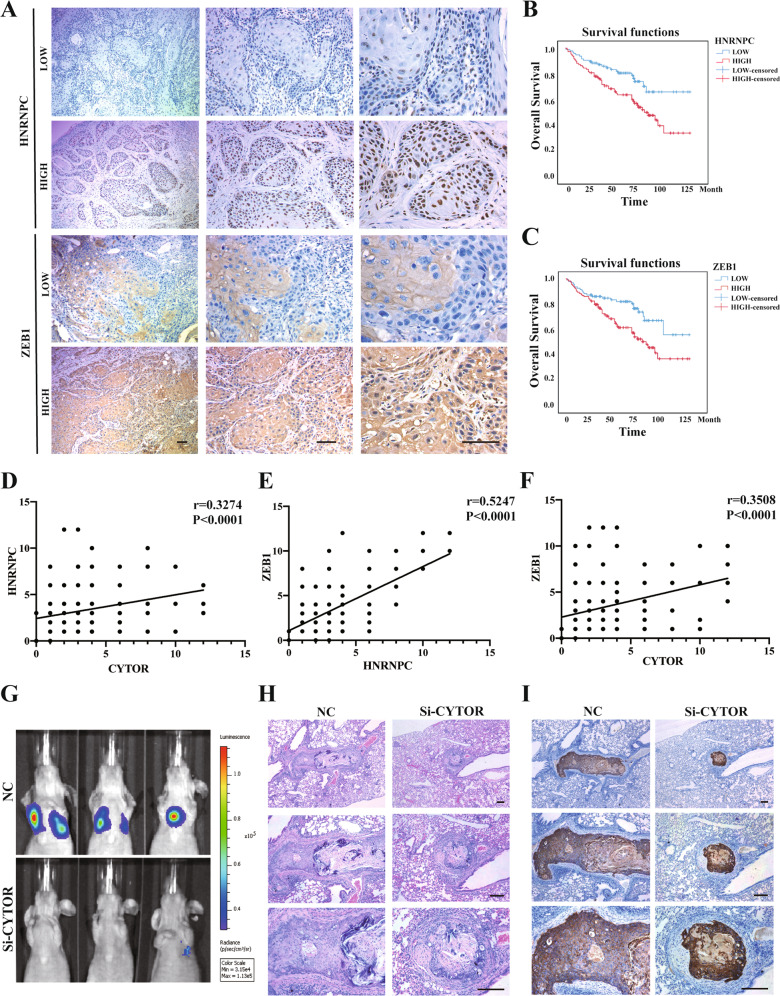
CYTOR-HNRNPC-ZEB1 axis is a potential therapeutic target for treating OSCC. **A** HNRNPC and ZEB1 expression in specimens of OSCC patients. (*n* = 192) **B** Kaplan–Meier analysis of OS curves for OSCC patients with HNRNPC-low and HNRNPC-high expression. **C** OS curves for OSCC patients with ZEB1-low and ZEB1-high expression. **D** Correlations between CYTOR and HNRNPC, **E** HNRNPC and ZEB1, and **F** CYTOR and ZEB1 were determined by Spearman’s correlation assay. **G** Nude mice were injected with the luciferase-labeled oral cancer cells, si-CYTOR encapsuled NMOFs (UiO-66-NH_2_) were injected at day 21 post-injection of cancer cells. Lung-metastasized cells were measured at day 45 post-injection. **H** Representative images of H&E staining of lung-metastasized cancer cells. **I** Representative images of IHC staining of cytokine in lungs.

In order to explore whether the CYTOR-HNRNPC-ZEB1 axis can serve as a target for OSCC therapeutic interventions, we first evaluated the expression of HNRNPC and ZEB1 proteins in 192 primary OSCC samples using IHC (Fig. 7A). The results revealed significant correlations between HNRNPC expression levels and tumor infiltration (*P* = 0.019) and recurrence (*P* < 0.001), while ZEB1 expression was significantly correlated with recurrence (*P* < 0.001). However, neither HNRNPC nor ZEB1 expression showed any correlations with gender, age, or tumor location (Supplementary Table S7). Nevertheless, the elevated expression levels of HNRNPC and ZEB1 were also associated with the poor overall survival rates (OS) (*P* < 0.001) (Fig. 7B, C) of OSCC patients, which supported the potential therapeutic value of targeting HNRNPC and ZEB1 expression for OSCC treatment. Furthermore, combining relative FISH expression scores for CYTOR and IHC scores of HNRNPC and ZEB1 in this cohort (*n* = 192), we observed significant positive correlations between CYTOR and HNRNPC (Fig. 7D), HNRNPC and ZEB1 (Fig. 7E), and CYTOR and ZEB1 (Fig. 7F).

In light of these findings, we then explored the Enhanced Permeability and Retention (EPR) effects of nanoparticle accumulation at tumor sites [37, 38] by synthesizing nanoscale metal-organic frameworks (NMOFs) to package siRNAs targeting CYTOR for suppression in an in vivo OSCC mouse model (Supplementary Fig. S12). We then assessed whether these tumor-targeting NMOFs could inhibit metastasis of oral cancer cells in a nude mouse tail vein metastasis model. After injection of NMOFs (containing CY5-labbled siRNA) for three consecutive weeks, we observed a significant inhibition of OSCC metastasis to lungs in the siCYTOR group compared with that in the siNC group (Fig. 7G). Consistent with the results of H&E staining (Fig. 7H), IHC-based detection of metastasized oral cancer cells showed a significant decrease in siCYTOR-treated mice (Fig. 7I). Taken together, these findings indicate that targeting the CYTOR-HNRNPC-ZEB1 axis may serve as a robust and effective treatment strategy against OSCC.

## Discussion

Aberrant energy metabolism in cancer cells represents a major, common hallmark of cancer [39]. Glycolysis, the main process by which tumor cells generate energy [40], is well-established to contribute to both tumor progression and EMT [41]. In addition to glycolysis, controversy has long persisted as to whether mitochondrial respiration is also a contributing factor in tumor development [8]. In fact, mitochondrial function is critical for cancer development [10], and especially so for the viability of metastasized cells [42]. As a major contributor to the production of cellular ROS, mitochondrial reprogramming can drive cancer cell metastasis via excessive ROS production [43]. Our findings here and in previous studies of cancer cell energy metabolism led us to propose that the CYTOR lncRNA can act as a regulator of both mitochondrial respiration and glycolysis in OSCC (Fig. 2). In this study, we show that CYTOR regulates ZEB1 via HNRNPC-mediated mRNA stabilization to alter energy metabolism, ultimately facilitating metastasis of oral cancer cells (Figs. 3, 4). To our knowledge, we provide the first demonstration of an etiological role for the CYTOR-HNRNPC-ZEB1 axis in regulating both oxidative stress and glycolytic processes in oral cancer invasion. Further studies are needed to investigate different possible modes of CYTOR targeting (and consequently, energy metabolism) for anti-cancer therapies.

Metastasis-targeting therapies have recently garnered considerable research attention due to their substantial contributions towards improving prognoses of cancer patients [44]. However, identifying distinct features of metastasized tumors from those of primary tumors can provide a better understanding of the targetable characteristics unique to the metastasized subgroup of cancers [8, 45]. Due to limitations of conventional cancer treatments, improving the therapeutic efficacy of treatments for metastasized cells remains largely unexplored. Given the enhanced biodistribution of chemotherapeutic agents (i.e., accumulation at the tumor site) conferred by EPR effects, this platform represents a viable strategy for targeting metastasized tumor sites [46]. In this work, NMOFs were used to package siRNAs for metastasis-targeted treatments. Notably, the administered CY5-labeled siRNAs were primarily enriched in metastasized lung tumor sites (Supplementary Fig. S12H), possibly due to the small NMOF particle diameter and EPR effects, resulting in obvious inhibition of lung metastasis (Fig. 7G–I). These experiments provide strong evidence supporting the use of a targeted delivery strategy for treatment of metastatic cancers.

The ZEB1 transcription factor is a primary regulator of EMT [47]; high ZEB1 expression levels have been shown to contribute to development of several cancers and serve as an apparent predictor of poor overall survival of cancer patients [48]. ZEB1 was also shown to regulate enzymes required for aerobic glycolysis activity [13]. In our study, we demonstrated that ZEB1 can promote both glycolysis and oxidative stress by regulating the expression of SIRT3 and COX10 (Fig. 5F–O, Fig. 6). As a differentially upregulated gene in multiple tumor types or cancer cell lines, HNRNPC can promote cancer development [33] and mediate RNA splicing [18, 49]. Recent studies using adenovirus-mediated aberrant RNA and protein binding showed that the non-degradative ubiquitination of HNRNPC can affect the splicing of viral RNA [22]. Our findings are in agreement with these previous studies, and further expand our understanding of this regulatory mechanism by showing that interactions between CYTOR and HNRNPC can significantly impact HNRNPC ubiquitination levels (Fig. 3G–M). Our further investigation of HNRNPC interactions with ZEB1 demonstrate that CYTOR-binding negatively affects HNRNPC ubiquitination, consequently leading to increased interactions between HNRNPC and ZEB1 mRNAs (Fig. 4E). Furthermore, our results show that the enhanced interactions between HNRNPC and ZEB1 mRNAs significantly prolong ZEB1 mRNA stability (Fig. 4K). Also, we demonstrated that HNRNPC significantly promotes the migration and invasion of oral cancer cells (Supplementary Fig. S8). These cumulative findings provide strong evidence that the CYTOR-HNRNPC-ZEB1 axis regulates energy metabolism in the development of tumor metastasis.

## Conclusions

**Fig. 8:**
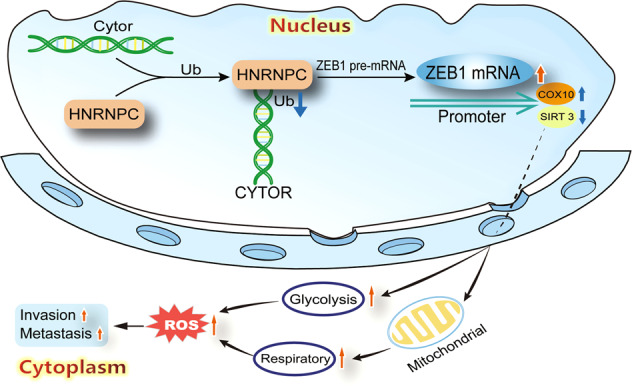
Schematic diagram of CYTOR-HNRNPC-ZEB1 axis regulates the energy metabolism-mediated metastasis of oral cancer cells. LncRNA CYTOR enhances ZEB1 expression via HNRNPC-mediated mRNA stabilization to alter energy metabolism, ultimately facilitating metastasis and invasion of oral cancer cells.

In the present study, we identified the lncRNA, CYTOR, which is expressed at significantly elevated levels in HNSCC patients and is specifically correlated with poor clinical outcomes. We first demonstrated that CYTOR enhance the migration and invasion capability of OSCC cells. RNA-seq of CYTOR KD oral cancer cell lines with GSEA and pathway enrichment analysis indicated that CYTOR was involved in mitochondrial functions and alternative RNA splicing, while further studies confirmed that CYTOR can positively regulate oxidative stress and glycolysis activity in these cell lines. Therefore, we have demonstrated for the first time that CYTOR-HNRNPC-ZEB1 axis promotes the migration and invasion of oral cancer cells through regulating mitochondrial metabolism and glycolysis (Fig. 8). By packaging CYTOR-targeting siRNAs into NMOFs, we provide a powerful example of a nanoscale tumor-specific drug delivery strategy that confers anti-metastatic effects through targeting a lncRNA.

## Materials and methods

### Patients

Total of 192 patients with primary OSCC were recruited from 2009 to 2015 and treated by surgery at the Department of Oral and Maxillofacial Surgery, the Affiliated Stomatological Hospital of Nanjing Medical University. Tumor pathological grade was classified according to WHO classification criteria. Clinical stage and TNM stage were defined by the UICC and the American Joint Commission on Cancer (AJCC). This study was approved by the Nanjing Medical University Ethics Committee and was in accordance with the World Medical Association Declaration of Helsinki, and written informed consent was obtained from all patients.

### Zebrafish metastasis model

To evaluate the impact of CYTOR and ZEB1 genes expression on oral cancer cell metastasis ability. The Tg (fli-1: EGFP) zebrafish was used to observe the cancer cells invasion. Fertilized zebrafish eggs were collected at 28 °C, and embryos at 24 h post fertilization (hpf) were treated with 0.2 mM N-phenylthiourea (PTU; Sigma) to prevent pigmentation. To avoid bias affecting the selection of zebrafish, we also randomized the embryos. Zebrafish embryos were anaesthetized with 0.003% tricaine (Sigma-Aldrich, St. Louris, MO, USA) and placed on a 10 cm Petri dish coated with 1% agarose at 48 hpf. After labeled the tumor cells with 2 g/ml of DiI (Fluka, Germany), the single cell suspension, formulated into certain density of 2 × 10^7^ cells per milliliter, was injected into perivitelline space (PVs) by microinjection. Each embryo beard about 200 cells to cause tail metastasis. Suitable injected embryos were selected after 2 h post injection (hpi). Selected embryos were placed in 32 °C for subsequent experiments. There were no inclusion or exclusion criteria used in the selection of the animals. Tumor cells migration in caudal haematopoietic tissue (CHT) were monitored (blinded outcome asssement) for three consecutive days by an inverted fluorescence microscope (IX71, Olympus, Japan).

### Nude mice metastasis model

BALB/c nude mice (4–6 weeks old, female) were purchased from Vital River Laboratory Animal Technology Co. Ltd (Beijing, China) and raised under specific pathogen-free conditions in the Animal Core Facility of Nanjing Medical University. There were no inclusion or exclusion criteria used in the selection of the animals. Animals from each cage were randomly allocated to the control or treated groups, but no blinding was used. All experimental procedures were approved by the Animal Ethics and Welfare Committee of Nanjing Medical University. Total 1 × 10^6^ Cal27 cells were administrated via tail vein to induce the metastasis. NMOFs (siRNA@UiO-66-NH_2_) were injected two times per week from day 21 to day 45. On day 45 the lung-metastasized cells were observed by fluorescence imaging (blinded outcome asssement). The lung samples were then harvested for histological analysis.

### Statistical analysis

Data were checked for a normal distribution, by the Kolmogorov–Smirnov test, all the normally distributed continuous variables were described as mean ± SD. One-way anova and Student’s paired or unpaired *t*-tests were used for the comparison of significant differences between groups with GraphPad Prism software (GraphPad Prism, RRID: SCR_002798). For all experiments, measures were performed from at least three technical replicates and experiments were repeated at least three times (biological replicates), and each dot from the dot plots indicates the average of at least three independent biological replicates. Survival analyses were performed using the Kaplan–Meier method and assessed using the log-rank test with SPSS software (SPSS, RRID: SCR_002865). The levels of significance were set to *P < 0.05, **P < 0.01, ***P < 0.001, and ****P < 0.0001.

## Supplementary information

Supplementary Materials

Supplementary Figure 1

Supplementary Figure 2

Supplementary Figure 3

Supplementary Figure 4

Supplementary Figure 5

Supplementary Figure 6

Supplementary Figure 7

Supplementary Figure 8

Supplementary Figure 9

Supplementary Figure 10

Supplementary Figure 11

Supplementary Figure 12

Supplementary Figure Legends

Supplementary Table 1

Supplementary Table 2

Supplementary Table 3

Supplementary Table 4

Supplementary Table 5

Supplementary Table 6

Supplementary Table 7

Original Western Blots

aj-checklist

## Data Availability

All data generated or analyzed during this study are included in this article, and its supplementary information files. The RNA-sequencing data and RIP-sequencing data used in this paper have been deposited in the NCBI GEO database with accession code GSE193143.

## References

[1] Ling Z, Cheng B, Tao X (2021). Epithelial-to-mesenchymal transition in oral squamous cell carcinoma: challenges and opportunities. Int J Cancer.

[2] Zanoni DK, Montero PH, Migliacci JC, Shah JP, Wong RJ, Ganly I (2019). Survival outcomes after treatment of cancer of the oral cavity (1985–2015). Oral Oncol.

[3] Casar B, He Y, Iconomou M, Hooper JD, Quigley JP, Deryugina EI (2012). Blocking of CDCP1 cleavage in vivo prevents Akt-dependent survival and inhibits metastatic colonization through PARP1-mediated apoptosis of cancer cells. Oncogene.

[4] Li S, Wang N, Brodt P (2012). Metastatic cells can escape the proapoptotic effects of TNF-alpha through increased autocrine IL-6/STAT3 signaling. Cancer Res.

[5] Larkin J, Hodi FS, Wolchok JD (2015). Combined nivolumab and ipilimumab or monotherapy in untreated melanoma REPLY. N. Engl J Med.

[6] Yang F, Huo XS, Yuan SX, Zhang L, Zhou WP, Wang F (2013). Repression of the long noncoding RNA-LET by histone deacetylase 3 contributes to hypoxia-mediated metastasis. Mol Cell.

[7] Bakir B, Chiarella AM, Pitarresi JR, Rustgi AK (2020). EMT, MET, plasticity, and tumor metastasis. Trends Cell Biol.

[8] Kenny TC, Craig AJ, Villanueva A, Germain D (2019). Mitohormesis primes tumor invasion and metastasis. Cell Rep..

[9] Pascual G, Avgustinova A, Mejetta S, Martin M, Castellanos A, Attolini CS (2017). Targeting metastasis-initiating cells through the fatty acid receptor CD36. Nature.

[10] Pavlova NN, Thompson CB (2016). The emerging hallmarks of cancer metabolism. Cell Metab.

[11] Vyas S, Zaganjor E, Haigis MC (2016). Mitochondria and cancer. Cell.

[12] Xu WY, Hu QS, Qin Y, Zhang B, Liu WS, Ni QX (2018). Zinc finger E-box-binding homeobox 1 mediates aerobic glycolysis via suppression of sirtuin 3 in pancreatic cancer. World J Gastroenterol.

[13] Zhou Y, Lin F, Wan T, Chen A, Wang H, Jiang B (2021). ZEB1 enhances Warburg effect to facilitate tumorigenesis and metastasis of HCC by transcriptionally activating PFKM. Theranostics.

[14] Kim JY, Cho KH, Jeong BY, Park CG, Lee HY (2020). Zeb1 for RCP-induced oral cancer cell invasion and its suppression by resveratrol. Exp Mol Med.

[15] Guo W, Huai Q, Zhang G, Guo L, Song P, Xue X (2020). Elevated heterogeneous nuclear ribonucleoprotein C expression correlates with poor prognosis in patients with surgically resected lung adenocarcinoma. Front Oncol.

[16] Wang S, Zou X, Chen Y, Cho WC, Zhou X (2020). Effect of N6-methyladenosine regulators on progression and prognosis of triple-negative breast cancer. Front Genet.

[17] Zhang S, Wu X, Diao P, Wang C, Wang D, Li S (2020). Identification of a prognostic alternative splicing signature in oral squamous cell carcinoma. J Cell Physiol.

[18] Konig J, Zarnack K, Rot G, Curk T, Kayikci M, Zupan B (2010). iCLIP reveals the function of hnRNP particles in splicing at individual nucleotide resolution. Nat Struct Mol Biol.

[19] Velusamy T, Shetty P, Bhandary YP, Liu MC, Shetty S (2008). Posttranscriptional regulation of urokinase receptor expression by heterogeneous nuclear ribonuclear protein C. Biochemistry.

[20] Lee EK, Kim HH, Kuwano Y, Abdelmohsen K, Srikantan S, Subaran SS (2010). hnRNP C promotes APP translation by competing with FMRP for APP mRNA recruitment to P bodies. Nat Struct Mol Biol.

[21] Liu N, Dai Q, Zheng G, He C, Parisien M, Pan TN (2015). N6-methyladenosine-dependent RNA structural switches regulate RNA-protein interactions.. Nature.

[22] Herrmann C, Dybas JM, Liddle JC, Price AM, Hayer KE, Lauman R (2020). Adenovirus-mediated ubiquitination alters protein-RNA binding and aids viral RNA processing. Nat Microbiol.

[23] Ulitsky I, Bartel DP (2013). lincRNAs: genomics, evolution, and mechanisms. Cell.

[24] Engreitz JM, Haines JE, Perez EM, Munson G, Chen J, Kane M (2016). Local regulation of gene expression by lncRNA promoters, transcription and splicing. Nature.

[25] Tripathi V, Ellis JD, Shen Z, Song DY, Pan Q, Watt AT (2010). The nuclear-retained noncoding RNA MALAT1 regulates alternative splicing by modulating SR splicing factor phosphorylation. Mol Cell.

[26] Lee S, Kopp F, Chang TC, Sataluri A, Chen B, Sivakumar S (2016). Noncoding RNA NORAD regulates genomic Stability by sequestering PUMILIO proteins. Cell.

[27] Wu QN, Luo XJ, Liu J, Lu YX, Wang Y, Qi J (2021). MYC-Activated LncRNA MNX1-AS1 promotes the progression of colorectal cancer by stabilizing YB1. Cancer Res.

[28] Meng LD, Shi GD, Ge WL, Huang XM, Chen Q, Yuan H (2020). Linc01232 promotes the metastasis of pancreatic cancer by suppressing the ubiquitin-mediated degradation of HNRNPA2B1 and activating the A-Raf-induced MAPK/ERK signaling pathway. Cancer Lett.

[29] Tang J, Yan T, Bao Y, Shen C, Yu C, Zhu X (2019). LncRNA GLCC1 promotes colorectal carcinogenesis and glucose metabolism by stabilizing c-Myc. Nat Commun.

[30] Tang Z, Li C, Kang B, Gao G, Li C, Zhang Z (2017). GEPIA: a web server for cancer and normal gene expression profiling and interactive analyses. Nucleic Acids Res.

[31] Karinen S, Juurikka K, Hujanen R, Wahbi W, Hadler-Olsen E, Svineng G (2021). Tumour cells express functional lymphatic endothelium-specific hyaluronan receptor in vitro and in vivo: Lymphatic mimicry promotes oral oncogenesis?. Oncogenesis.

[32] Tian H, Shi S, You B, Zhang Q, Gu M, You Y (2021). ER resident protein 44 promotes malignant phenotype in nasopharyngeal carcinoma through the interaction with ATP citrate lyase. J Transl Med.

[33] Wu Y, Zhao W, Liu Y, Tan X, Li X, Zou Q, et al. Function of HNRNPC in breast cancer cells by controlling the dsRNA-induced interferon response. EMBO J. 2018;37:e99017. 10.15252/embj.201899017 PMC6276880 30158112

[34] Lang B, Armaos A, Tartaglia GG (2019). RNAct: Protein-RNA interaction predictions for model organisms with supporting experimental data. Nucleic Acids Res.

[35] Zhang Y, Chen W, Pan T, Wang H, Zhang Y, Li C (2019). LBX2-AS1 is activated by ZEB1 and promotes the development of esophageal squamous cell carcinoma by interacting with HNRNPC to enhance the stability of ZEB1 and ZEB2 mRNAs. Biochem Biophys Res Commun.

[36] Schiffmann LM, Werthenbach JP, Heintges-Kleinhofer F, Seeger JM, Fritsch M, Gunther SD (2020). Mitochondrial respiration controls neoangiogenesis during wound healing and tumour growth. Nat Commun.

[37] Liu Y, Gong CS, Lin L, Zhou Z, Liu Y, Yang Z (2019). Core-shell metal-organic frameworks with fluorescence switch to trigger an enhanced photodynamic therapy. Theranostics.

[38] Liu Y, Hou W, Xia L, Cui C, Wan S, Jiang Y (2018). ZrMOF nanoparticles as quenchers to conjugate DNA aptamers for target-induced bioimaging and photodynamic therapy. Chem Sci.

[39] Ganapathy-Kanniappan S, Geschwind JF (2013). Tumor glycolysis as a target for cancer therapy: progress and prospects. Mol Cancer.

[40] Zhang Y, Yang JM (2013). Altered energy metabolism in cancer: a unique opportunity for therapeutic intervention. Cancer Biol Ther.

[41] Zhao J, Huang X, Xu Z, Dai J, He H, Zhu Y (2017). LDHA promotes tumor metastasis by facilitating epithelialmesenchymal transition in renal cell carcinoma. Mol Med Rep..

[42] Viale A, Pettazzoni P, Lyssiotis CA, Ying H, Sanchez N, Marchesini M (2014). Oncogene ablation-resistant pancreatic cancer cells depend on mitochondrial function. Nature.

[43] Schieber M, Chandel NS (2014). ROS function in redox signaling and oxidative stress. Curr Biol.

[44] Steeg PS (2016). Targeting metastasis. Nat Rev Cancer.

[45] Cirenajwis H, Lauss M, Ekedahl H, Torngren T, Kvist A, Saal LH (2017). NF1-mutated melanoma tumors harbor distinct clinical and biological characteristics. Mol Oncol.

[46] Kalyane D, Raval N, Maheshwari R, Tambe V, Kalia K, Tekade RK (2019). Employment of enhanced permeability and retention effect (EPR): Nanoparticle-based precision tools for targeting of therapeutic and diagnostic agent in cancer. Mater Sci Eng C Mater Biol Appl.

[47] Caramel J, Ligier M, Puisieux A (2018). Pleiotropic roles for ZEB1 in cancer. Cancer Res.

[48] Aigner K, Dampier B, Descovich L, Mikula M, Sultan A, Schreiber M (2007). The transcription factor ZEB1 (deltaEF1) promotes tumour cell dedifferentiation by repressing master regulators of epithelial polarity. Oncogene.

[49] Zarnack K, Konig J, Tajnik M, Martincorena I, Eustermann S, Stevant I (2013). Direct competition between hnRNP C and U2AF65 protects the transcriptome from the exonization of Alu elements. Cell.

