# Supplementary material for: lncRNA CYTOR promotes aberrant glycolysis and mitochondrial respiration via HNRNPC-mediated ZEB1 stabilization in oral squamous cell carcinoma
Source: Cell Death Dis. 2022 Aug 13;13(8):703. doi: 10.1038/s41419-022-05157-1 (PMC9376070; doi:10.1038/s41419-022-05157-1)
Supplement: Supplementary file 1 — Supplementary Materials [file 41419_2022_5157_MOESM1_ESM.docx]

**lncRNA CYTOR promotes aberrant glycolysis and mitochondrial respiration via HNRNPC-mediated ZEB1 stabilization in oral squamous cell carcinoma**

**・Authors’ names:**

Weiwen Zhu^1,2,3^, Jie Wang^1,2,3^, Xiang Liu^1,2,3^, Yanbin Xu^1,2,3^, Rundong Zhai^1,2,3^, Jiayi Zhang^1,2,3^, Mengqi Wang^1,2,3^, Mengyao Wang^1,2,3^, Laikui Liu^1,2,3*^

**・Authors’ affiliations:**

^1^Department of Basic Science of Stomatology, The Affiliated Stomatological Hospital of Nanjing Medical University, Jiangsu, China;

^2^Jiangsu Province Key Laboratory of Oral Diseases, Nanjing Medical University, Jiangsu, China;

^3^Jiangsu Province Engineering Research Center of Stomatological Translational Medicine, Jiangsu, China;

*** Corresponding author:**

Dr Laikui Liu, Department of Basic Science of Stomatology, Affiliated Hospital of Stomatology, Nanjing Medical University. 136# Hanzhong Road, Nanjing, Jiangsu 210029, China. E-mail: [my_yunkong@njmu.edu.cn](mailto:my_yunkong@njmu.edu.cn)

**Supplementary Data**

**Primers and antibodies**

The primers and antibodies are listed in the following table (Characterization of the sequences of siRNA, shRNA, RT-qPCR primers, chip primers and antibody).

**Sequences for siRNAs or shRNA**

shCYTOR-#1 GGCUUGAACAUUUGGUCUUTT

shCYTOR-#2 CUCUACUCAUGCCCAAAGUTT

shCYTOR-#3 GAAACCUCUUGACUCUUCUTT

shZEB1 GAACCAGTTGTAAATGTAA

siHNRNPC-#1 CGTCAGCGTGTATCAGGAA

siHNRNPC-#2 AGGTGTGAAACGATCTGCA

siHNRNPC-#3 GTGAAGAAAGATGAGACTA

**Primers for qPCR**

GAPDH-F GAACGGGAAGCTCACTGG

GAPDH-R GCCTGCTTCACCACCTTCT

CYTOR-F ATCCACATTCCAACCTCCGT

CYTOR-R CTGAGTCGTGATTTTCGGTGT

HOTAIRM1-F AAACTCCGTGTTACTCATTCCTG

HOTAIRM1-R GAATTGGACAGTCTAAGATTTGGG

HNRNPC-F GGCTTTGTGAGAAACCTTACCAT

HNRNPC-R TTCATGGAGCGAGGATCTGTC

COX10-F TCCTCAAACGCATGTATGTCAC

COX10-R CTGGTTCTGGCTTGGGTCTT

SIRT-F TCCTTCCTAGCATCACATTACCTG

SIRT3-R CACTGTCATTAAATCTATTGCCGTC

ZEB1-F ACACGACCACAGATACGGCA

ZEB1-R GAAGCAACCACTATGGGTTTGA

**Primers for chIP**

SIRT3-1-F GCCGGAAATGCTCACTCAC

SIRT3-1-R GAGTTTTGTCCGGAGCGCAG

SIRT3-2-F TGTTTCCCAAAGTGGGTGGT

SIRT3-2-R TGAACTCCTACCTCGGGTCT

SIRT3-3-F GCAGAAGGCTGCTGAATGCTA

SIRT3-3-R TCTCCTGTAGTAACCTCCCTGT

SIRT3-4-F CAATGCATTTCTGATAAGGGATACT

SIRT3-4-R TAGCATTCTTTCTGCTACACCGT

COX10-1-F GCTTTTTCTTCAGGACAGCGG

COX10-1-R GAGGTGATCGGAAGGGACTG

COX10-2-F GGATTTCACTCCAGACCACCA

COX10-2-R ATCAAATGGGAGCCCAGAGG

COX10-3-F CGACAACCTCTTGTCCCCTC

COX10-3-R AGCCGTTTTGGTTCACCTCA

COX10-4-F CAGGTCTTGGTTCTGGCACT

COX10-4-R AAGTCTGGCACATTAAGGCCC

**Antibodies for western blot**

Vimentin CST (5741#) 1:1000

Snail CST (3879#) 1:1000

E-Cadherin CST (14472#) 1:1000

N-Cadherin CST (13116#) 1:1000

β-Actin CST (3700#) 1:1000

SIRT3 Proteintech (1099-1-AP) 1:1000

CTP(SLC25A1)  Proteintech (15235-1-AP) 1:1000

SOD1  Proteintech (10269-1-AP) 1:1000

SOD2 Proteintech (24127-1-AP) 1:1000

HNRNPC  Proteintech (11760-1-AP) 1:1000

FLAG Sigma (F7425) 1:1000

Tublin CST (2148) 1:1000

GAPDH CST (5174) 1:1000

ZEB1 Proteintech (21544-1-AP) 1:1000

Citrate Sunthase (CS) CST (14309S) 1:1000

COX10 Proteintech (10611-2-AP) 1:1000

**Supplementary materials and methods**

**Real-time quantitative PCR (qPCR) analysis**

RNA was extracted from cells using Trizol reagent and reverse transcribed using the PrimeScript RT Reagent Kit (Takara Bio, Kusatsu, Japan). Real-time quantitative PCR analyses were per- formed in triplicate using the SYBR Green PCR Master Mix (Takara Bio) and detected using an Applied Biosystems 7900 Real-Time PCR System (Thermo Fisher Scientific, Waltham, MA, USA). The primer sequences used in this study are listed in Supplemental data. The values were normalized to the endogenous control, and the 2^-ΔΔCt^ method was used to calculate the relative quantification of gene expression.

**Western blot analysis**

Western blot was performed using methods previously described (1). Briefly, cell lysates were separated on polyacrylamide–sodium dodecyl sulfate gel and electroblotted onto nitrocellulose membranes (Bio-Rad, Hercules, CA, USA). After blocking with 5% nonfat dry milk, the membranes were incubated with various antibodies overnight. The information about primary antibodies is provided in Supplemental data. Then, membranes were incubated with a horseradish peroxidase–conjugated secondary antibody for 50 mins. The signals were visualized using ECL detection (Thermo Fisher Scientific).

**Wound-healing and invasion assays**

Cells were plated in six-well plates and grown to 90% confluence. A pipette tip was used to scratch wounds, and then cells were incubated with basial medium. Migrating cells at the wound front were photographed at 0 and 12 hours. Cell invasion assays were carried out by using 8-μm pore Transwell filters (Costar, Lowell, MA, USA) that were precoated with Matrigel (Corning, Bedford, MA, USA). Cells (2.0 × 10^5^) were resuspended in 200 μL serum-free medium and added to the upper chamber, while the lower chamber was filled with complete medium as the chemoattractant. After incubation for 24 hours, the migrated cells were fixed with 4% PFA and stained with crystal violet (Sigma-Aldrich, St Louis, MO USA). Migratory cells on the lower surface of the chamber were counted and photographed (Olympus, Tokyo, Japan).

**Cell lines**

HNSCC cell lines Cal27 and HN6 were used. Cal27 and HN6 cell lines were purchased from the American Type Culture Collection (ATCC, Manassas, VA, USA). Cell lines were authenticated using human cancer cell line STR profiles. All cancerous cell lines were maintained in Dulbecco’s modified Eagle’s medium (DMEM) supplemented with 10% fetal bovine serum (Gibco) and 1% penicillin/ streptomycin at 37 °C in a 5% CO^2^-humidified incubator. All cells were routinely tested for mycoplasma at regular intervals throughout the whole course of the study.

**Patient-derived cells (PDCs)**

The OSCC patient samples were obtained from surgeries (Nanjing Medical University affiliated Stomatological Hospital, China). Tumors were collected into the solution contains DMEM/F12/HEPES (GIBCO), 1mg/ml Collagenase (Roche), 100U/ml Hyaluronidase (Sigma), 25% BSA fraction V (GIBCO), 5 mg/ml Insulin and 50 mg/ml Gentamycin (GIBCO). Samples were first triturated with a pipette to break down tissues clump, then incubated at 37°C for 30 mins with rotation. The harvested cell suspensions were then filtered through 100 μm nylon mesh filter, then centrifuged for 5 min at 250 × g. After centrifugation the cells were suspended into the complete growth medium.

**RNA immunoprecipitation (RIP)**

RIP assays were performed with a Magna RNA-binding protein immunoprecipitation kit (Millipore, #17-704), according to the manufacturer’s instructions. 1 × 10^7^ cells were subsequently washed twice with 10ml ice-cold PBS, and the pellets were collected and resuspended in 115 μL RIP lysis buffer on ice for 10 mins before use. Negative control IgG, human anti-HNRNPC antibody (5 μg/sample, Proteintech Cat# 11760-1-AP, RRID:AB_2117500) and anti-FLAG tag antibody (5 μg/sample, Sigma-Aldrich Cat# F9291, RRID:AB_439698) were used in this study. After proteinase K digestion, the immunoprecipitated RNAs were extracted, purified, and subjected to qPCR.

**RNA pull-down assay**

Biotin-labeled full-length or truncated fragments of CYTOR and ZEB1 RNA were transcribed in vitro with a Pierce RNA 3’ End Desthiobiotinylation Kit (Thermo Scientific, #20163) and T4 RNA ligase (InvitrogenTM, MEGAsciptTM, #AM1330) using PCR products as a template. Cells were washed twice with 10 ml ice-cold PBS and resuspended by 100μl Pierce IP lysis buffer (Thermo Scientific, #87787). RNA pull-down assays were performed with a Pierce Magnetic RNA–Protein Pull-Down Kit (Thermo Scientific, #20164). According to the manufacturer’s instructions, biotinylated RNA was captured with streptavidin magnetic beads and then incubated with the 30μl cell lysates at 4 °C for 2h rotation before washing and elution of the RBP complex. The eluted proteins were subjected to western blot.

**Chromatin Immunoprecipitation (ChIP)**

Chromatin immunoprecipitation (ChIP) analysis was carried out using EZ-ChIP kit (Millipore,17-10086) according to the manufacturer’s protocol. Briefly, HN6 and Cal27 cells were crosslinked with fresh 4% formaldehyde; subsequently, the cells were lysed in SDS buffer and sonicated to shear the DNA into 200-1000bp. Lysates diluted with ChIP dilution buffer were immunoprecipitated with anti-ZEB1 antibody (Proteintech Cat# 21544-1-AP, RRID:AB_10734325) and rabbit IgG was used as an internal control. Reverse-crosslinked DNA was transferred to real-time PCR analysis. Detailed information, including the primer sequences used for real-time PCR, is listed in Supplemental data.

**Oxygen consumption rate (OCR) and extracellular acidification rate (ECAR) assay**

To assess the impact of CYTOR and ZEB1 on mitochondrial respiration and glycolytic capacity of oral cancer cells, Seahorse Bioscience XFe96 (Agilent Seahorse XFe96 Analyzer, RRID:SCR_019545) Extracellular Flux Analyzer was used according to the manufacturer’s instructions. The mitochondrial respiration and glycolytic capacity were determined using the Mito-stress Test Kit and Glycolysis Stress Test Kit as the manufacturer's instructions. Briefly, 1 × 10^4^ HN6 (RRID : CVCL_8129, obtained from ATCC) cells and 2 × 10^4^ Cal27 (ATCC Cat# CRL-2095, RRID : CVCL_1107, obtained from ATCC) cells were seeded onto 96-well plates and incubated overnight. After washing the cells with Seahorse detection buffer, 175 μL of Seahorse detection buffer plus 25 μL each of 1 μmol/L oligomycin, 1 μmol/L FCCP, and 1 μmol/L rotenone was automatically injected to measure the oxygen consumption rate (OCR). Then, 25 μL each of 10 mmol/L glucose, 1 μmol/L oligomycin, and 100 mmol/L 2-deoxy-glucose were added to measure the extracellular acidification rate (ECAR). The OCR and ECAR values were calculated after normalization to the cell number and are plotted as the mean ± SD.

**Dual Luciferase Reporter Assay**

The putative binding regions of ZEB1 in the human SIRT3 and COX10 promoter were amplified by PCR from genomic DNA and cloned downstream of the firefly luciferase gene (FL) in the pGL3-basic luciferase reporter vector (Genecopoeia, Guangzhou, Guangdong, China). For luciferase reporter assays, 293T cells were co-transfected with individual pGL3-SIRT3 and pGL3-COX10 reporter plasmids and LeV-ZEB1 plasmids using lipofectamine 2000. At 48 hours post-transfection, cells lysates were collected and assayed with a Dual-Luciferase Assay kit (Promega, Madison, WI, USA) following the manufacturer’s instructions. The pRL Renilla luciferase (RL) reporter was used for data normalization. Results are displayed as the ratio of FL/RL activity.

**cDNA Library Construction and Sequencing**

Sequence libraries were generated and sequenced by CapitalBio Technology (Beijing, China). The triplicate samples of all assays were constructed an independent library, and do the following sequencing and analysis. The NEB Next Ultra RNA Library Prep Kit for Illumina (NEB) was used to construct the libraries for sequencing. NEB Next Poly(A) mRNA Magnetic Isolation Module (NEB) kit was used to enrich the poly(A) tailed mRNA molecules from 1 μg total RNA. The mRNA was fragmented into ∼200 base pair pieces. The first-strand cDNA was synthesized from the mRNA fragments reverse transcriptase and random hexamer primers, and then the second-strand cDNA was synthesized using DNA polymerase I and RNaseH. The end of the cDNA fragment was subjected to an end repair process that included the addition of a single “A” base, followed by ligation of the adapters. Products were purified and enriched by polymerase chain reaction (PCR) to amplify the library DNA. The final libraries were quantified an Agilent 2100 Bioanalyzer. After quantitative reverse transcription-polymerase chain reaction (RT-qPCR) validation, libraries were subjected to paired-end sequencing with pair end 150-base pair reading length on an Illumina HiSeq sequencer (Illumina).

**Mass Spectrometry analyses**

Experiments were performed on a Q Exactive mass spectrometer that was coupled to Easy nLC (Thermo Fisher Scientific). The peptide mixture was loaded onto a the C18-reversed phase column (15 cm long, 75 μm inner diameter) packed in-house with RP-C18 5μm resin in buffer A (0.1% Formic acid in HPLC-grade water) and separated with a linear gradient of buffer B (0.1% Formic acid in 84% acetonitrile ) at a flow rate of 250 nl/min controlled by IntelliFlow technology over 60 min. MS data was acquired using a data-dependent top10 method dynamically choosing the most abundant precursor ions from the survey scan (300–1800 m/z) for HCD fragmentation. Determination of the target value is based on predictive Automatic Gain Control (pAGC). Dynamic exclusion duration was 20 s. Survey scans were acquired at a resolution of 70,000 at m/z 200 and resolution for HCD spectra was set to 17,500 at m/z 200. Normalized collision energy was 27 eV and the underfill ratio, which specifies the minimum percentage of the target value likely to be reached at maximum fill time, was defined as 0.1%.

**Immunohistochemistry (IHC) staining**

Paraffin-embedded OSCC tumor tissue sections (4 μm) were treated with xylene for deparaffinization, rehydrated with an ethanol gradient, treated for 20 min with 3% hydrogen peroxide. Following antigen retrieval, samples were blocked with normal goat serum and then probed overnight with primary antibodies. An HRP-polymer anti-rabbit/mouse Kit and a DAB Detection Kit (Fuzhou Maixin Biotech, Fuzhou, China) were then used to stain samples, and hematoxylin was employed for counterstaining. An ethanol gradient was then used to dehydrate samples, which were clarified with xylene and mounted using neutral gum.

**Pathological staining analyses**

Two pathologists independently analyzed pathological samples. The staining of indicated antibodies or FISH images were assessed by immunoreactivity score (IRS) values calculated as: IRS = IS × PS. IS scoring was as follow: 0, negative; 1, weak; 2, moderate; 3, strong. PS scoring was as follows: 0, negative; 1, <10%; 2, 11%-50%; 3, 51%-80%; 4, >80% positive staining. Patients were then separated into low- and high-expression subgroups based on IRS scores of 0-4 and >4, respectively.

**The synthesis of UiO-66-NH2 Nanoscale Metal Organic Frameworks (NMOFs)**

Total 80mg of ZrCl4 was added to 20 mL N,N-Dimethylformamide (DMF). Under vigorous stirring, 62 mg NH2-BDC and 25 μL were added. Then, the mix solution was kept in an oven at 120 ℃ under static conditions. After 24 h, the solutions were cooled to room temperature and the precipitates were isolated by centrifugation. Finally, the solids were dried under reduced pressure.

**The synthesis of siRNA@UiO-66-NH2 NMOFs**

8 mg UiO-66-NH2 dispersed in 8 mL DEPC-treated water was mixed with 2 mL siRNA solution (5 nM), and the mixture was stirred (800rpm) at room temperature for 2 h to afford siRNA@UiO-66-NH2. After siRNA loading, the mixture was centrifuged at 12000 rpm for 15 mins. The supernatant was discarded to remove the free siRNA and the precipitate was collected by freeze-drying.

**Reference**

1. Zhu W, Xu R, Du J, Fu Y, Li S, Zhang P, et al. Zoledronic acid promotes TLR-4-mediated M1 macrophage polarization in bisphosphonate-related osteonecrosis of the jaw. FASEB J. 2019;33(4):5208-19.

**Supplementary Tables**

**Table S1.** **Differentially expressed genes of CYTOR-KD and NC cells identified in the RNA-seq analysis.**

**Table S2. List of the possible CYTOR-interacting proteins identified by mass spectrometry.**

**Table S3.** **The ubiquitinated lysine residues in HNRNPC identified by mass spectrometry.**

**Table S4. Differentially expressed genes of HNRNPC-KD and NC cells identified in the RNA-seq analysis.**

**Table S5. The set of enriched protein coding genes identified by HNRNPC RIP-seq data.**

**Table S6. The list of HNRNPC-interacting genes with identified ENCODE eCLIP data.**

**Table S7. Associations between clinical variables and expression levels of HNRNPC and ZEB1 in OSCC.**
