# Supplementary figures and images for: lncRNA CYTOR promotes aberrant glycolysis and mitochondrial respiration via HNRNPC-mediated ZEB1 stabilization in oral squamous cell carcinoma
Source: Cell Death Dis. 2022 Aug 13;13(8):703. doi: 10.1038/s41419-022-05157-1 (PMC9376070; doi:10.1038/s41419-022-05157-1)

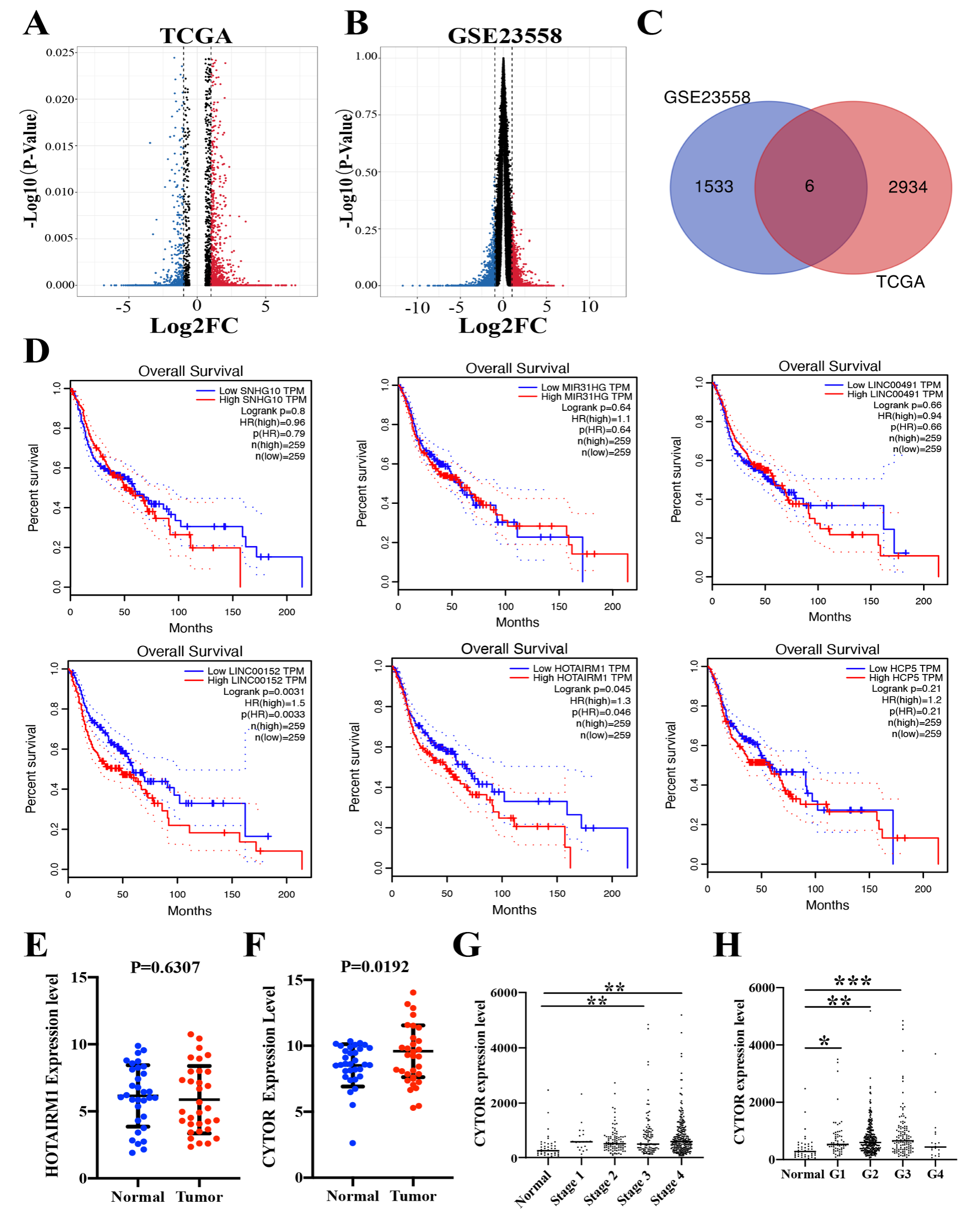

Supplement: Supplementary file 2 — Supplementary Figure 1 [file 41419_2022_5157_MOESM2_ESM.png]

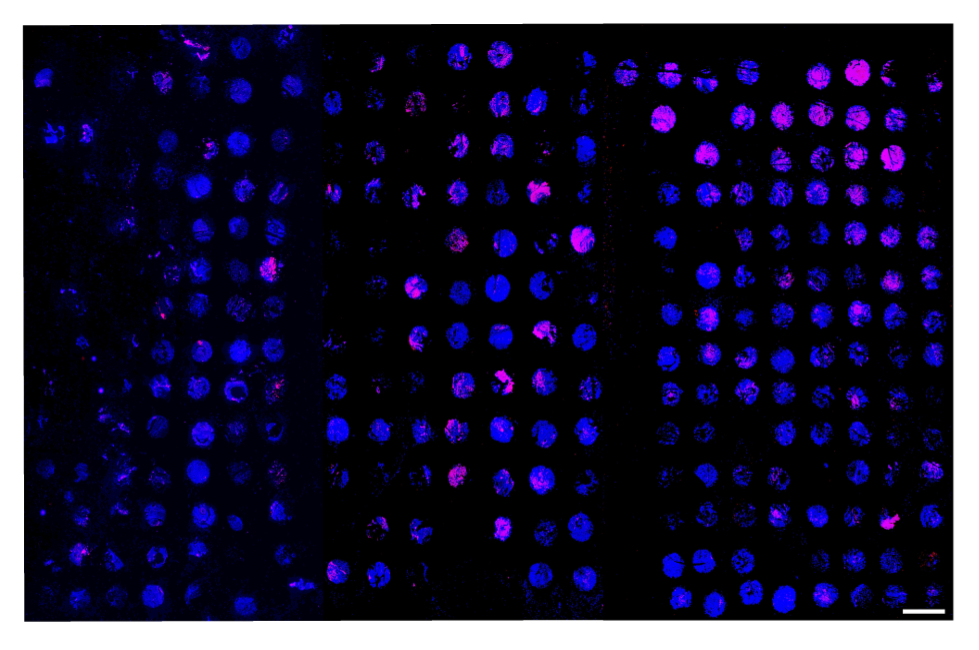

Supplement: Supplementary file 3 — Supplementary Figure 2 [file 41419_2022_5157_MOESM3_ESM.png]

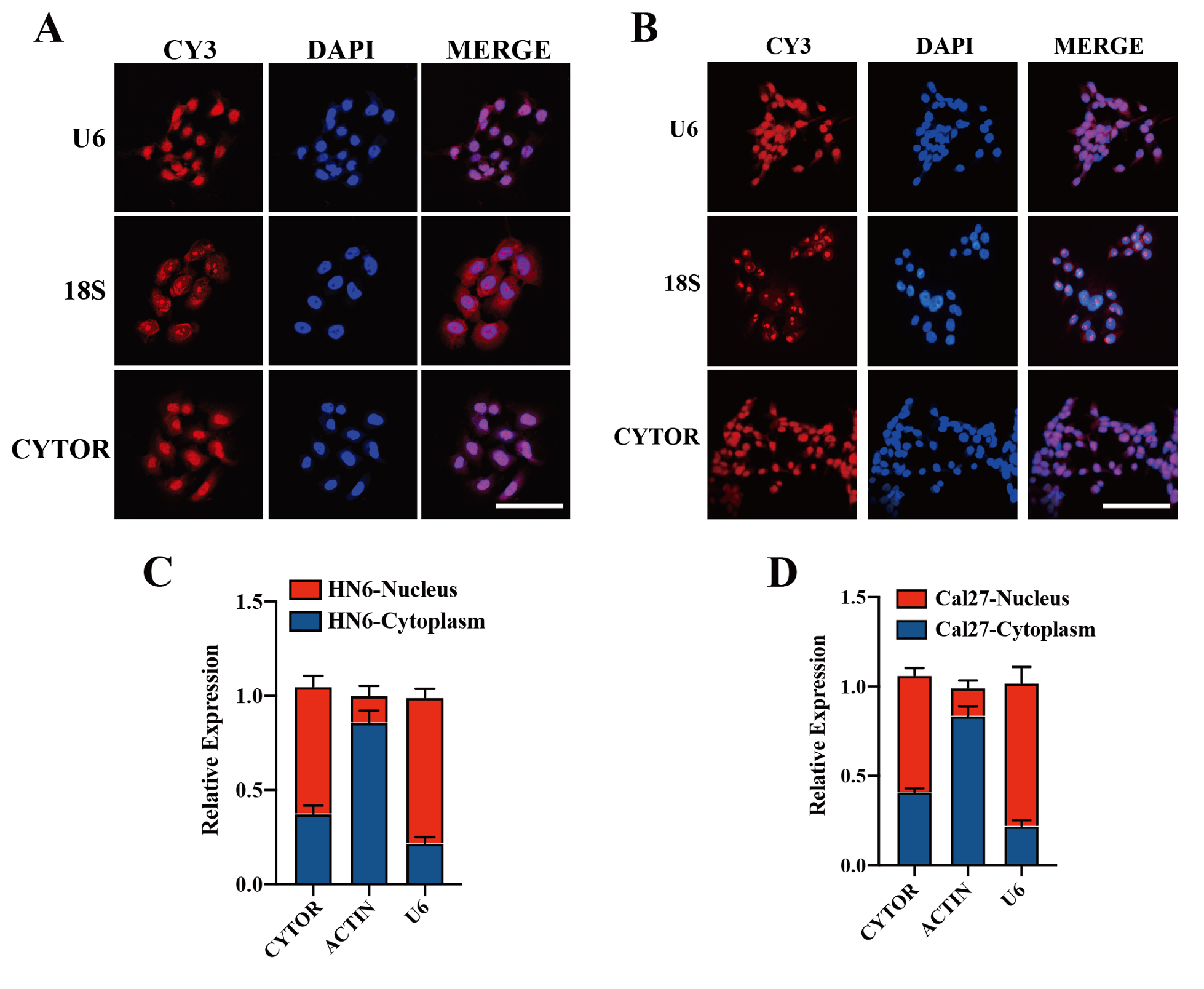

Supplement: Supplementary file 4 — Supplementary Figure 3 [file 41419_2022_5157_MOESM4_ESM.png]

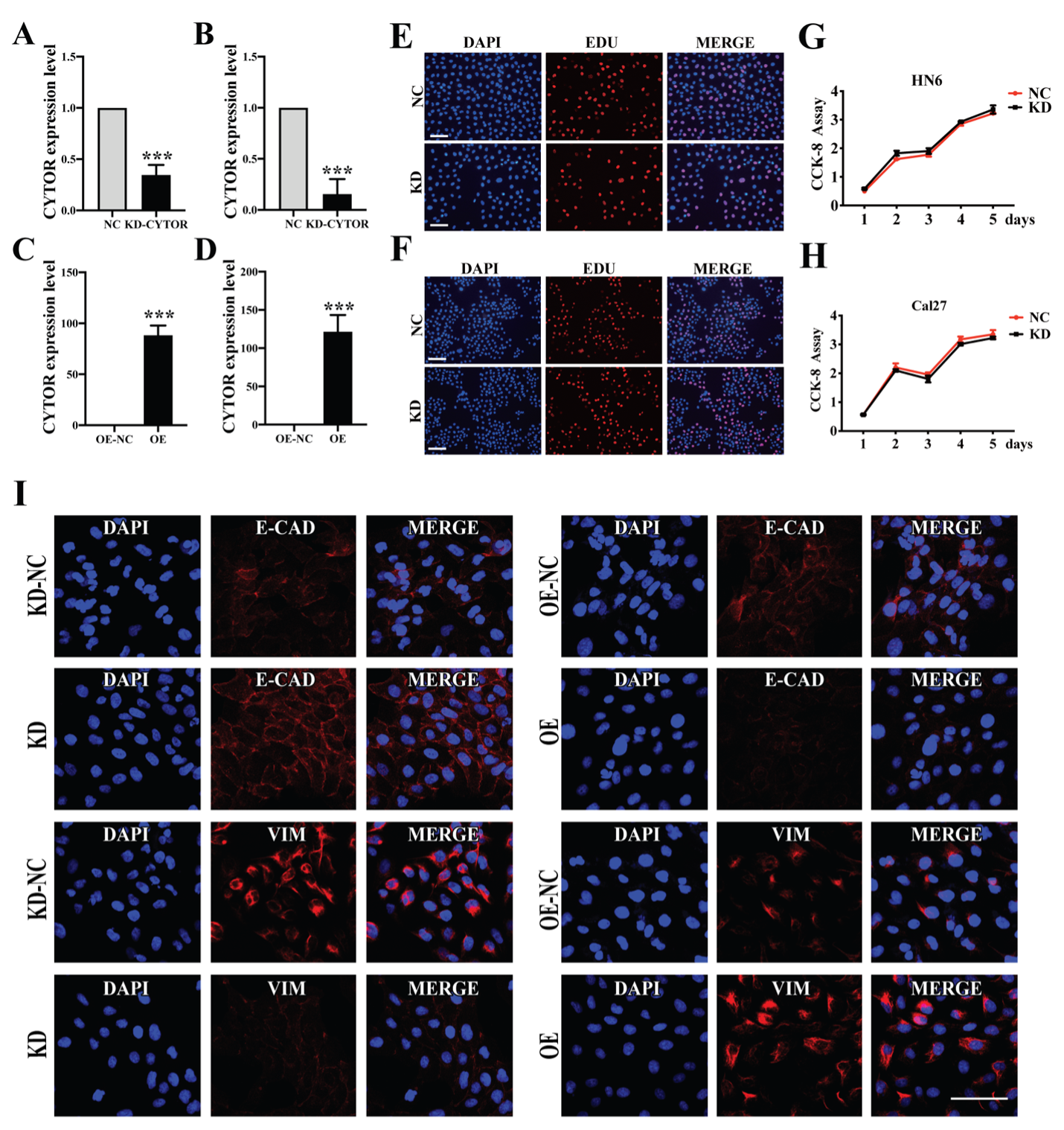

Supplement: Supplementary file 5 — Supplementary Figure 4 [file 41419_2022_5157_MOESM5_ESM.png]

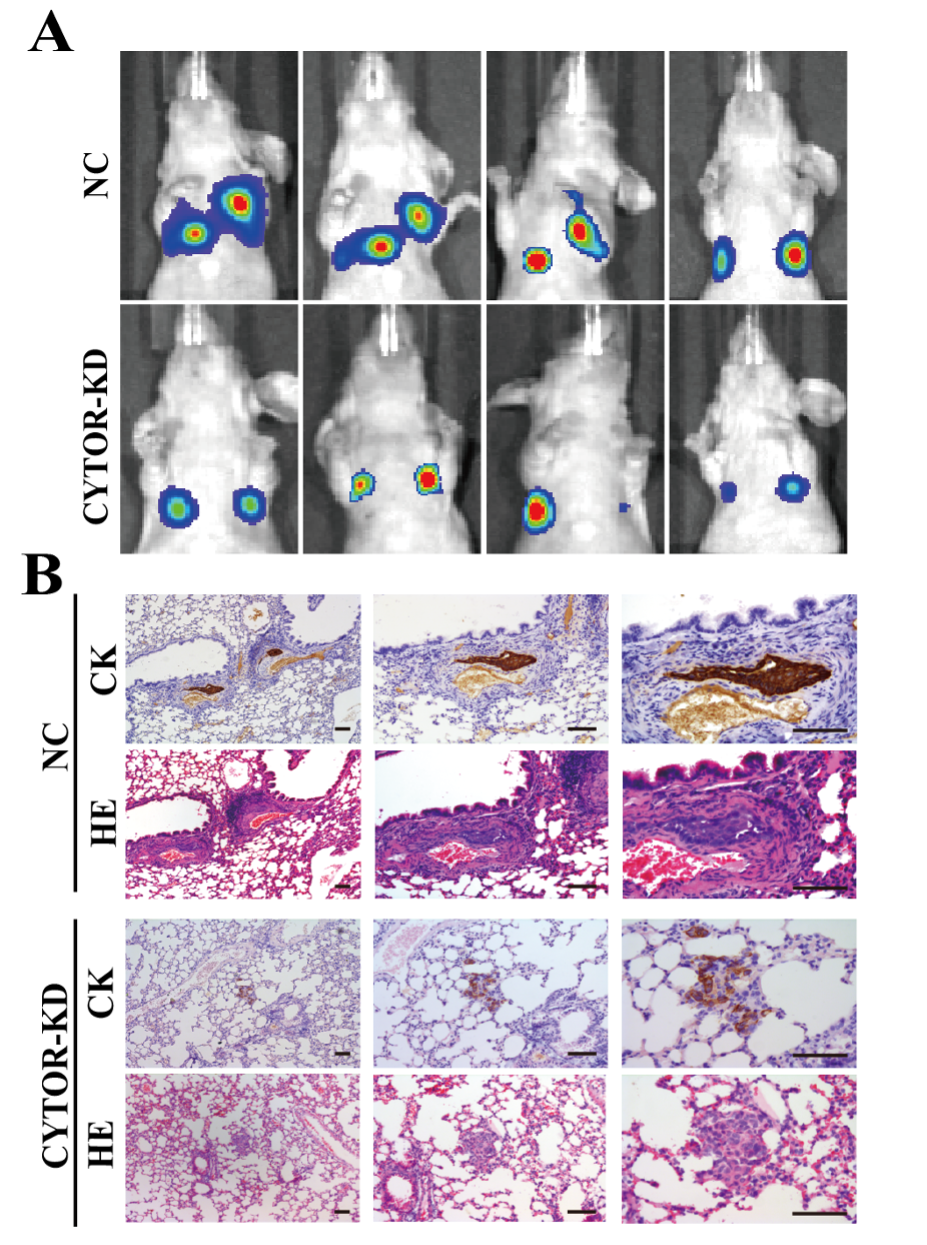

Supplement: Supplementary file 6 — Supplementary Figure 5 [file 41419_2022_5157_MOESM6_ESM.png]

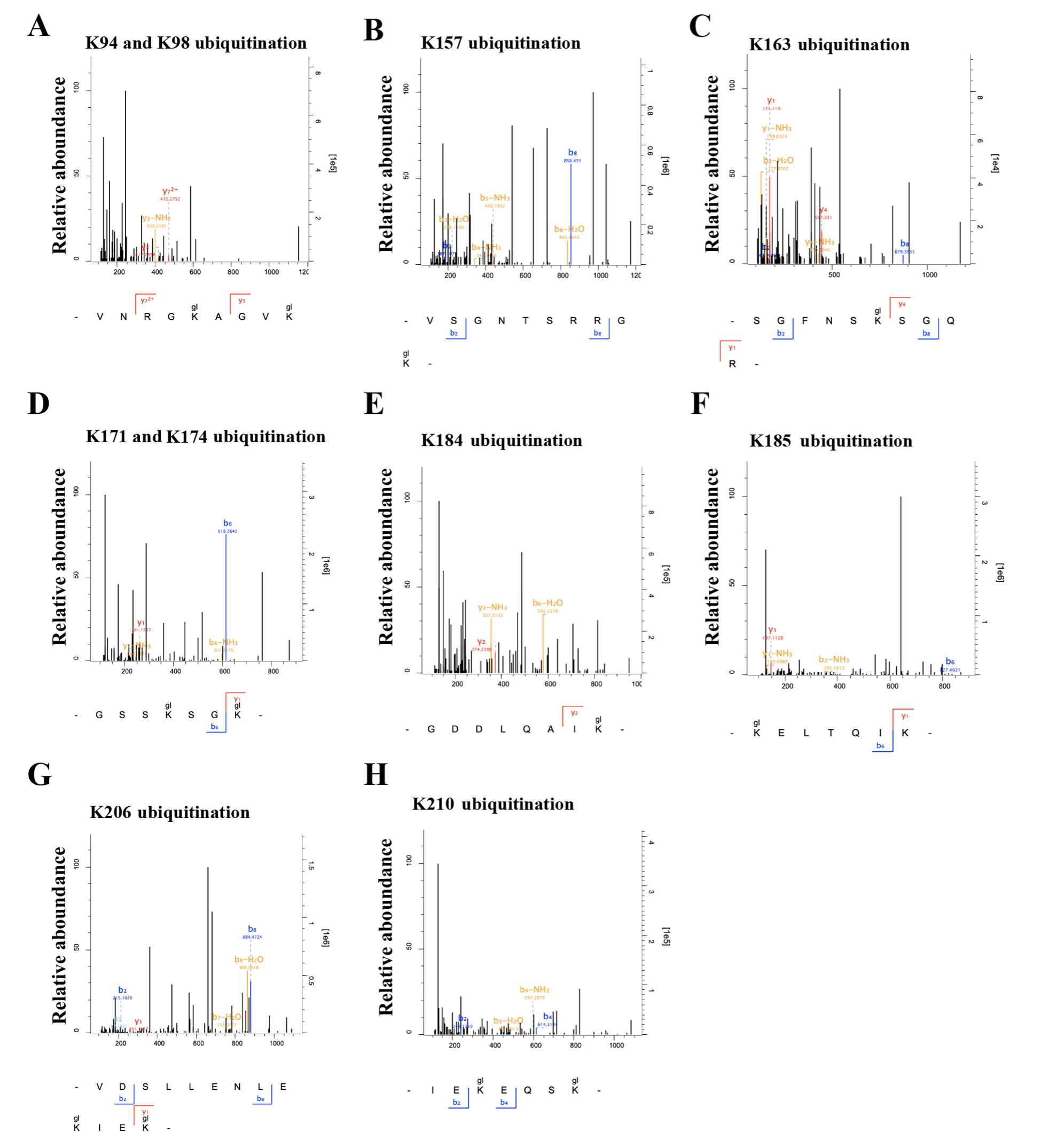

Supplement: Supplementary file 7 — Supplementary Figure 6 [file 41419_2022_5157_MOESM7_ESM.png]

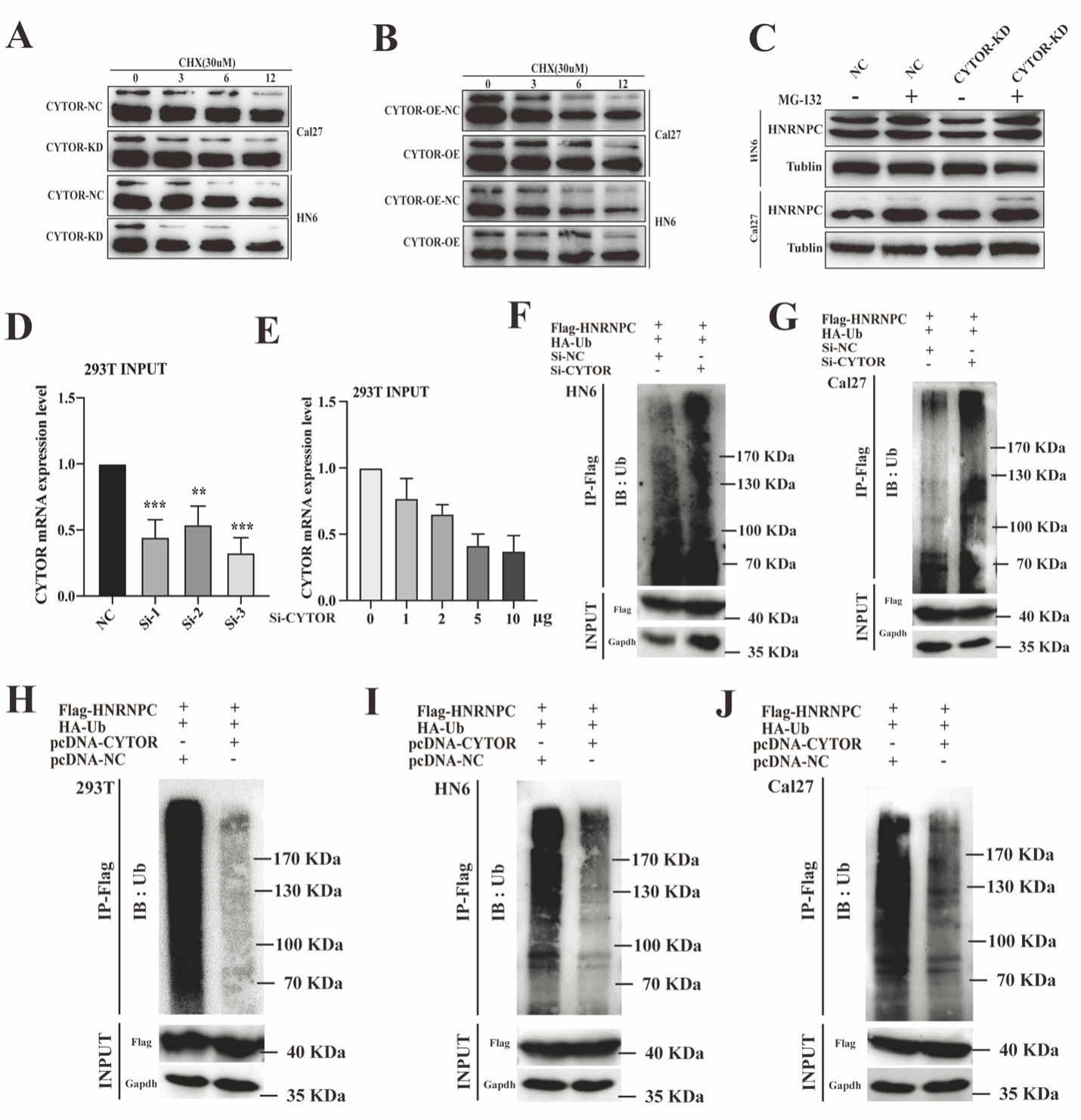

Supplement: Supplementary file 8 — Supplementary Figure 7 [file 41419_2022_5157_MOESM8_ESM.png]

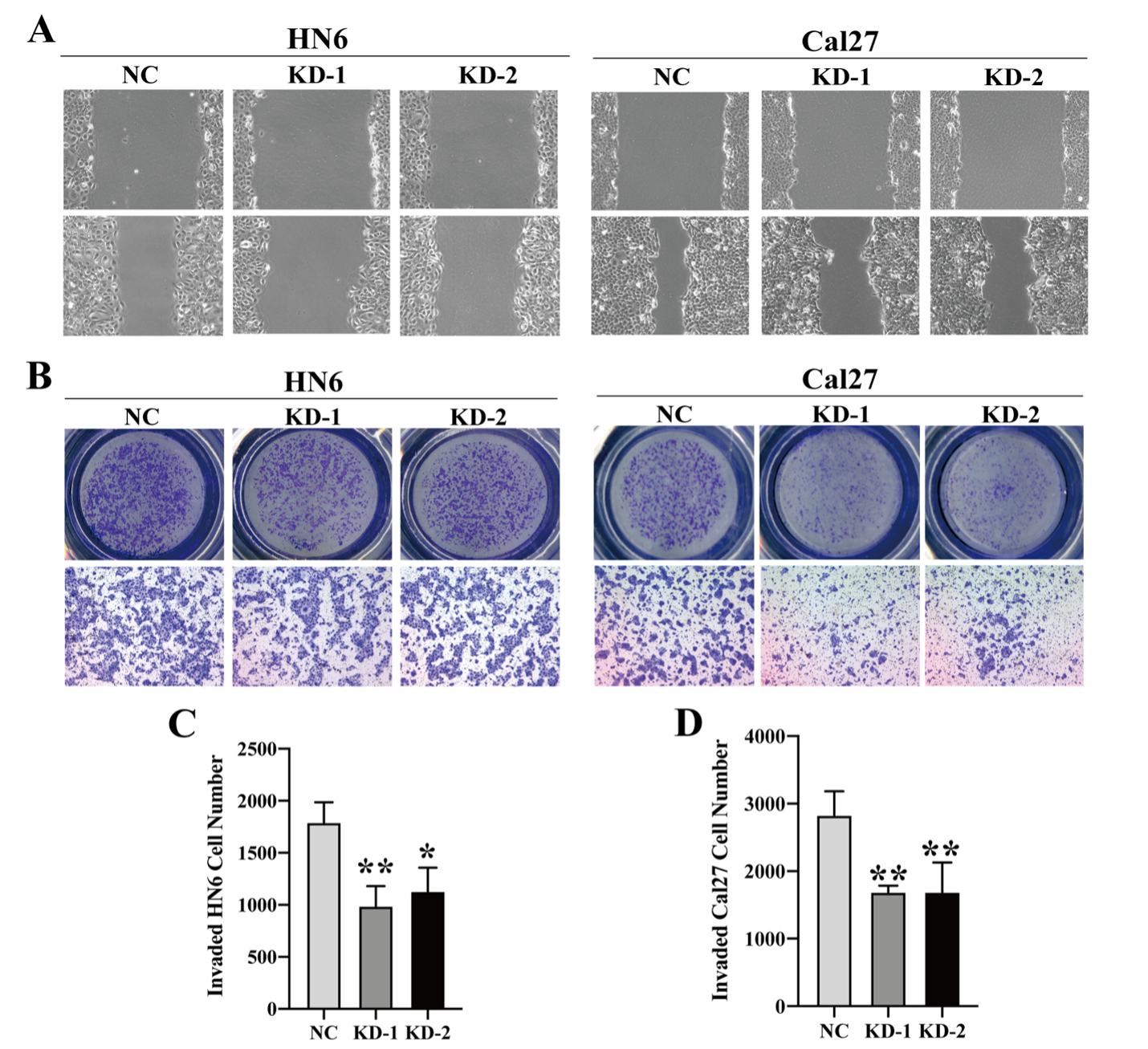

Supplement: Supplementary file 9 — Supplementary Figure 8 [file 41419_2022_5157_MOESM9_ESM.png]

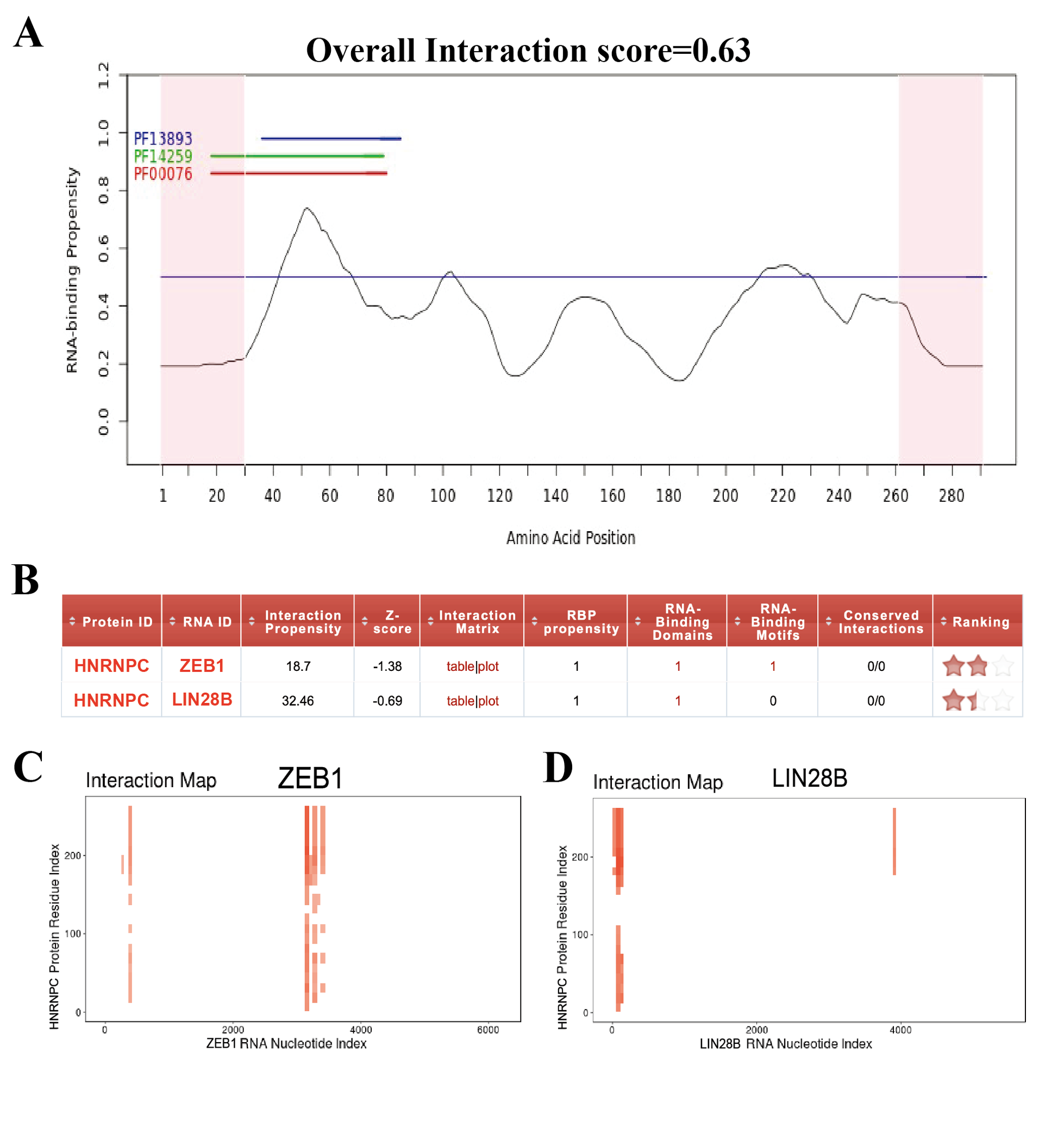

Supplement: Supplementary file 10 — Supplementary Figure 9 [file 41419_2022_5157_MOESM10_ESM.png]

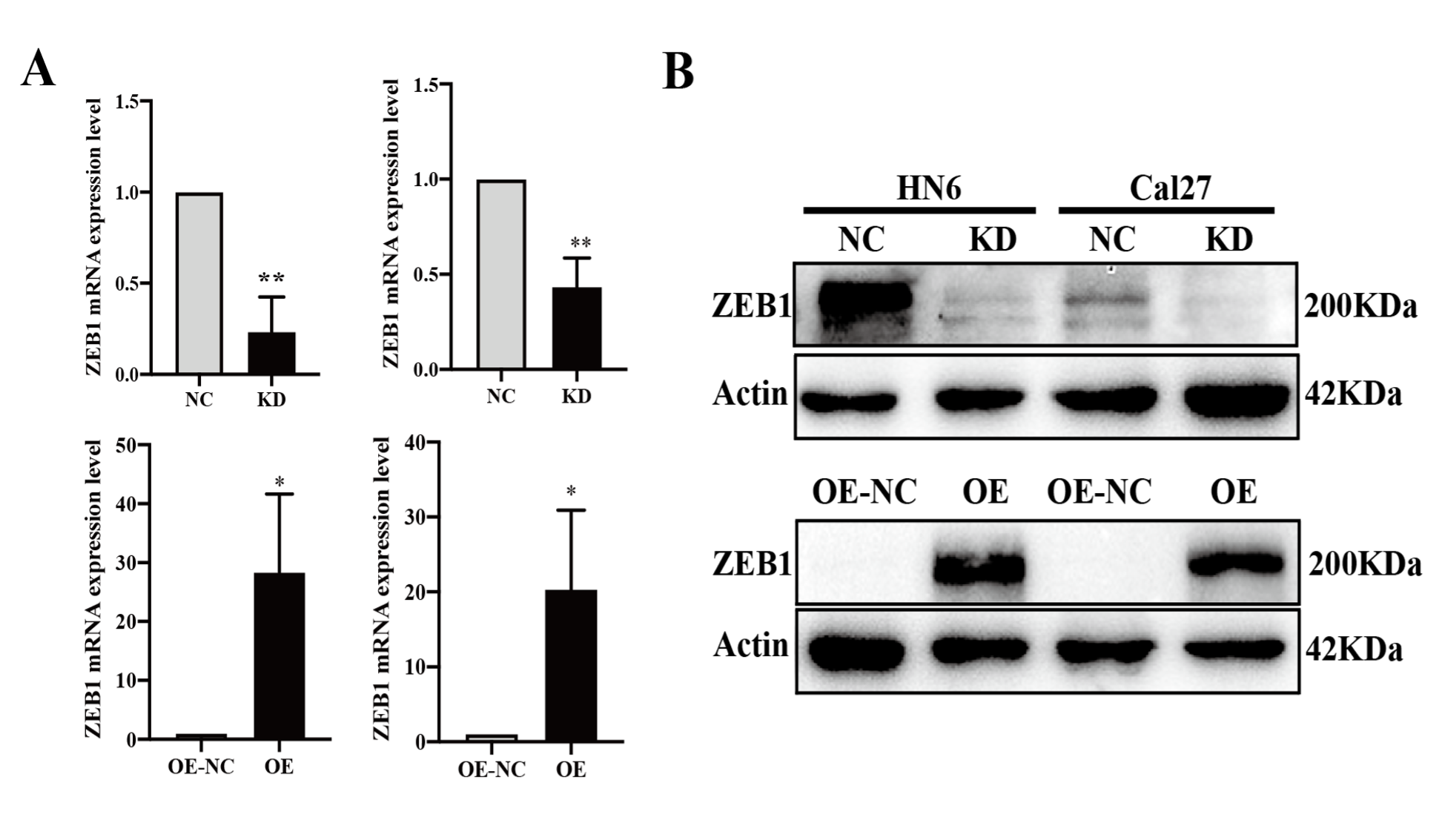

Supplement: Supplementary file 11 — Supplementary Figure 10 [file 41419_2022_5157_MOESM11_ESM.png]

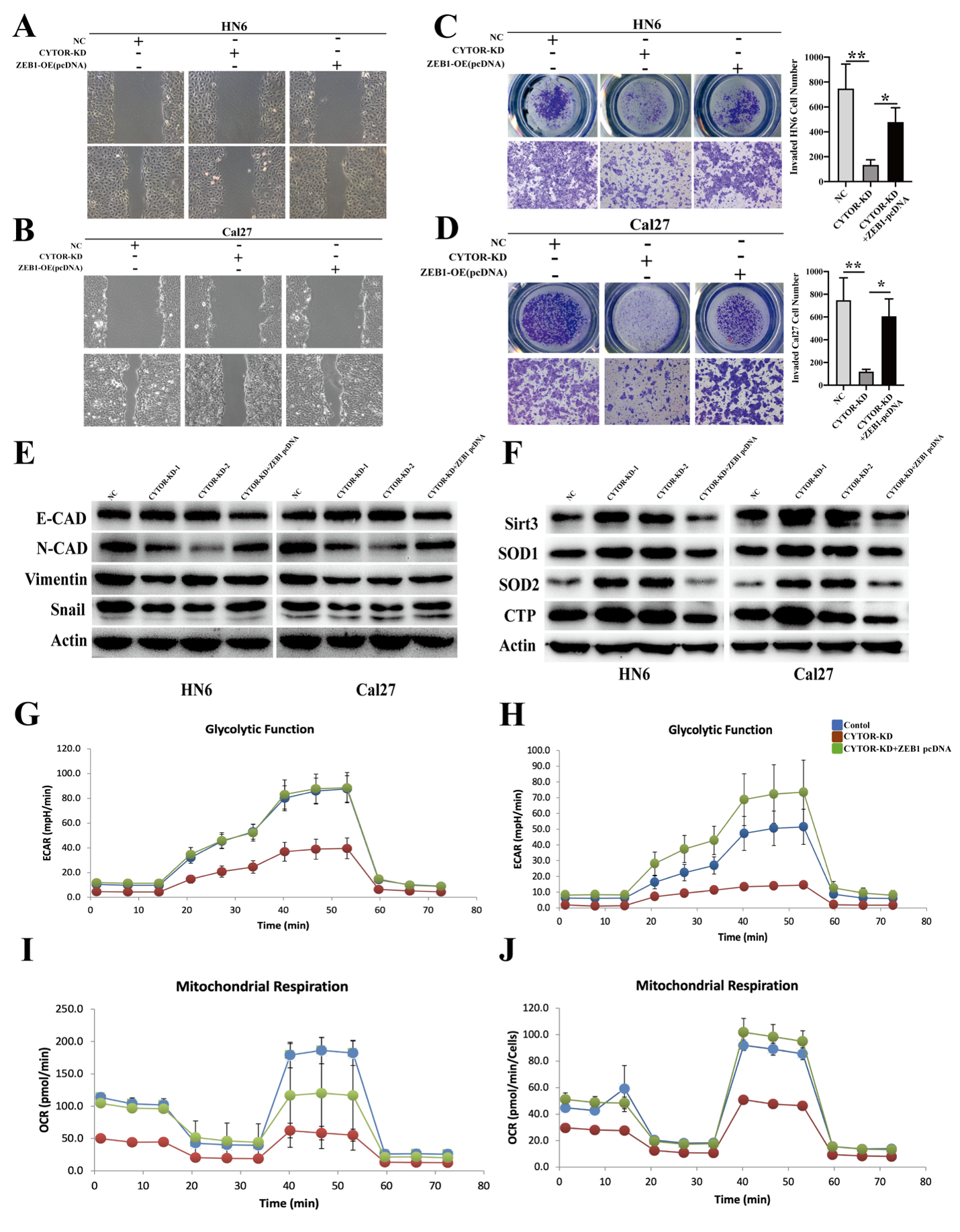

Supplement: Supplementary file 12 — Supplementary Figure 11 [file 41419_2022_5157_MOESM12_ESM.png]

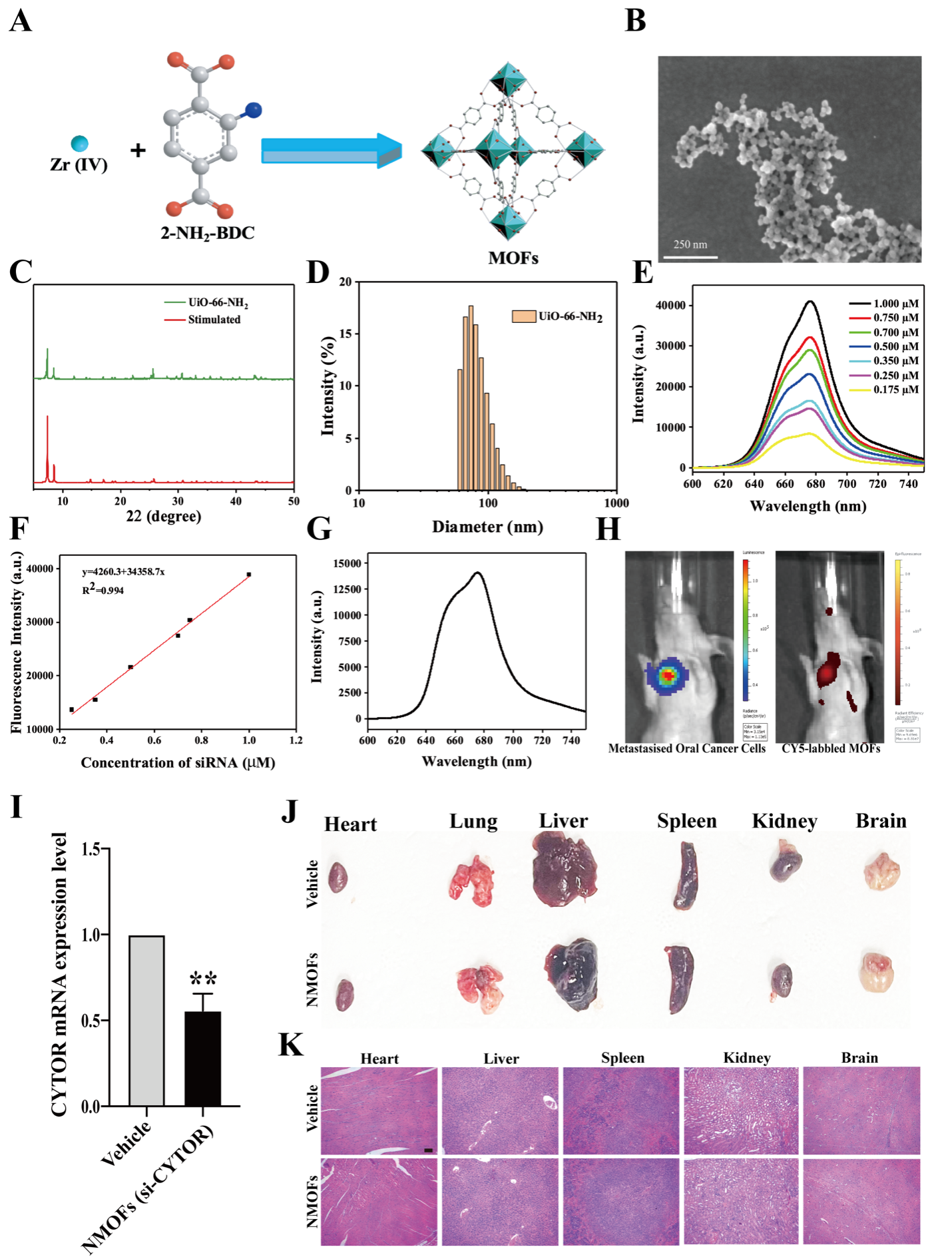

Supplement: Supplementary file 13 — Supplementary Figure 12 [file 41419_2022_5157_MOESM13_ESM.png]
