# Supplementary material for: lncRNA CYTOR promotes aberrant glycolysis and mitochondrial respiration via HNRNPC-mediated ZEB1 stabilization in oral squamous cell carcinoma
Source: Cell Death Dis. 2022 Aug 13;13(8):703. doi: 10.1038/s41419-022-05157-1 (PMC9376070; doi:10.1038/s41419-022-05157-1)
Supplement: Supplementary file 14 — Supplementary Figure Legends [file 41419_2022_5157_MOESM14_ESM.docx]

**Supplementary Figure Legends**

**Fig S1. lncRNA CYTOR is upregulated in HNSCC tissues and is** **indicative of poor prognosis.** (A) Volcano Plot showed the differentiated expressed lncRNAs in TCGA. (B) Volcano Plot of the differentiated expressed genes in GSE23558. (C) Venn diagram showed 6 up-regulated lncRNAs in both two datasets. (D) Kaplan-Meier analysis showed the overall survival curves of 6 lncRNAs in HNSCC patients. (E) qPCR analysis of lncRNA HOTAIRM1 abundance in OSCC tissues and normal tissues (n=32). (F) qPCR analysis showed that CYTOR was overexpressed in OSCC tissues, compared to the adjacent normal tissues (n=32). (G-H) CYTOR expression in HNSCC patients with different clinical stages and pathology grades. The data was presented as mean ± SD, *P < 0.05, **P < 0.01, ***P < 0.001.

**Fig S2. FISH assay of CYTOR expression in 192 OSCC patient tissues.** A tissue microarray with 192 OSCC tumor tissues was subjected to FISH assay to determine the expression level of CYTOR. Scale bar = 2000 μm.

**Fig S3. Identification of the subcellular location of CYTOR in oral cancer cells.** (A-B) The subcellular location of CYTOR in HN6 and Cal27 was determined by FISH, scale bar = 50 μm. (C-D) Subcellular fractionation were carried out and both the nuclear and cytoplasm fractions were subjected to qPCR to determine the subcellular location of CYTOR in HN6 and Cal27 cell lines.

**Fig S4. CYTOR promotes the EMT but does not affect the proliferation of OSCC cell lines.** (A-D) The expression level of CYTOR in HN6 and Cal27 cell line. β-actin served as the internal control. (E-F) EdU assay was used to assess the proliferation of HN6 and Cal27 cell lines. scale bar = 50 μm. (G-H) A CCK-8 assay was performed to determine the effect of CYTOR expression in regulating the proliferation of OSCC cells. (I) The stable CYTOR knockdown and overexpression cells were subjected to immunofluorescent to detect the expression of EMT-related markers (E-Catherin and Vimentin) (KD, knockdown; NC, negative control; OE, overexpression; scale bar, 50 μm. The data was presented as mean ± SD, ***P < 0.001).

**Fig S5. The deficiency of CYTOR reduces the metastasis of OSCC cells in nude mice.** (A) Nude mice were injected with the luciferase-labeled NC and CYTOR-KD oral cancer cells into the tail vein, then, lung metastasized cells were measured at day 45 post-injection. (B) Representative images of IHC and H&E staining of metastasized cells in lungs. (KD, knockdown; NC, negative control; Scale bars = 100 μm).

**Fig S6. MS evidence for ubiquitination site location in HNRNPC peptides.** Spectra were obtained from LC-MS/MS analyses using collision-induced dissociation (CID) at 35% and identified in MaxQuant 1.6.0.16. All modified residues can be confidently identified by confirming ions. Best evidence spectra were selected for annotation of b-ion (blue) and y-ion (red) series and their masses for singly- and doubly-charged fragments, identified ubiquitination of lysine residues were marked as k (gl).

**Fig S7. CYTOR mediates the non-degrative ubiquitination of HNRNPC.** (A-B) CYTOR-overexpressing and CYTOR-knockdown HN6 and Cal27 cells were treated with cycloheximide (CHX, 50 μg/mL) for the indicated times. (C) HN6 and Cal27 cells were treated with MG132 (25 μmol/l) for 12 h, and the HNRNPC abundance in the indicated cells were determined by immunoblotting. (D-E) The efficacy of CYTOR knockdown using small interferon RNA in 293T cells was determined by qPCR. (F-G) HN6 and Cal27 cells were transfected with indicated plasmids and siRNAs to study the deletion of CYTOR in regulating the ubiquitination level of HNRNPC. Cells were transfected with indicated plasmids or pcDNA 3.1 plasmids to overexpress the expression of CYTOR and followed by immunoprecipitation (IP), the immunoblotting for HA-Ub showed the ubiquitination of HNRNPC protein in (H) 293T (I) HN6 and (J) Cal27 cell lines. The data was presented as mean ± SD, **P < 0.01, ***P < 0.001.

**Fig S8. HNRNPC promotes the migration and invasion of OSCC cells.** The small interferon RNAs were used to knockdown (KD) the expression level of HNRNPC in OSCC cells. (A) Wound healing assays were used to evaluate the expression of HNRNPC in regulating the migratory ability of HN6 and Cal27 cell lines. (B-D) Transwell invasion assays were used to determine the HNRNPC expression in affecting the invasion of oral cancer cells. The data was presented as mean ± SD, *P < 0.05, **P < 0.01.

**Fig S9. Prediction of the binding possibility using CatRAPID.** (A) CatRAPID signature module prediction of the RNA-binding propensity for HNRNPC protein followed by the prediction of RNA-binding regions. Overall interaction scores above 50% indicate the binding propensity. (B) Binding prediction results of ZEB1 and LIN28B with HNRNPC protein. (C) CatRAPID fragment module prediction of the interaction profile and matrix between HNRNPC and ZEB1. (D) The predicted binding matrix map of HNRNPC and LIN28B.

**Fig S10. The identification of the efficiency for ZEB1 overexpression and knockdown.** (A) The mRNA expression level of ZEB1 in HN6 and Cal27 cell lines. (B) The protein expression level of ZEB1 was determined by western blot. (KD, knockdown; NC, negative control; OE, overexpression; The data was presented as mean ± SD, *P < 0.05, **P < 0.01).

**Fig S11. The ZEB1 overexpression rescues the CYTOR-KD induced phenotypes of OSCC cell lines.** The pcDNA 3.1 plasmids were used to overexpress the ZEB1 expression level in CYTOR-KD cells. (A-B) Wound healing assays were used to evaluate the expression of ZEB1 in rescuing the CYTOR-KD induced migration inhibition. (C-D) Transwell invasion assays were used to determine the invasion of oral cancer cells. (E) The HN6 and Cal27 cells were treated with the indicated plasmids and the epithelial mesenchymal transition (EMT) status was determined by western blot. (F) Western blot analysis showing the mitochondrial-related proteins (SIRT3, CTP, SOD1, SOD2) expression in HN6 and Cal27 cells. (G and I) SCC9 and (H and J) SCC25 cells were detected for ECAR and OCR to indicate the glycolysis stress and mitochondrial respiration. The data was presented as mean ± SD, *P < 0.05, **P < 0.01.

**Fig S12. The synthesis of nano scaled-metal organic frameworks (NMOFs).** (A) The synthesis method of NMOFs (UiO-66-NH2), UiO-66-NH2 was obtained by solvothermal method of ZrCl4 and 2-aminoterephthalic acid (2-NH2-BDC). (B) Scanning electron microscope (SEM) image of UiO-66-NH2 showed that the diameter of UiO-66-NH2 was around 70 nm, due to the enhanced permeability and retention (EPR) effect, UiO-66-NH2 can be accumulated at the tumor site. (C) X-ray diffraction (XRD) patterns of UiO-66-NH2 showed similar XRD patterns compared to the stimulated. (D) Dynamic light scattering (DLS) image of UiO-66-NH2 showed that the average hydrodynamic diameter of UiO-66-NH2 was 75 nm. (E) Fluorescence intensity of different concentration of CY5-labbeled si-RNAs were determined. (F) Correlation between si-RNA concentration and fluorescence intensity was analyzed with linear correlation. (G) Based on the equation, the si-RNA loading efficiency (LE) was calculated as LE = loaded siRNA / original siRNA✖️100%. The si-RNA loading efficiency of NMOFs was about 48.14%. (H) CY5-labbled si-CYTOR encapsuled NMOFs (UiO-66-NH2) were injected at day 21 post-injection of luciferase-labeled cancer cells. lung metastasized cells were measured at day 45 post-injection. The florescent image demonstrated the desired tumor accumulation ability of UiO-66-NH2. (I) The CYTOR knockdown efficiency of NMOFs was determined by qPCR. (J) Overview of the main organs from vehicle- and NMOFs-treated nude mice. (K) H&E staining of the main organs from vehicle- and NMOFs-treated nude mice, Scale bar = 100 μm; The data was presented as mean ± SD, **P < 0.01.
