# Supplementary material for: lncRNA CYTOR promotes aberrant glycolysis and mitochondrial respiration via HNRNPC-mediated ZEB1 stabilization in oral squamous cell carcinoma
Source: Cell Death Dis. 2022 Aug 13;13(8):703. doi: 10.1038/s41419-022-05157-1 (PMC9376070; doi:10.1038/s41419-022-05157-1)

Figure 1J

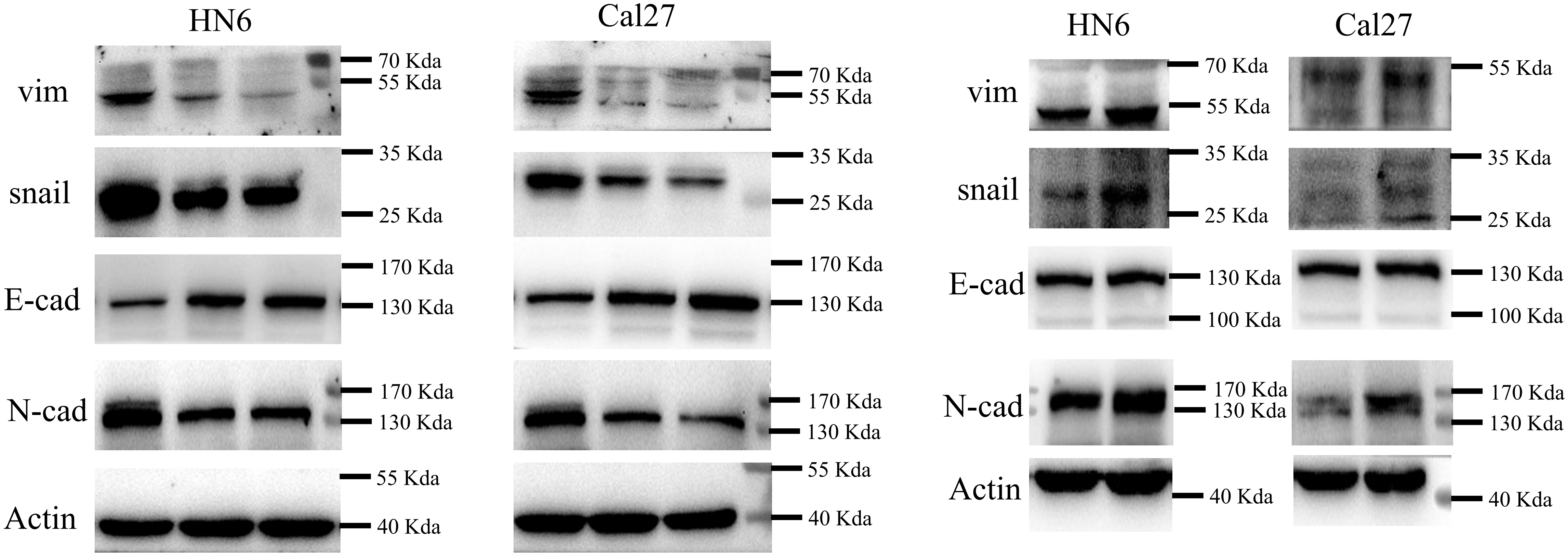

HN6
Cal27
HN6
Cal27
vim
snail
E-cad
N-cad
Actin
70 Kda
55 Kda
35 Kda
25 Kda
170 Kda
130 Kda
100 Kda
40 Kda

Figure 1K

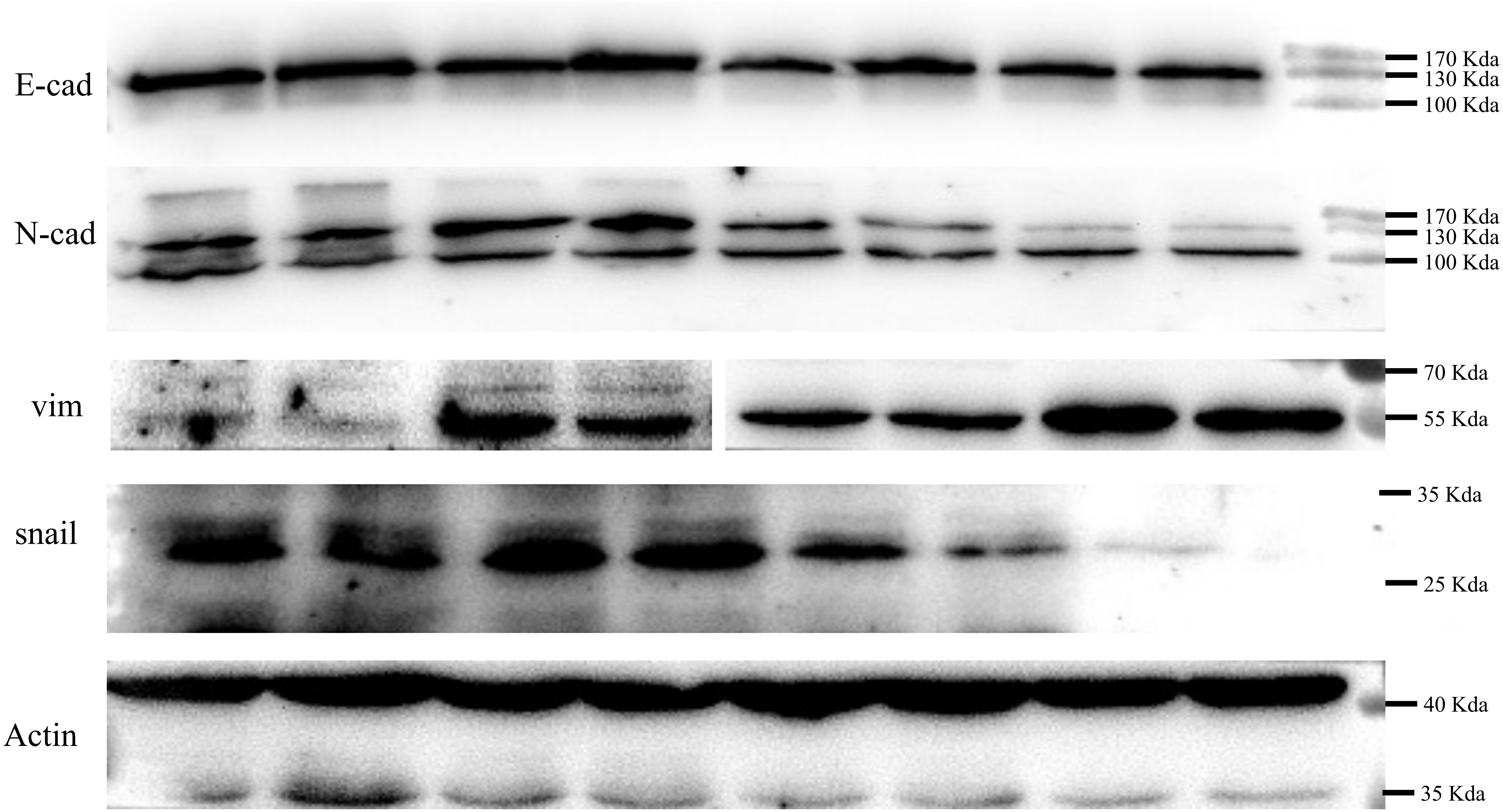

E-cad
N-cad
vim
snail
Actin
170 Kda
130 Kda
100 Kda
170 Kda
130 Kda
100 Kda
70 Kda
55 Kda
35 Kda
25 Kda
40 Kda
35 Kda

Figure 2C

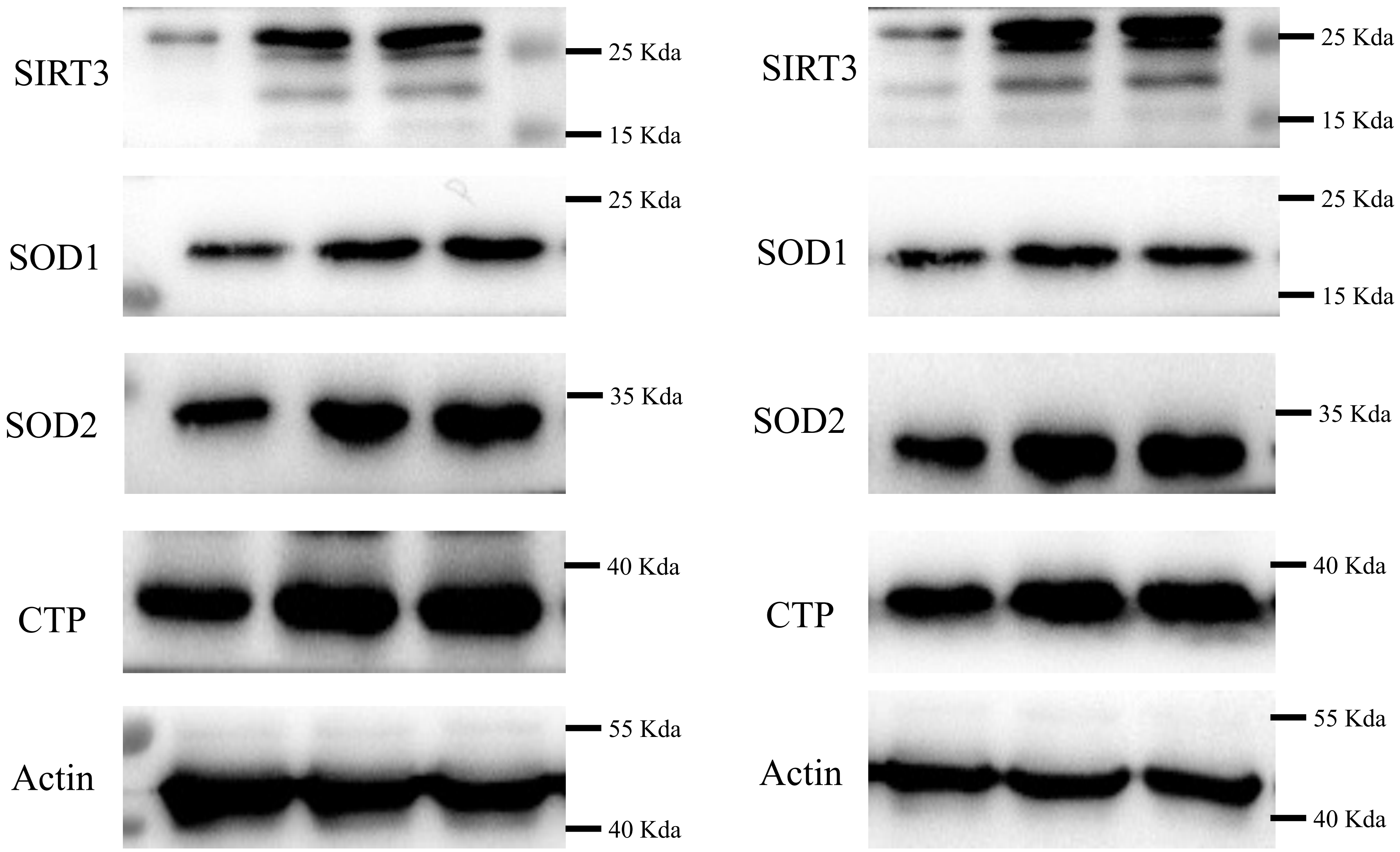
SIRT3
25 Kda
15 Kda
SOD1
25 Kda
SOD2
35 Kda
CTP
40 Kda
Actin
55 Kda
40 Kda
SIRT3
25 Kda
15 Kda
SOD1
25 Kda
15 Kda
SOD2
35 Kda
CTP
40 Kda
Actin
55 Kda
40 Kda

Figure 3B

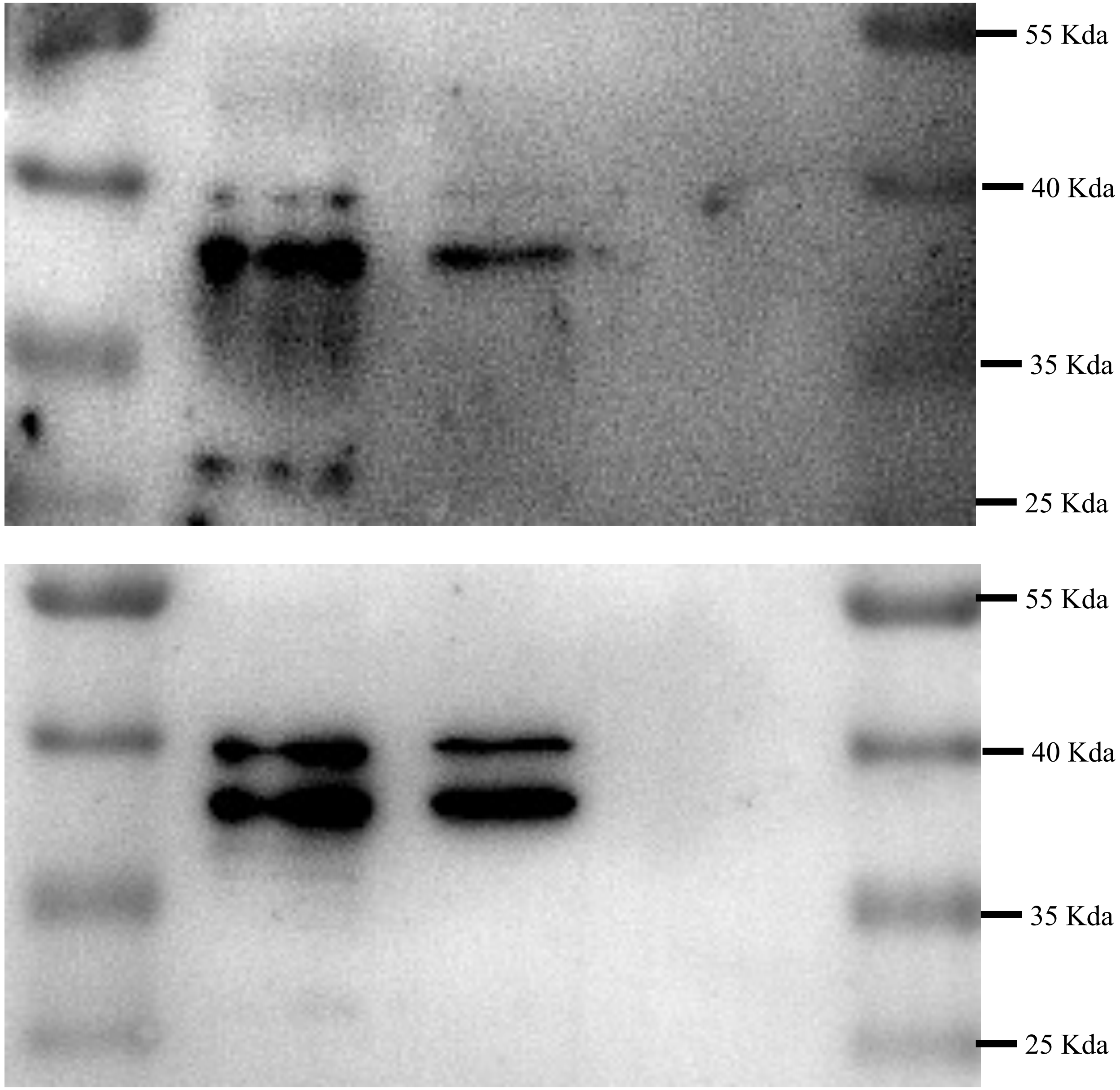

Figure 3E

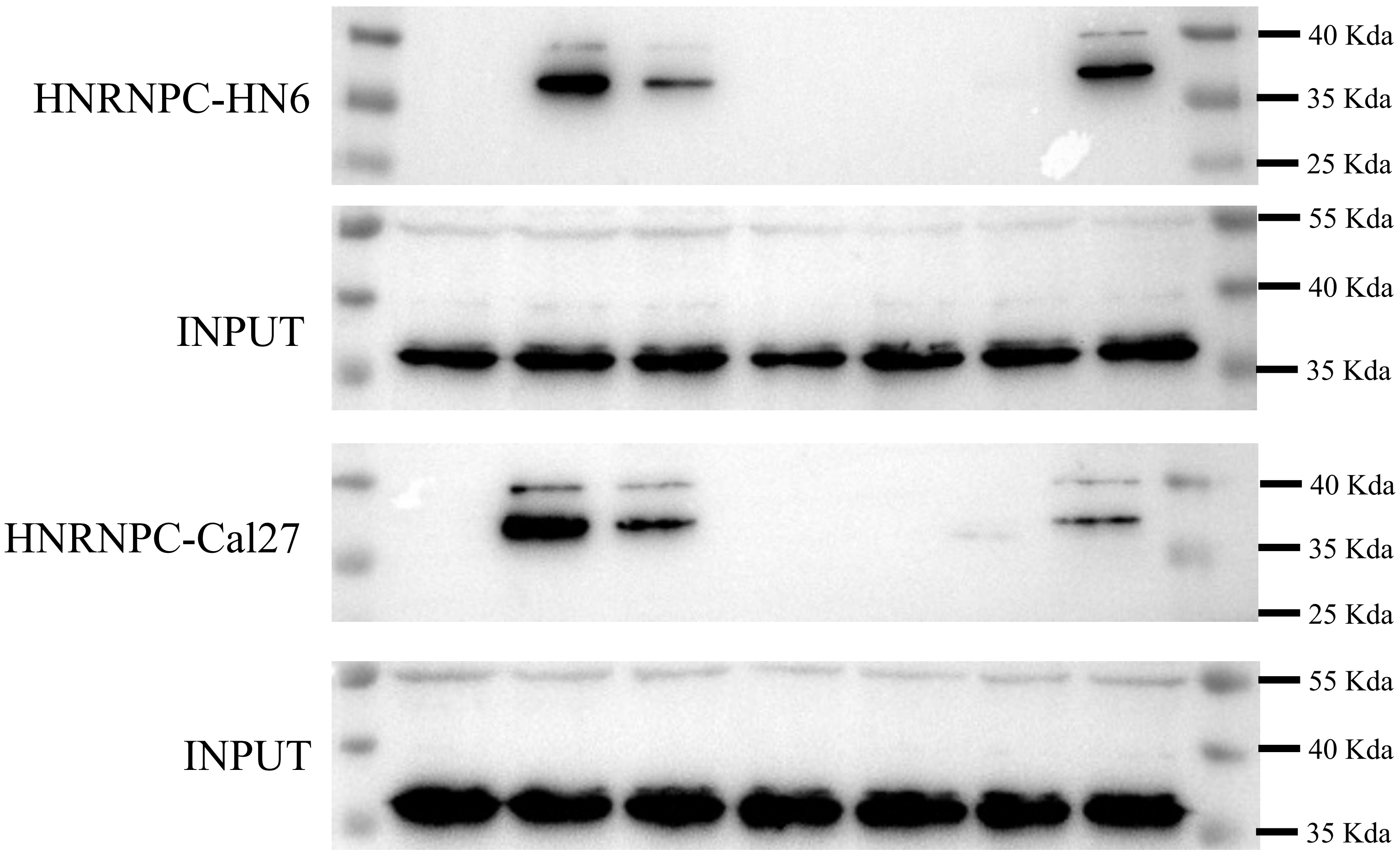

HNRNPC-HN6
40 Kda
35 Kda
25 Kda
INPUT
55 Kda
40 Kda
35 Kda
HNRNPC-Cal27
40 Kda
35 Kda
25 Kda
INPUT
55 Kda
40 Kda
35 Kda

Figure 3F

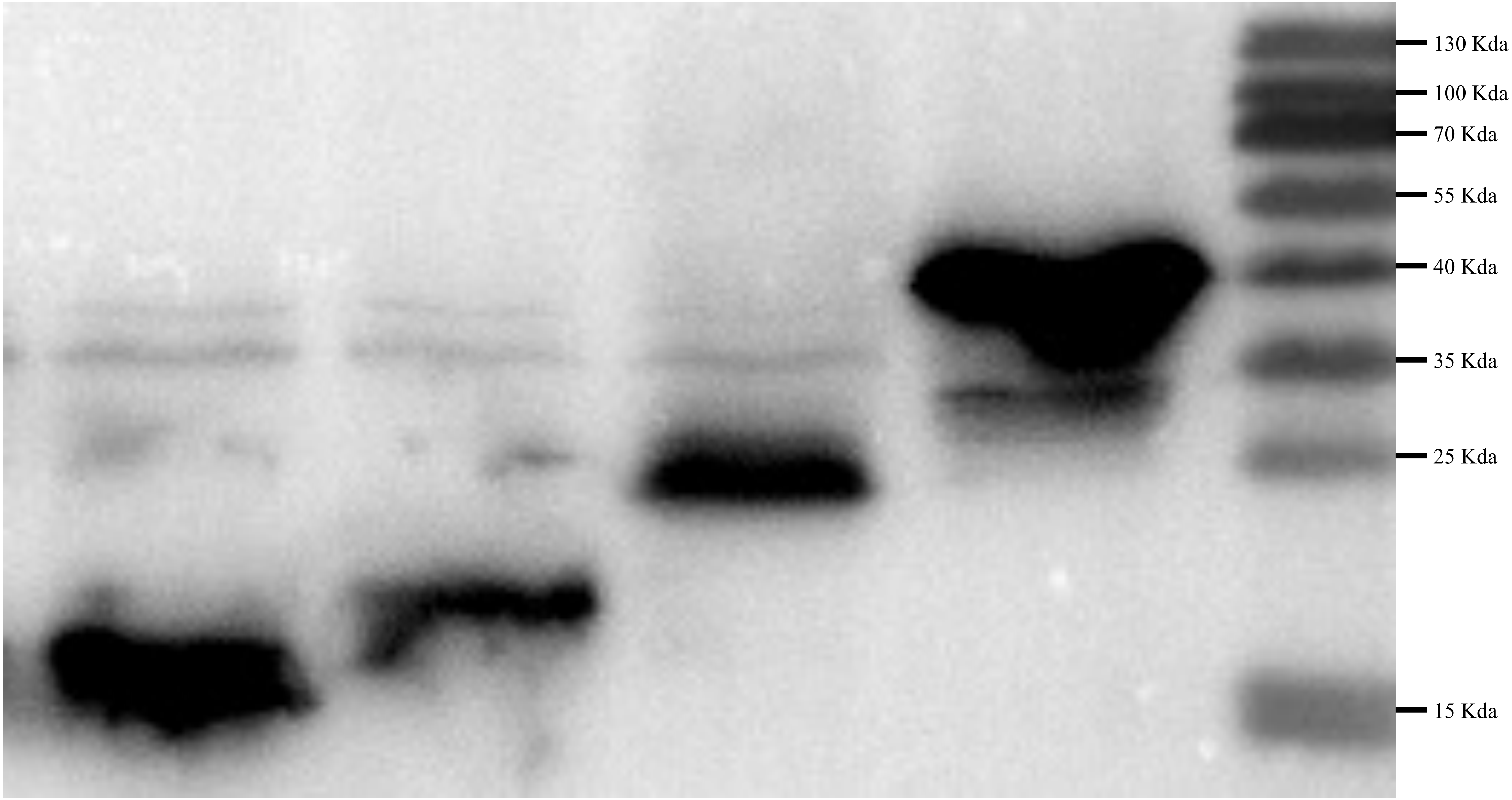

Figure 3I

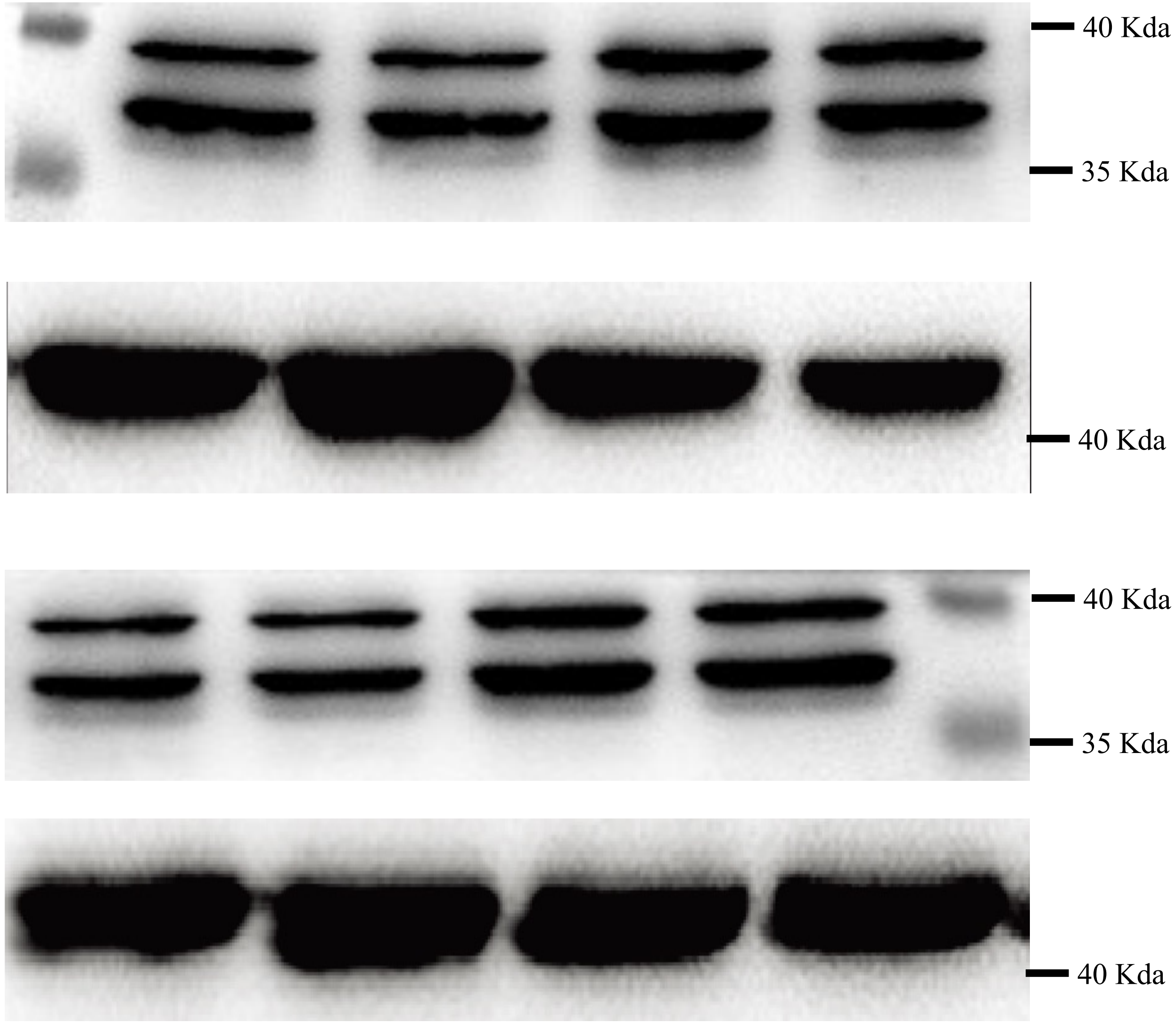

Figure 3K

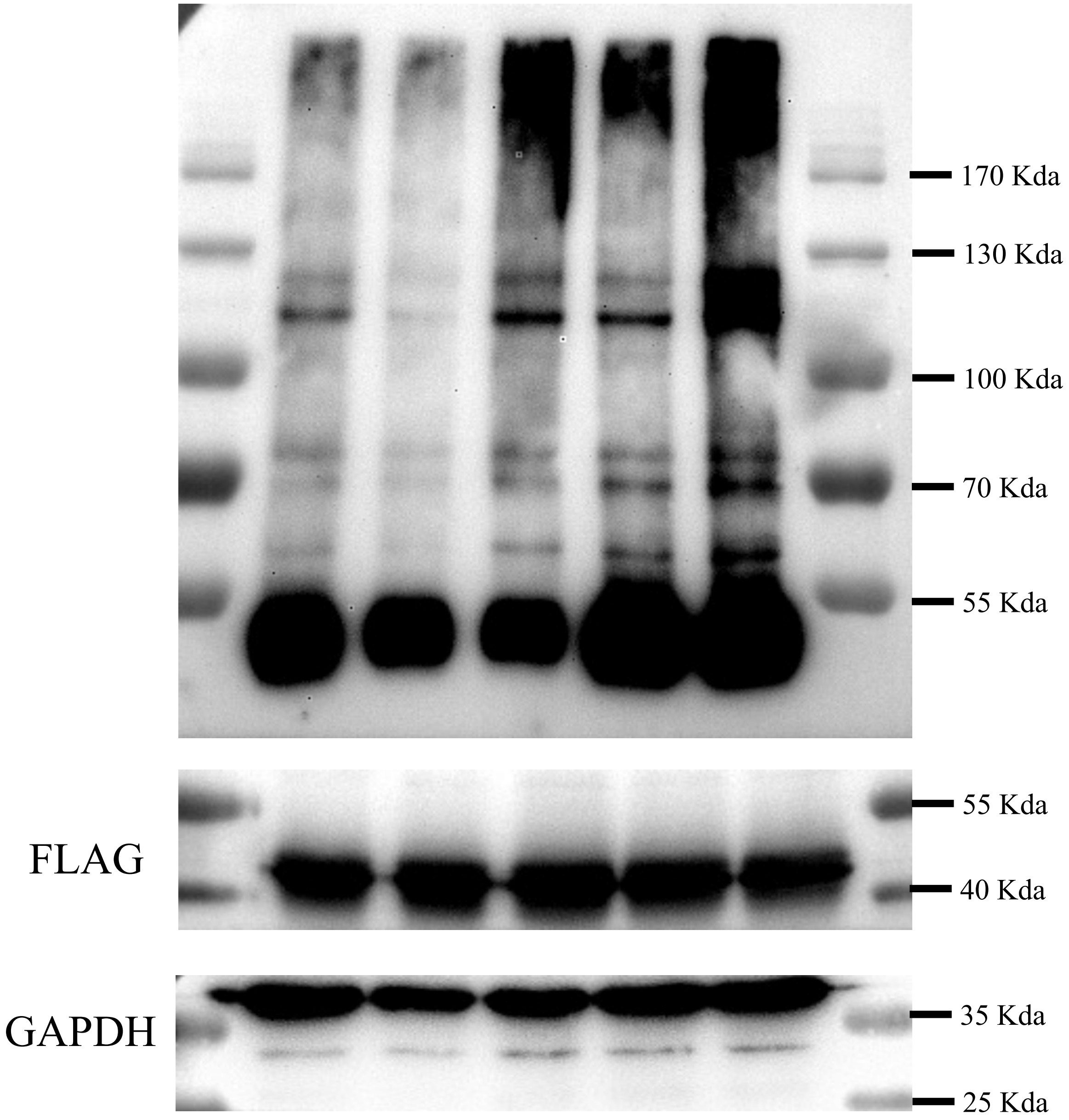
170 Kda
130 Kda
100 Kda
70 Kda
55 Kda
FLAG
55 Kda
40 Kda
GAPDH
35 Kda
25 Kda

Figure 3L

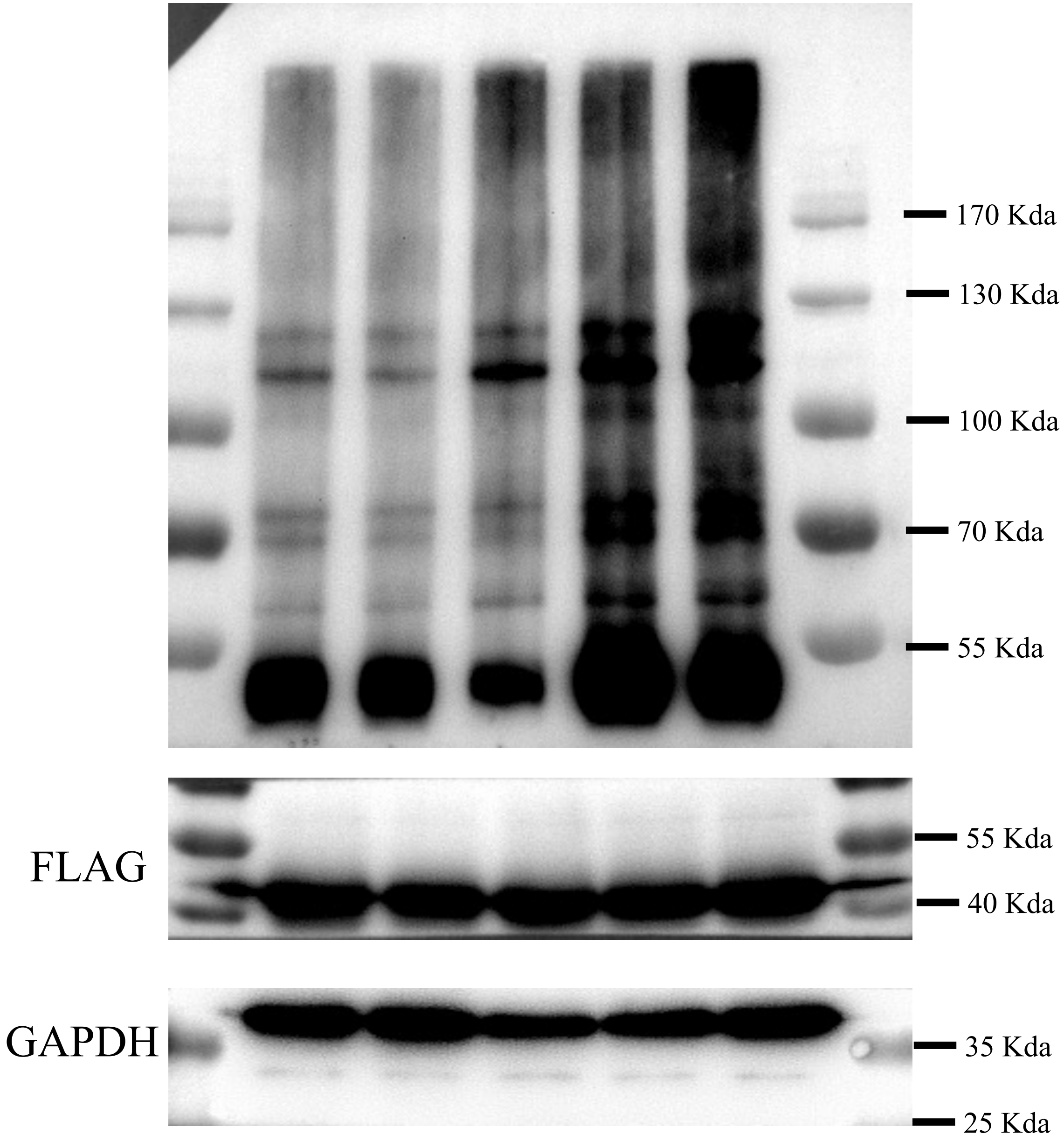

170 Kda
130 Kda
100 Kda
70 Kda
55 Kda
FLAG
55 Kda
40 Kda
GAPDH
35 Kda
25 Kda

Figure 3M

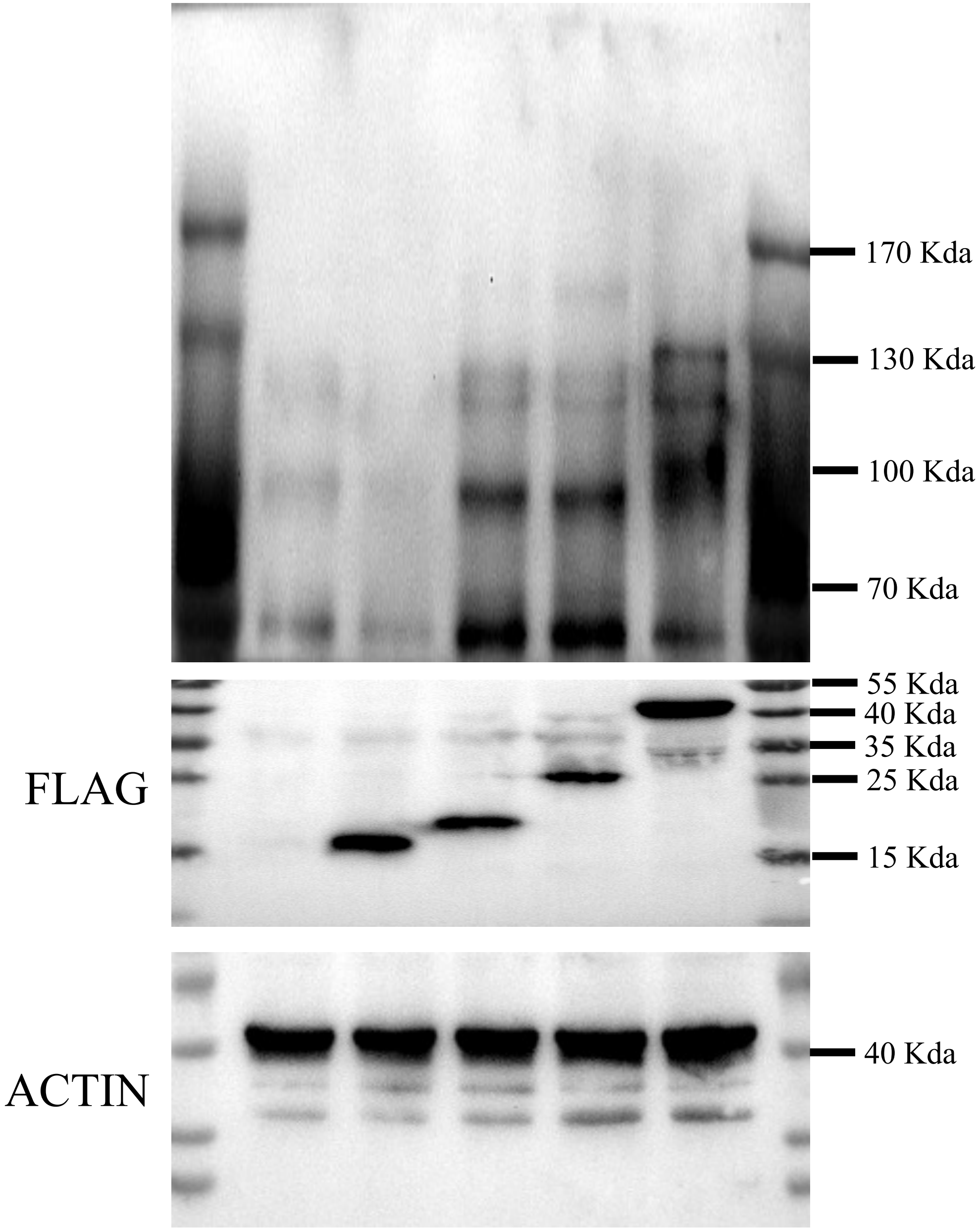
170 Kda
130 Kda
100 Kda
70 Kda
55 Kda
40 Kda
35 Kda
25 Kda
15 Kda
FLAG
ACTIN
40 Kda

Figure 4B

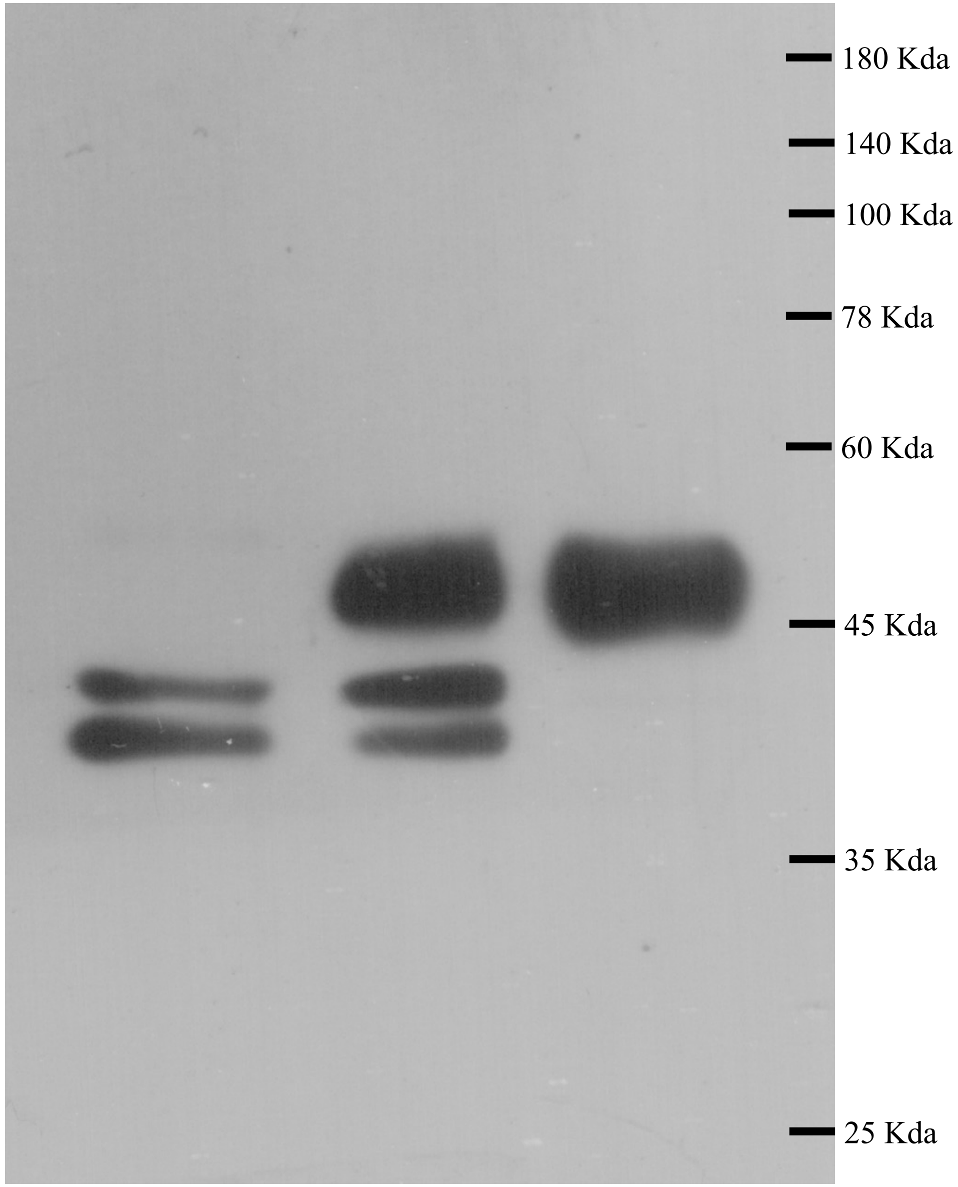

Figure 4E

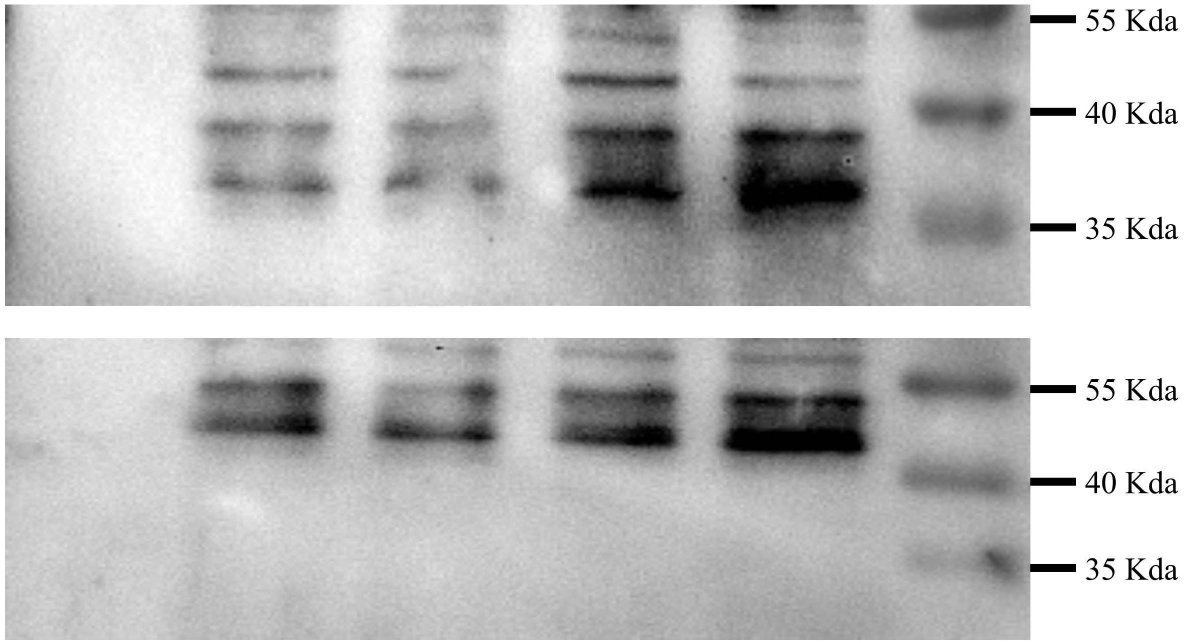

Figure 4F

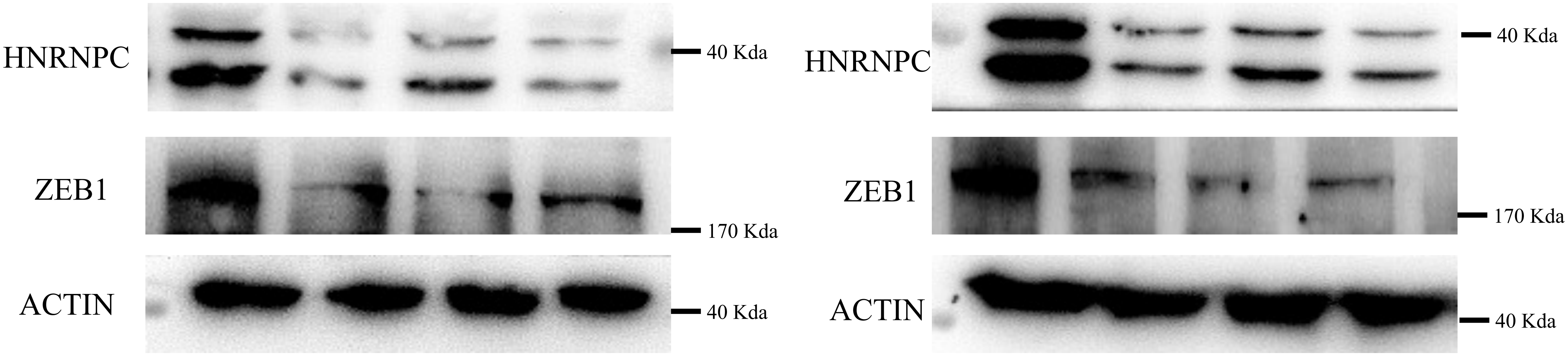
HNRNPC
40 Kda
ZEB1
170 Kda
ACTIN
40 Kda
HNRNPC
40 Kda
ZEB1
170 Kda
ACTIN
40 Kda

Figure 4G

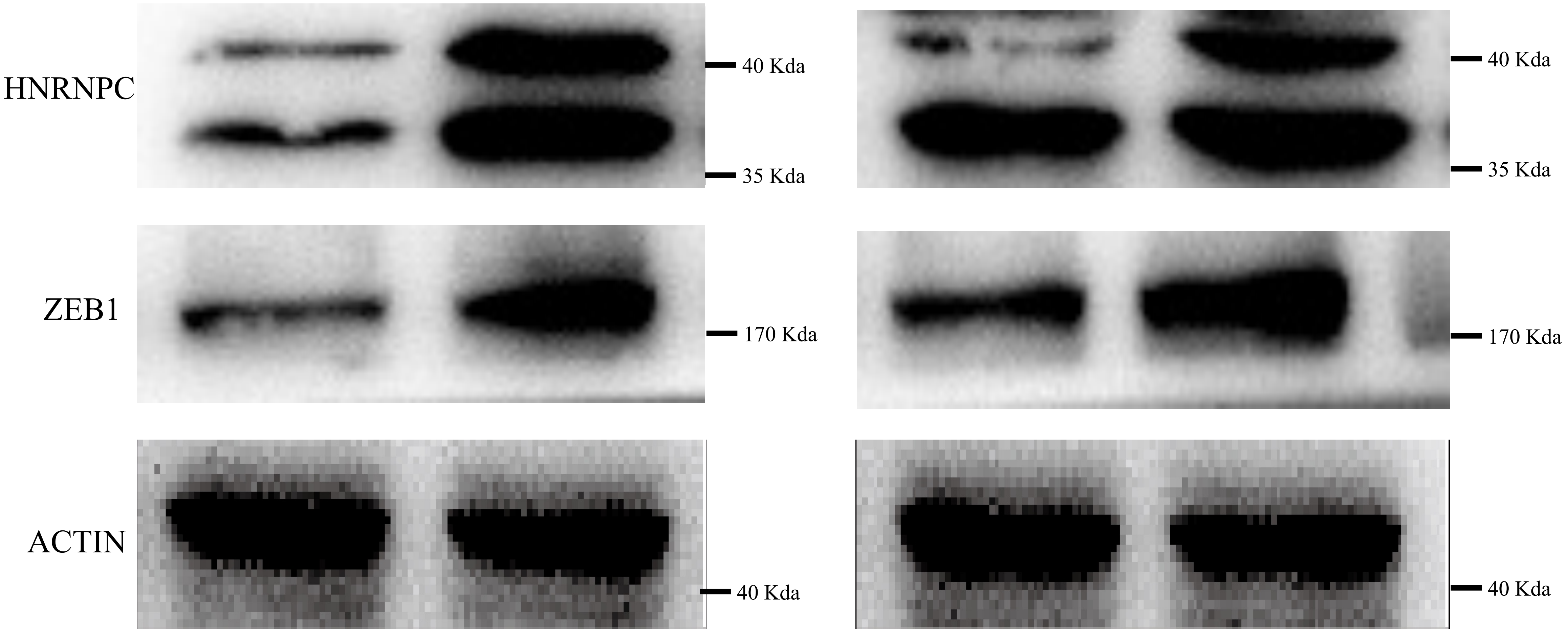
HNRNPC
ZEB1
ACTIN
40 Kda
35 Kda
170 Kda
40 Kda
40 Kda
35 Kda
170 Kda
40 Kda

Figure 4I

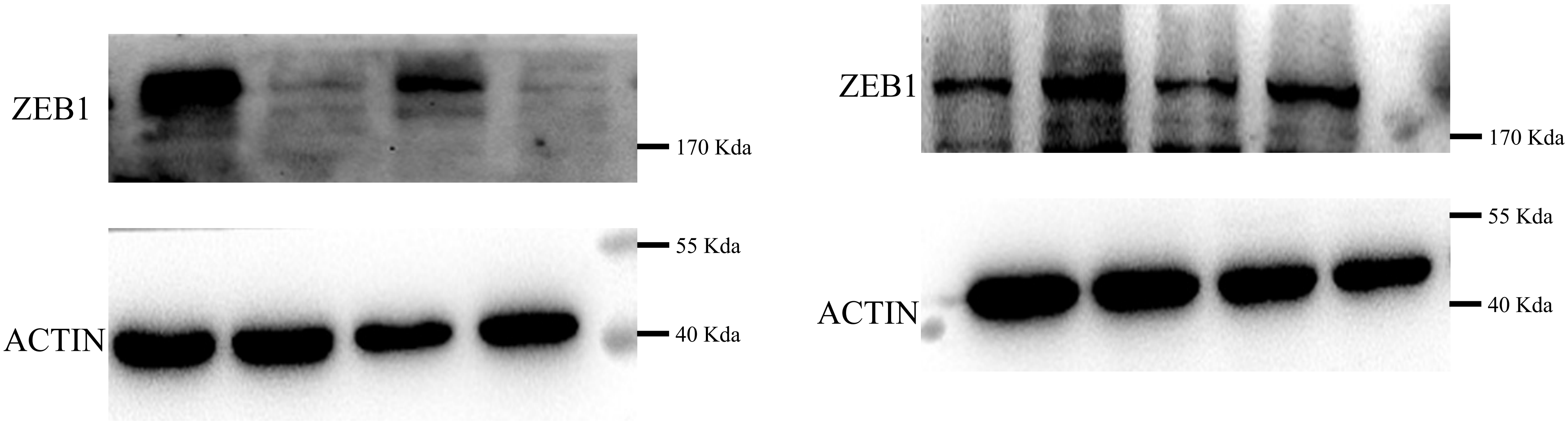
ZEB1
170 Kda
ACTIN
55 Kda
40 Kda
ZEB1
170 Kda
55 Kda
ACTIN
40 Kda

Figure 4J

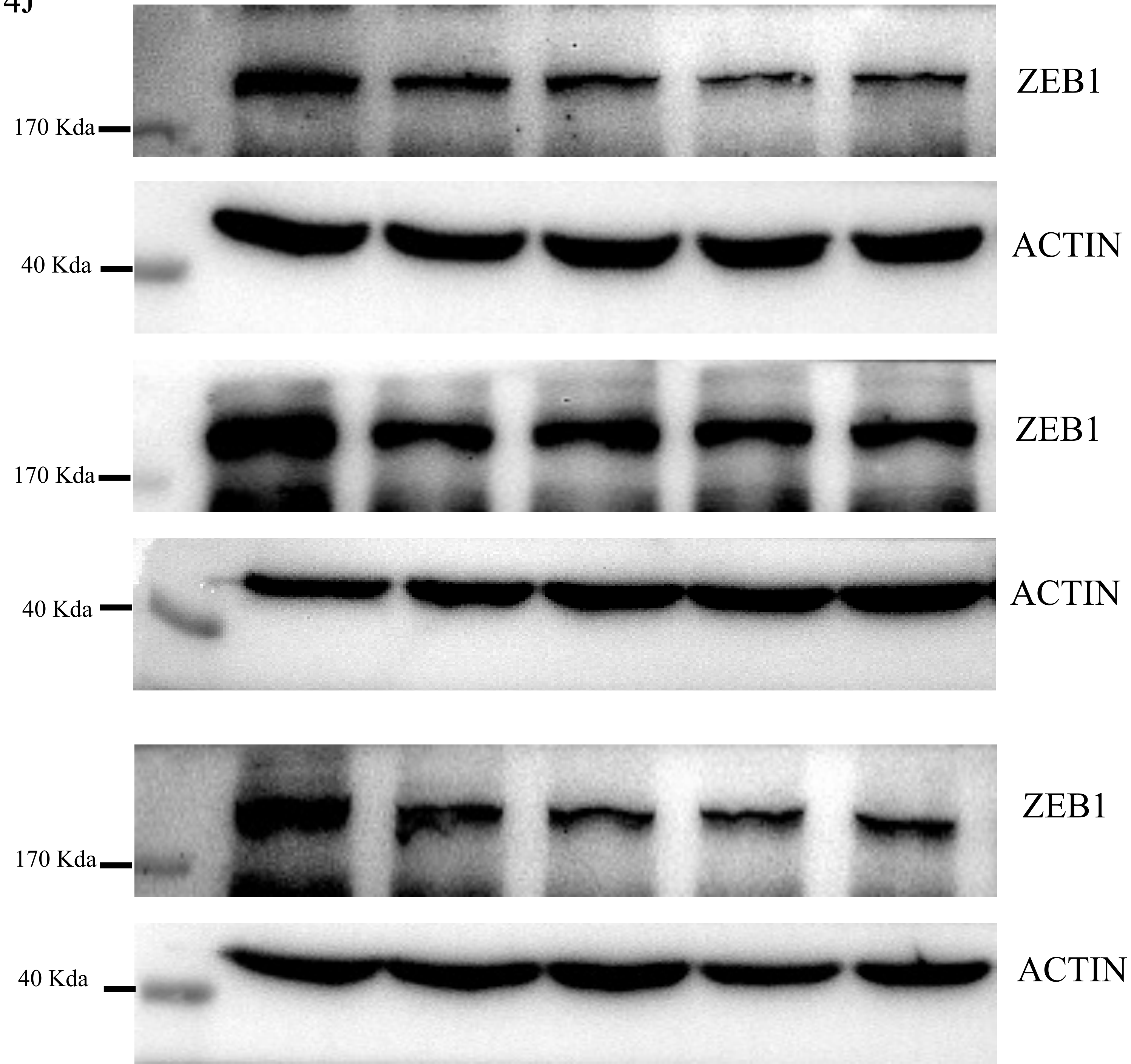
ZEB1
170 Kda
ACTIN
40 Kda
ZEB1
170 Kda
ACTIN
40 Kda
ZEB1
170 Kda
ACTIN
40 Kda

Figure 5F

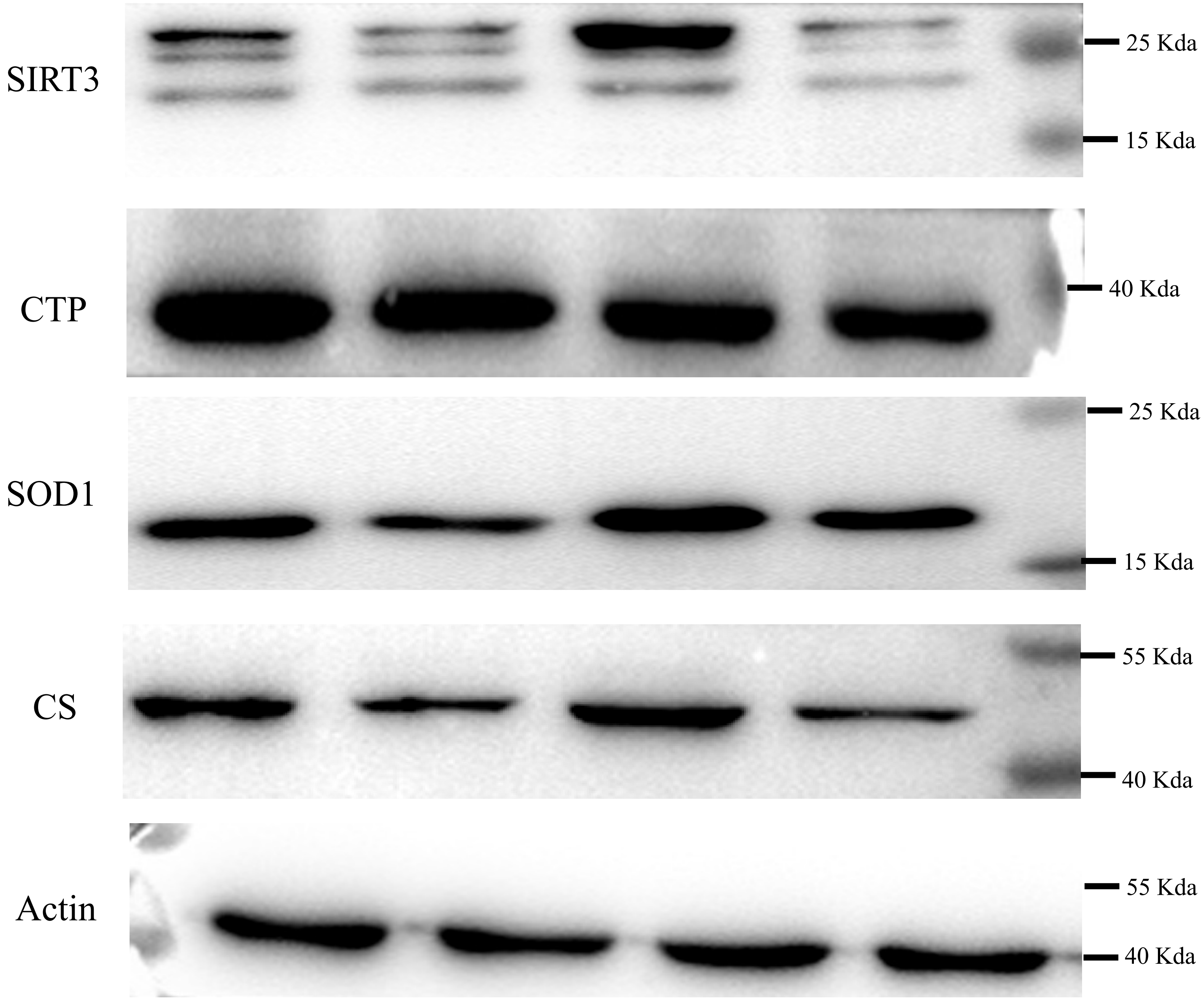

SIRT3
25 Kda
15 Kda
CTP
40 Kda
SOD1
25 Kda
15 Kda
CS
55 Kda
40 Kda
Actin
55 Kda
40 Kda

Figure 6B

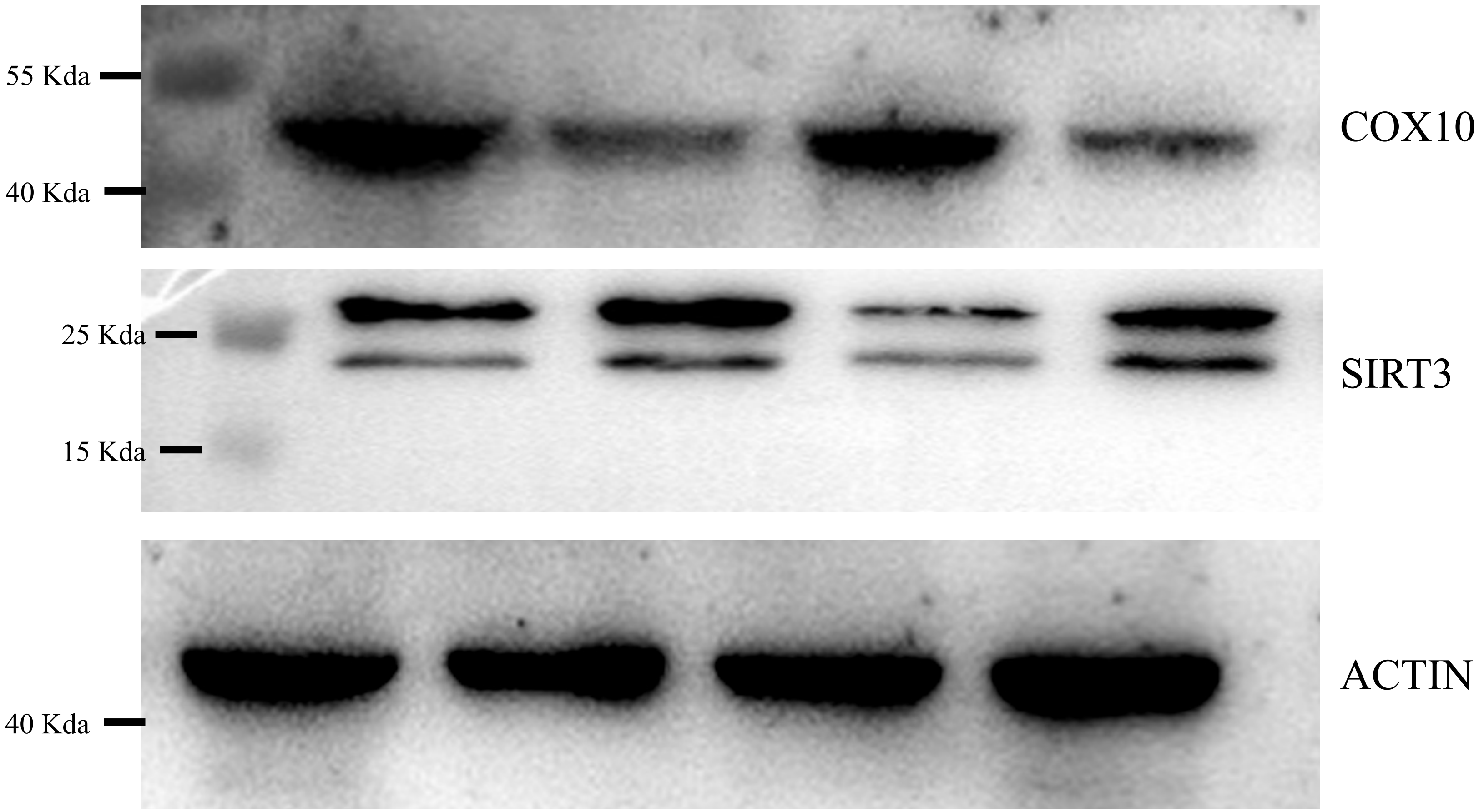

Supplementary Figure S7A-C

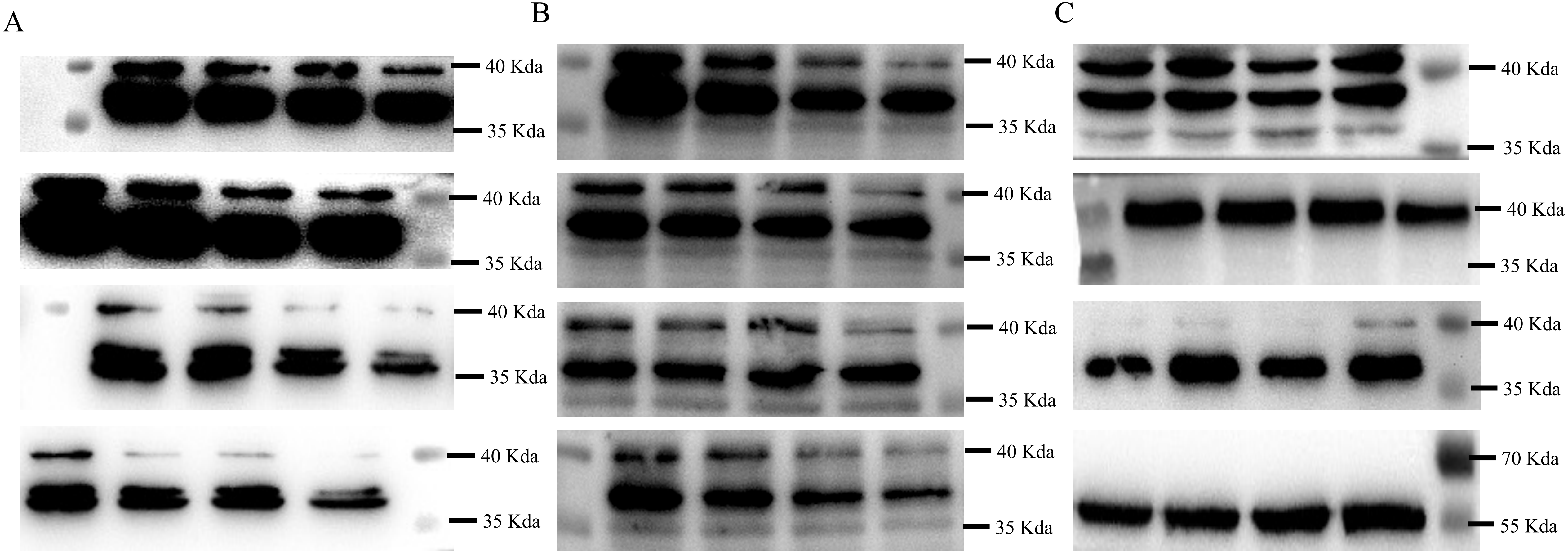

Supplementary Figure S7F-J

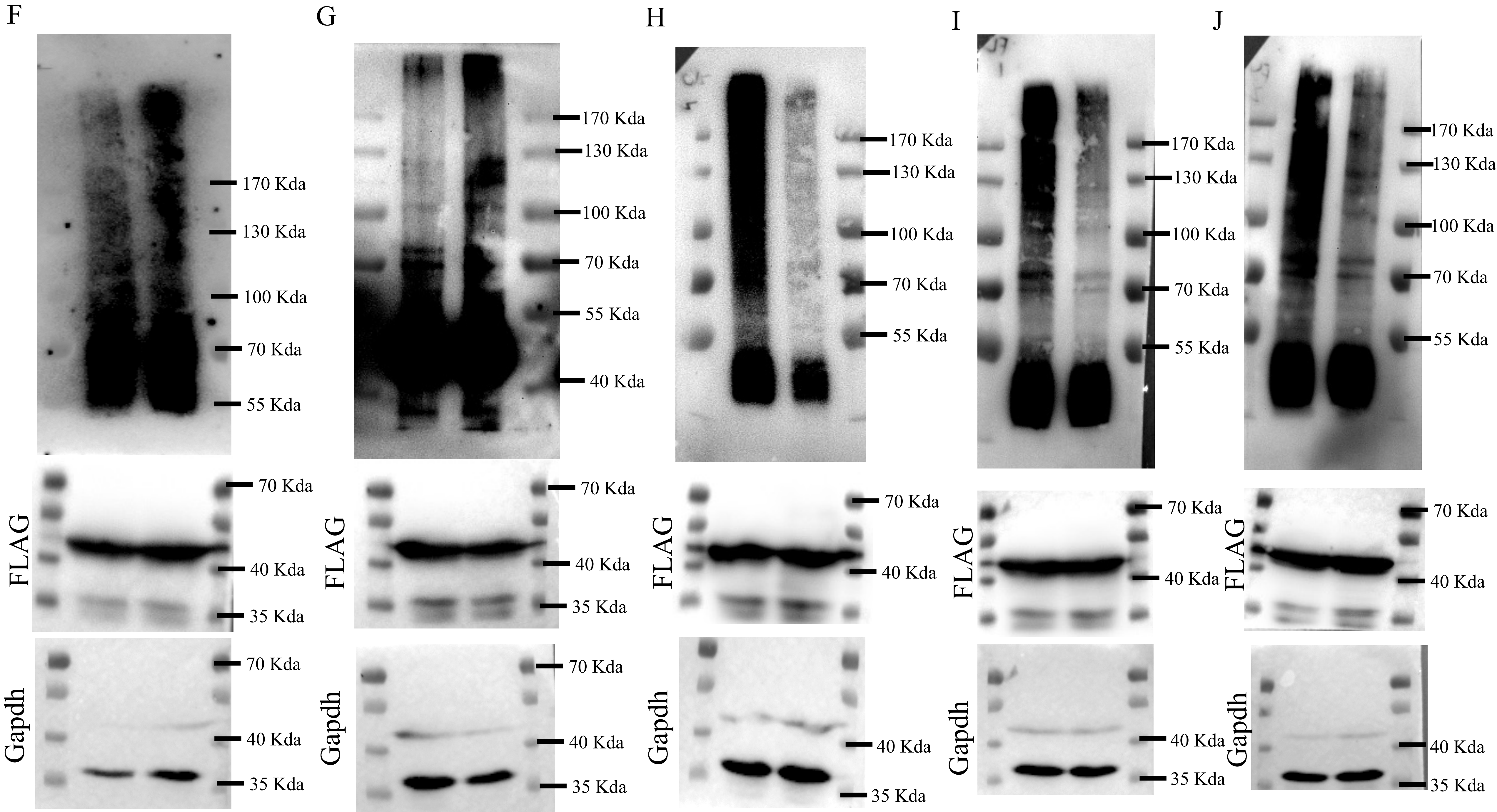

Supplementary Figure S10B

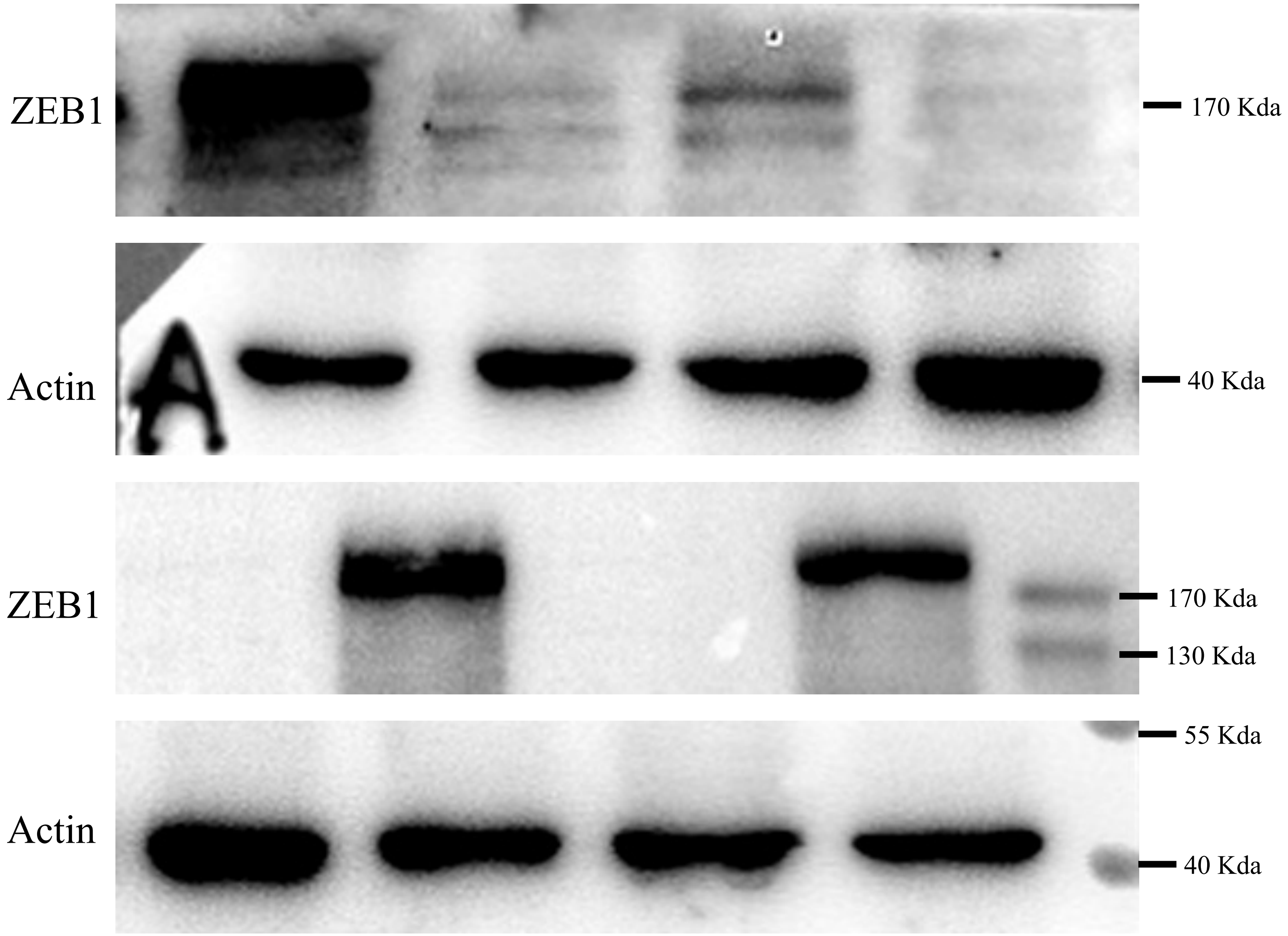

Supplementary Figure S11E

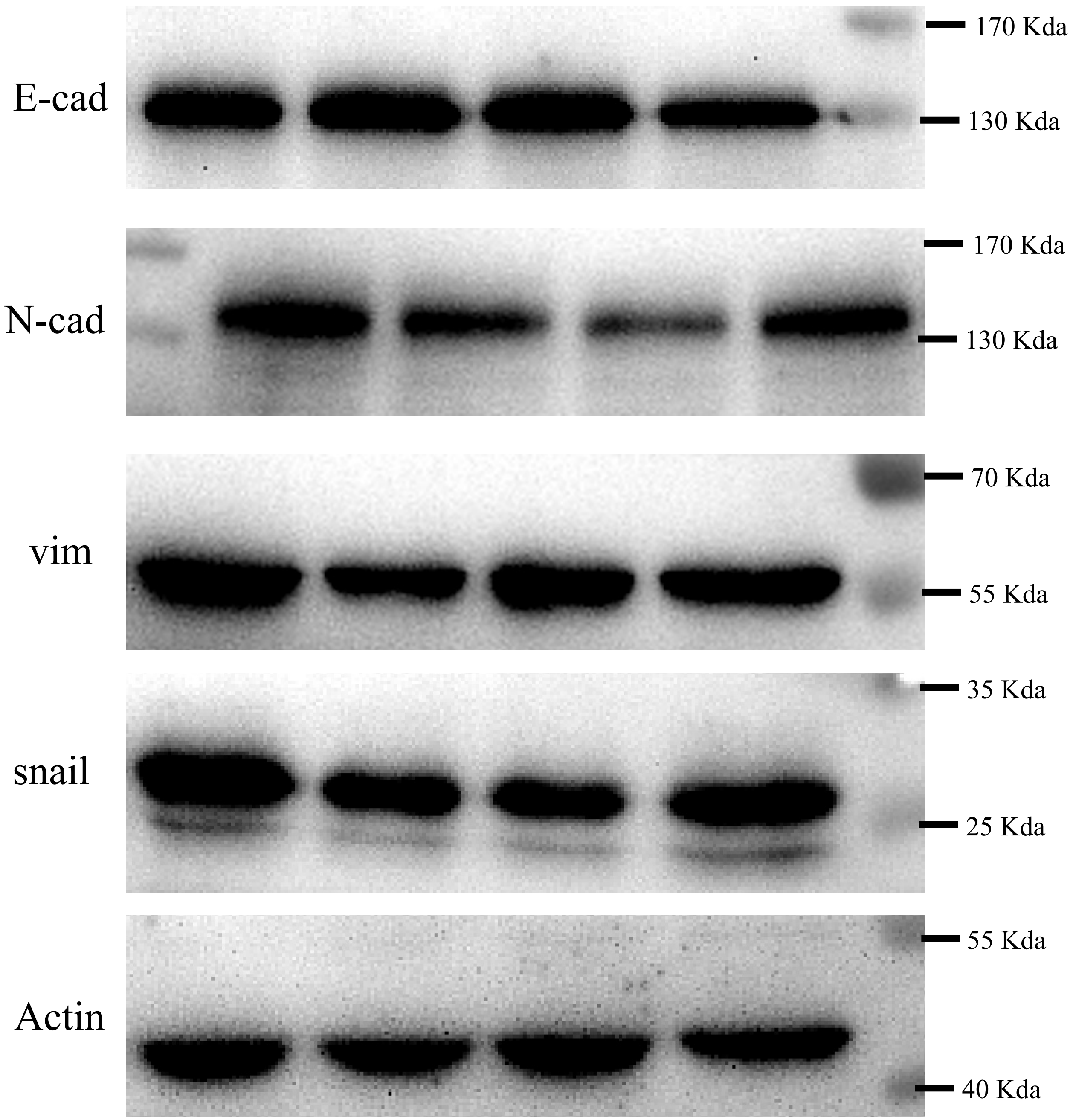

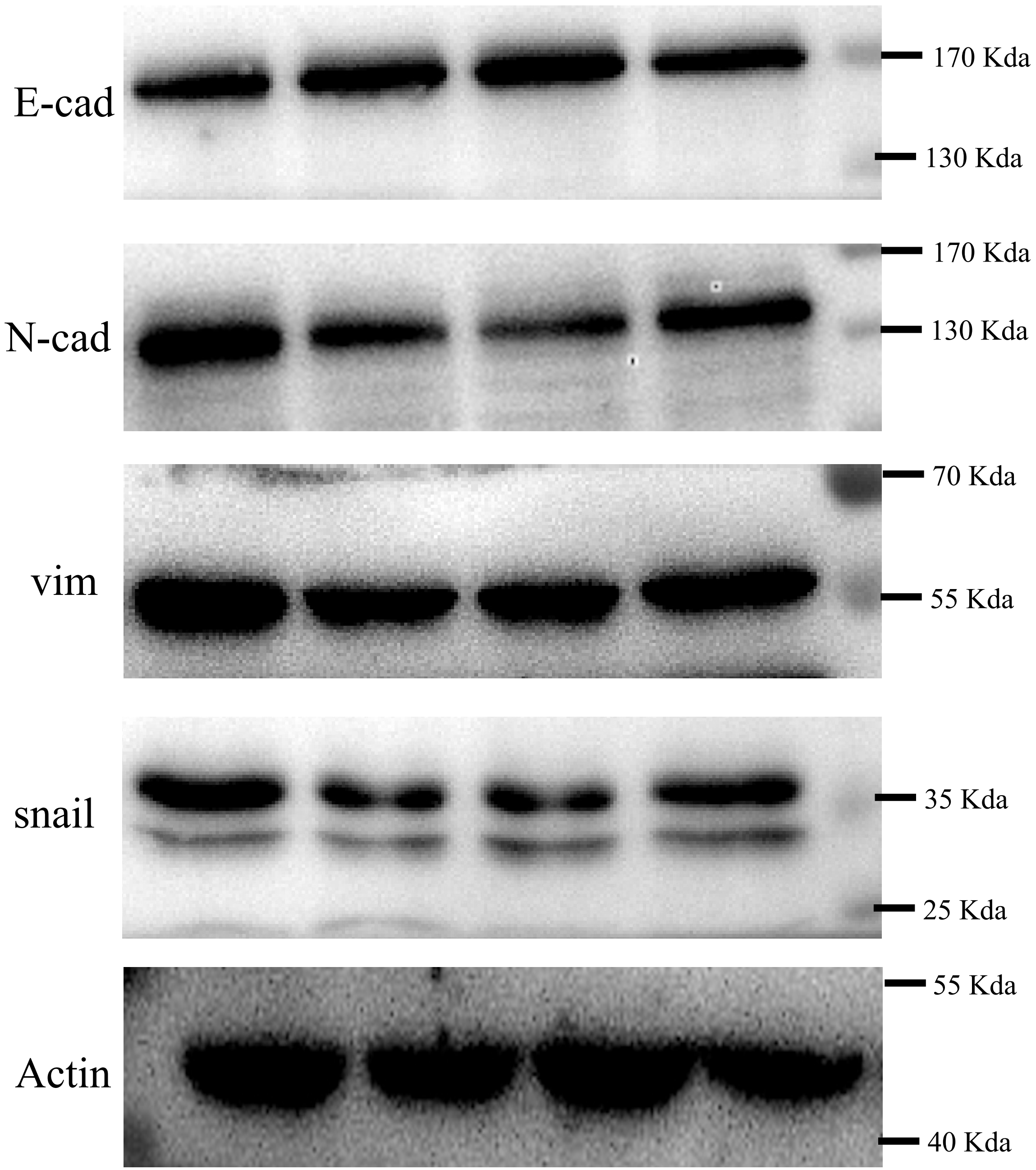

Supplementary Figure S11F

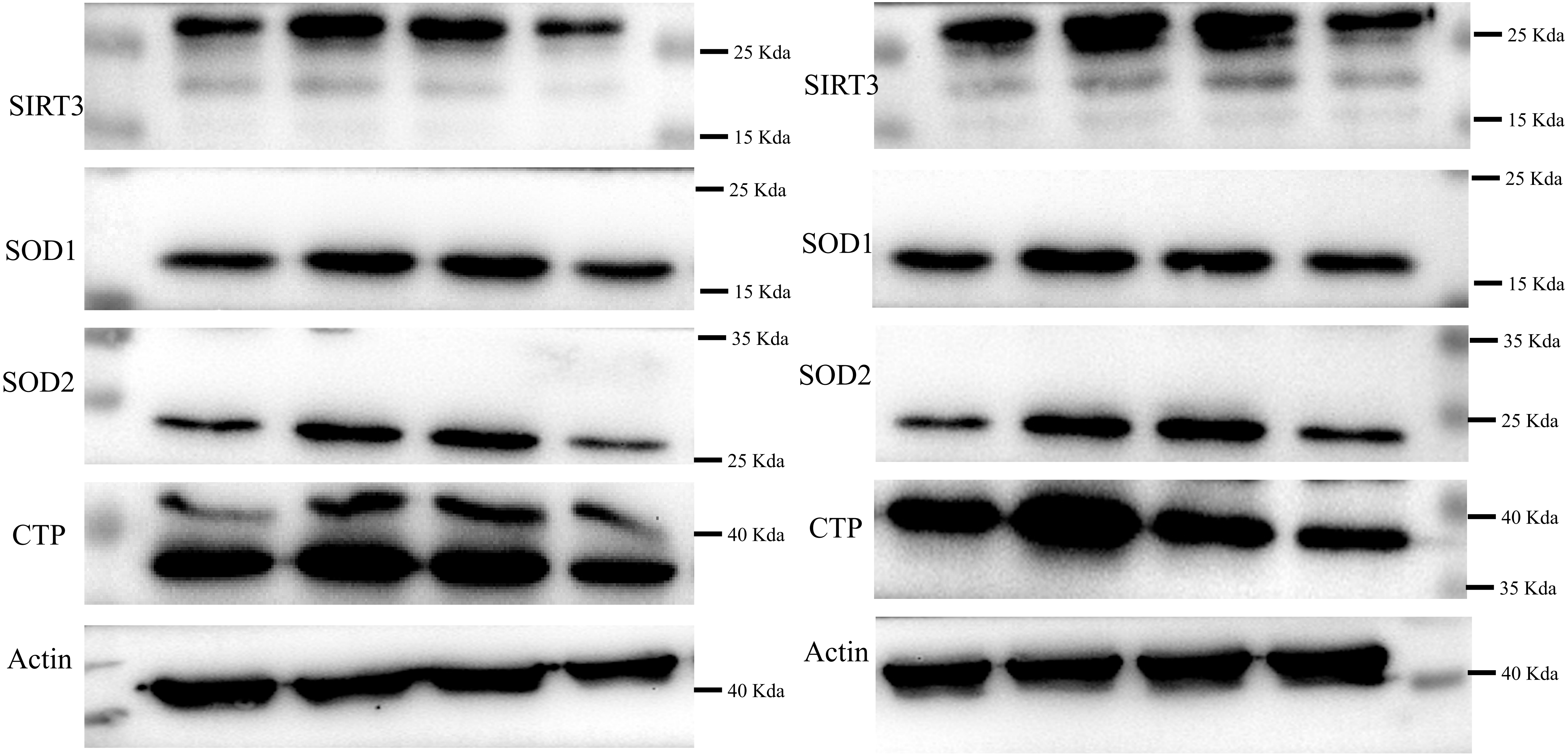

Supplement: Supplementary file 22 — Original Western Blots [file 41419_2022_5157_MOESM22_ESM.pdf]
